# Huoxiang Zhengqi Oral Liquid Attenuates LPS-Induced Acute Lung Injury by Modulating Short-Chain Fatty Acid Levels and TLR4/NF-*κ*B p65 Pathway

**DOI:** 10.1155/2023/6183551

**Published:** 2023-02-17

**Authors:** Ruying Tang, Jianjun Zhang, Rui Zhang, Xinyu Li, Ruilin Lv, Haipeng Nan, Jinlian Liu, Zhongpeng Zhao, Wei He, Linyuan Wang

**Affiliations:** ^1^School of Chinese Materia Medica, Beijing University of Chinese Medicine, Beijing, China; ^2^School of Traditional Chinese Medicine, Beijing University of Chinese Medicine, Beijing, China; ^3^School of Basic Medical Sciences, Anhui Medical University, Hefei, China

## Abstract

Huoxiang Zhengqi Oral Liquid (HZOL) is a classic Chinese patent medicine used in China for more than 1,000 years in treating gastrointestinal and respiratory diseases. Clinically applied HZOL in early respiratory disease stages can reduce the proportion of lung infection patients that progress to severe acute lung injury (ALI). However, few pharmacological studies evaluated its level of protection against ALI. We explored mechanisms of HZOL against ALI by employing network pharmacology, molecular docking, and rat experiments. Firstly, network pharmacology prediction and published biological evaluation of active ingredients of HZOL suggested that HZOL exerted the protective effect in treating ALI mainly in the areas of regulation of cell adhesion, immune response, and inflammatory response and closely related to the NF-*κ*B pathway. Secondly, molecular docking results demonstrated that imperatorin and isoimperatorin combined well with targets in the NF-*κ*B pathway. Finally, ALI rats induced by lipopolysaccharides (LPS) were used to validate prediction after pretreatment with HZOL for 2 weeks. Results confirmed that lung and colon injury occurred in ALI rats. Furthermore, HZOL exerts anti-inflammatory effects on LPS-induced ALI and gut injury by repairing lung and colon pathology, reducing and alleviating pulmonary edema, inhibiting abnormal enhancement of thymus and spleen index, modulating hematologic indices, and increasing levels of total short-chain fatty acids (SCFAs) in the cecum. Additionally, abnormal accumulation of inflammatory cytokines IL-6, IL-1*β*, TNF-*α*, and IFN-*γ* in serum and bronchoalveolar lavage fluid was significantly reduced after pretreating with HZOL. Furthermore, HZOL downregulated the expression of TLR4, CD14, and MyD88 and phosphorylation of NF-*κ*B p65 in lung tissue. Altogether, HZOL was found to exert an anti-inflammatory effect regulation by increasing levels of SCFAs, inhibiting the accumulation of inflammatory cytokines, and attenuating the activation of the TLR4/NF-*κ*B p65 pathway. Our study provided experimental evidences for the application of HZOL in preventing and treating ALI.

## 1. Introduction

Acute lung injury (ALI) has the pathophysiological characteristic of morphologic and functional lung abnormalities caused by the destruction of pulmonary capillaries and alveolar epithelia, resulting in severe inflammation in the lung [1, 2]. Acute lung injury (ALI), with the high mortality rate of 40%, is usually caused by various pathogenic factors, such as pathogenic microorganism infections. ALI is characterized by acute respiratory distress syndrome and refractory hypoxemia, accompanied by gastrointestinal dysfunction [3]. At the earliest stage of ALI, cytokine upregulation—represented by eradication or containment of invading microbes—and lung permeability increase, along with recruitment of inflammatory cells, followed by further aggravation if the ALI goes untreated [4]. As a result, lung inflammation and vascular hyperpermeability continue to worsen, often leading to acute respiratory distress syndrome (ARDS). Evidences from experimental studies have proved that through intraperitoneal injection, lipopolysaccharides (LPS) can induce not only acute lung injury but also intestinal injury [5]. Importantly, in the pathological state of LPS-induced inflammation and immune disorders in the ALI animal model, not only was an accumulation of inflammatory cytokine observed in the lung, but also short-chain fatty acid (SCFA) disbalance occurred in the cecum, demonstrating a close association in the lung-gut axis of ALI [6]. ALI can increase intestinal bacterial load, while it can decrease total SCFAs and individual SCFAs (acetic, propionic, butyric, valeric, and isobutyric acids) in the cecum [7]. Commonly, most SCFAs are produced in the cecum and proximal colon, which provides energy and nutrients for the microbiota. Especially, SCFAs are representative metabolites of the microbiota and are also associated with the lung and gut damage. It is an effective strategy to treat ALI from the lung-gut axis by inhibiting lung inflammation and modulating gut injury.

Clinically, aspirin, salbutamol, and ketoconazole are commonly used as anti-inflammatory drugs which have some side effects; finding the treatments for ALI safely and effectively is meaningful [8]. In recent years, the prevention and treatment of ALI with TCM formulas have increasingly indicated the advantages of using TCM theory. Of note, significant positive effects have been achieved using Traditional Chinese Medicine (TCM) in treating ALI. Huoxiang Zhengqi Oral Liquid (HZOL) is the adjuvant preventive treatment of pulmonary inflammation in China [9]. HZOL is typically used in treating gastrointestinal and pulmonary diseases as it confers a number of pharmacological benefits, including antibacterial, analgesic, and antiviral effects, along with regulation of gastrointestinal function [10]. It is often used in the clinical treatment of functional dyspepsia, diarrhea, acute gastroenteritis, gastrointestinal cold, and other diseases. It can be used not only to regulate gastrointestinal diseases including irritable bowel syndrome, vomiting, and diarrhea but also to treat influenza-like lung diseases resulting from wind chill or heat dampness, in line with the TCM theory [11].

Modern studies have revealed that the active ingredients of HZOL and its pharmacological experiments have demonstrated that these ingredients exhibit antiulcerative, antibacterial, anti-inflammatory, and antiviral effects [12–15]. HZOL is used clinically in China for over 1,000 years in treating gastrointestinal-type colds on account of its anti-inflammatory properties and positive effects on immunological regulation. The main manifestations of gastrointestinal-type cold refer to influenza with gastrointestinal symptoms, which are caused by wind, cold, heat, and dampness invading the muscle surface and digestive systems according to the theory of TCM. HZOL exerts anti-inflammatory effect and immune regulation in gastrointestinal diseases in previous studies [16, 17]. However, detailed mechanisms of HZOL as an agent in lung disease remain unclear. Researches have verified that the active ingredients of HZOL exert anti-inflammatory, antibacterial, and immunomodulatory effects, which are effective in the treatment of ALI. Therefore, the therapeutic effect and mechanism of HZOL in treating ALI were performed in this study.

**Figure 1:**
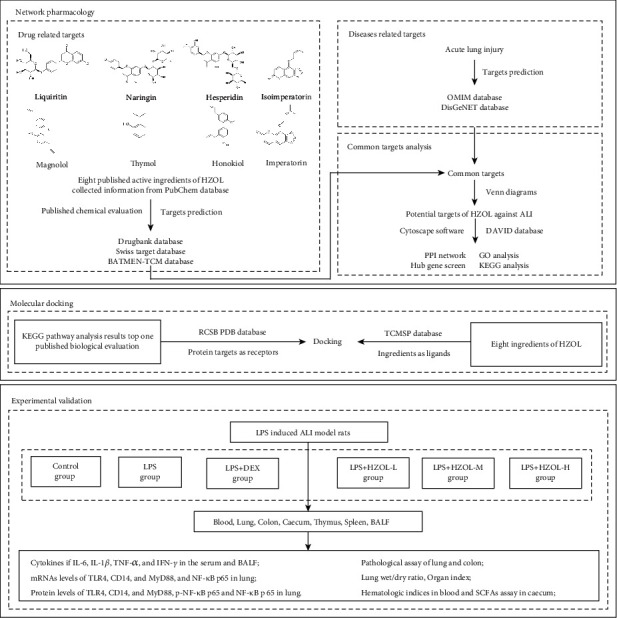
Procedures in this study.

At present, network pharmacology can be used to theoretically analyze multiple compounds and targets to predict the therapeutic mechanisms of action of TCMs in relation to treating diseases based on different databases [18]. An increasing number of studies on TCM formulations use network pharmacology to explore their pharmacodynamic mechanism [19, 20]. Furthermore, molecular docking was an auxiliary validation of network pharmacology. Therefore, we employed network pharmacology to identify putative targets and candidate pathways of HZOL against ALI. Then, molecular docking was employed to verify interaction between active components and pathway target proteins to further explore the molecular mechanism of network pharmacology prediction results. Finally, we validated the mechanism of HZOL in ALI model rats induced by LPS according to the predicted results and measured the content levels of SCFAs in the cecum. In summary, mechanisms of HZOL in treating ALI and gut injury were performed.  Figure 1 shows the study procedures.

## 2. Materials and Methods

### 2.1. Network Pharmacology and Molecular Docking

In accordance with the study conducted by [21], we performed a network pharmacological analysis of Huoxiang Zhengqi Oral Liquid (HZOL). Firstly, we collected information about the eight published active ingredients of HZOL—honokiol, magnolol, hesperidin, liquiritin, naringin, thymol, imperatorin, and isoimperatorin—from the PubChem database [12, 15]. Next, DrugBank, BATMEN-TCM, and Swiss Target databases were used to collect potential targets. Then, the OMIM and DisGeNET databases were used to research ALI targets using the keyword “Acute Lung Injury.” All of these targets were normalized in UniProt. Then, common targets were acquired using a tool to calculate and draw custom Venn diagrams. Next, a common target PPI network was constructed and the top five hub genes were obtained. GO and KEGG analyses were performed by the DAVID online database; then, “functional annotation clustering” was selected to export the data and screen the key pathway. Finally, molecular docking was done to predict the interaction of ingredient ligands and target receptors. The crystal structures of targets in the predicted key pathway were downloaded from the RCSB PDB database, while the structures of eight published active ingredients of HZOL were obtained from the TCMSP database. Then, we used the AutoDock 4.2.6 software to remove ligands, structure water molecules, and add polar hydrogen atoms and charges to the protein crystal structures before docking. Finally, the Discovery Studio software was used to evaluate the interaction between the targets and the active ingredients.

### 2.2. Preparation of Drugs

HZOL was purchased from the Taiji Group Chongqing Fuling Pharmaceutical Co., Ltd. Production of HZOL following the Chinese Pharmacopeia 2015 (Approved number, Z50020409; Lot number, 20071956). Quality of HZOL was able to ascertain both stability and controllability. Introduction of HZOL was follows: firstly, prescription of HZOL consists of ten herbs including Atractylodis Rhizoma 80 g, Magnoliae Officinalis Cortex 80 g, Poria 120 g, Pinelliae Rhizoma 80 g, Citri Reticulatae Pericarpium 80 g, Angelicae Dahuricae Radix 120 g, Arecae Pericarpium 120 g, Licorice extract 10 g, Patchouli oil 0.8 mL, and volatile oil in Perillae Folium 0.4 mL. Secondly, HZOL was standardised prepared following the method of the criteria of the Chinese Pharmacopeia 2015 as previous study described [10]. Thirdly, content determination of HZOL was controlled with magnolol, honokiol, and hesperidin. In a nutshell, per milliliter of HZOL contained magnolol and honokiol no less than 0.30 mg and hesperidin no less than 0.10 mg.

### 2.3. Animals and Treatment

Healthy male Wistar rats weighing 180–200 g (12 weeks, permit number SCXK, Beijing, 2016-0002) were purchased from SPF (Beijing) Biotechnology Co., Ltd. (Beijing, China) and kept in standard cages with standard laboratory animal feed and water. The rats were housed at a constant temperature of 22 ± 2°C and a relative humidity of 55 ± 5%, with 12 h day/night cycles. The use of rats and the experimental protocols were reviewed and approved by the Institutional Animal Care and Use Committee at Beijing University of Chinese Medicine (No. BUCM-4-2020100907-4029).

A total of 60 rats were randomly divided into six groups (*n* = 10), and the used dose administration was as follows: a control group (control), lipopolysaccharide group (LPS), LPS+dexamethasone group (LPS+DEX), LPS+HZOL low-dose group (LPS+HZOL-L), LPS+HZOL middle-dose group (LPS+HZOL-M), and LPS+HZOL high-dose group (LPS+HZOL-H). The daily dose of HZOL for human adults is 0.33 mL/kg (clinical dose is 20 mL/one day/60 kg body weight of one person), and the equivalent dose in rat is 10-fold that of adults in previous reported research (3.33 mL/kg/d, 10-fold of the clinical dose) [10]. The rats except the control group were administered a single dose of LPS (5 mg/kg, Escherichia coli 055: B5 L2880, Sigma, USA) intraperitoneally according to a previous study [22]. The rats in the LPS+HZOL groups received an oral administration of the agents for 2 weeks before LPS injection at different doses (LPS+HZOL-L, 1.67 mL/kg, 5-fold of the clinical dose; LPS+HZOL-M, 3.33 mL/kg, 10-fold of the clinical dose; and LPS+HZOL-H, 6.67 mL/kg, 20-fold of the clinical dose) following a previous study [10], while DEX (5 mg/kg) was administrated via stomach perfusion for six days before the LPS treatments [18, 23]. The dosage was adjusted according to the body weight of rats. After 6 h LPS administration, the animals were anaesthetized with 20% urethane (5 mL/kg) by intraperitoneal injection and related biological samples were collected.

### 2.4. Pathological Assay and Pathology Scores

We obtained partial sections of lung tissues and colon tissues. We routinely fixed the tissue with 4% paraformaldehyde and then gradient dehydration. The samples were cut, embedded in paraffin, bleached, and stained in hematoxylin and eosin (H&E). The score of lung injury and colon injury was performed by other investigators who were blinded to the study. For the lung injury, scoring principles were as follows: (1) alveolar congestion, (2) hemorrhage, (3) infiltration of neutrophils in the airspace or vessel wall, and (4) thickness of the alveolar wall formation. Each lung injury item was scored as follows: no damage or minimal damage = 0, mild damage = 1, moderate damage = 2, severe damage = 3, and diffuse injury = 4. Then, the lung injury was scored according to previous study and all scores were added up to obtain a total score [23, 24]. Besides, the total histological score for colon injury ranges from 0 to 8 according to previous study as follows: epithelium: normal morphology = 0, loss of goblet cells = 1, loss of goblet cells in large areas = 2, loss of crypts = 3, and loss of crypts in large areas = 4; infiltration: no infiltrate = 0, infiltrate around crypts = 1, infiltrate into the lamina muscularis mucosae = 2, extensive infiltration into the lamina muscularis mucosae and thickening of the mucosa = 3, and infiltration of the submucosal layer = 4 [25].

### 2.5. Lung Wet/Dry Ratio and Organ Index

In accordance with the reported study, we calculated lung wet/dry ratio = wet weight/dry weight [23]. In addition, the whole body and absolute thymus and spleen weights were recorded, and the organ index was calculated as organ index = absolute organ weight/body weight.

### 2.6. Hematologic Index Assay

Using a Mindray automatic animal blood cell analyzer (BC-2800 VET, Shenzhen, China), part of the whole blood was collected in an EDTA-treated blood collection tube for the determination of white blood cell (WBC) count, granulocyte concentration (GRAN%), basophil concentration (BASO%), eosinophil concentration (EOS%), lymphocyte concentration (LYM%), and blood platelet count (PLT).

### 2.7. Enzyme-Linked Immunosorbent Assay (ELISA)

The serum samples were obtained through separation from anterior blood samples. Besides, the bronchoalveolar lavage fluid (BALF) was collected from the left lung by bronchial intubation. Normal saline with a total volume of 3 mL was instilled and retrieved three times. Finally, the cytokines interleukin-6 (IL-6), interleukin-1*β* (IL-1*β*), tumor necrosis factor-*α* (TNF-*α*), and interferon-*γ* (IFN-*γ*) in the serum and BALF samples were measured using commercially available ELISA kits (Ray Biotech. Inc., USA; ProteinTech®, Chicago, IL, USA).

### 2.8. Real-Time Quantitative Polymerase Chain Reaction (RT-qPCR) Analysis

Levels of Toll-like receptor 4 (TLR4), cluster of differentiation 14 (CD14), myeloid differentiation factor 88 (MyD88), and nuclear factor-kappa B (NF-*κ*B p65) mRNAs were identified by RT-qPCR. Lung tissues were dissociated with TRIzol reagent, and total RNA was determined. Then, RNA of each sample was utilized to synthesize the first-strand cDNA.  Table 1 shows the sequences for primers. Next, the cDNA was amplified. 2^-∆∆Ct^ method was used for quantification.

### 2.9. Western Blot Analysis

The concentration of total proteins in lung tissues was tested using a BCA kit. Next, 40 *μ*g of protein samples was dissolved in an equal volume of loading buffer and was then separated by 10% SDS-PAGE and transferred onto PVDF membranes. After blocking with TBST containing 5% nonfat milk for 1 h at room temperature, the membranes were incubated with primary antibodies at 4°C overnight. The primary antibodies used were TLR4, CD14, MyD88, p-NF-*κ*B p65, NF-*κ*B p65, and GAPDH, respectively (Proteintech Group, Rosemont, IL, USA; Cell Signaling Technology, Beverly, MA, USA). Next, the membranes were washed in TBST and incubated with secondary antibodies for 1.5 h at room temperature. Finally, the signals of the target proteins were detected, and target bands were exposed by chemiluminescence and quantified using ImageJ software.

### 2.10. Measurement of SCFAs in Cecum Contents

SCFAs were tested using GC-MS analysis according to the protocol used in a previous study [6]. The conditions of Thermo TRACE 1310-ISQ LT were coupled with a GC column of Agilent HP-INNOWAX and the MS conditions of electron impact ionization (EI) source, single ion mode (SIM) scanning mode, and 70 eV electron energy.

### 2.11. Statistical Analysis

All experimental data were expressed as the means ± SD. Statistical analysis was carried out using SPSS 20.0 software in a one-way analysis of variance (ANOVA) followed by the least significant difference (LSD) post hoc test for comparison of multiple groups. *P* value < 0.05 was considered statistically significant.

## 3. Results

### 3.1. Common Targets, Hub Genes, GO, and KEGG Analysis of HZOL in Treating ALI

**Figure 2:**
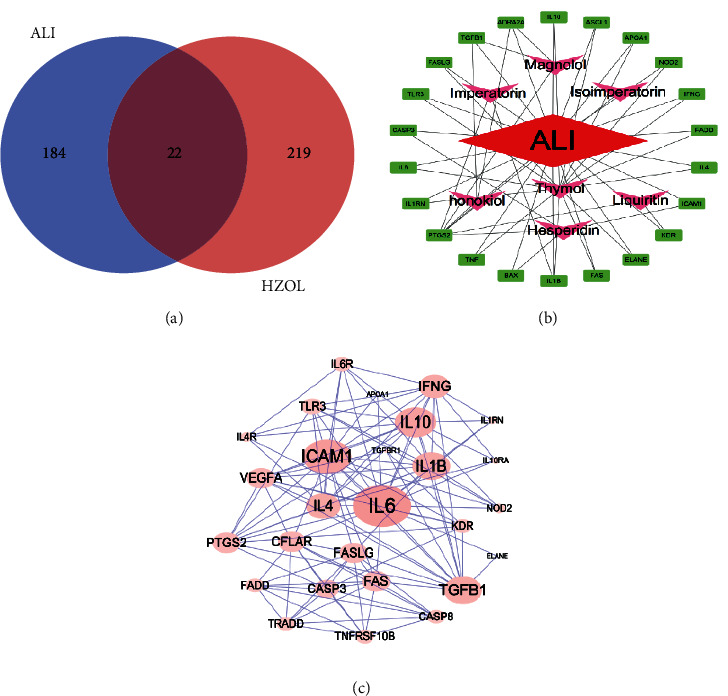
Common target network analysis and identification of hub genes of HZOL for ALI. (a) Common targets of HZOL and ALI. (b) Common target-active ingredient-disease network. (c) PPI network of twenty-two common targets with 22 nodes and 214 edges.

**Figure 3:**
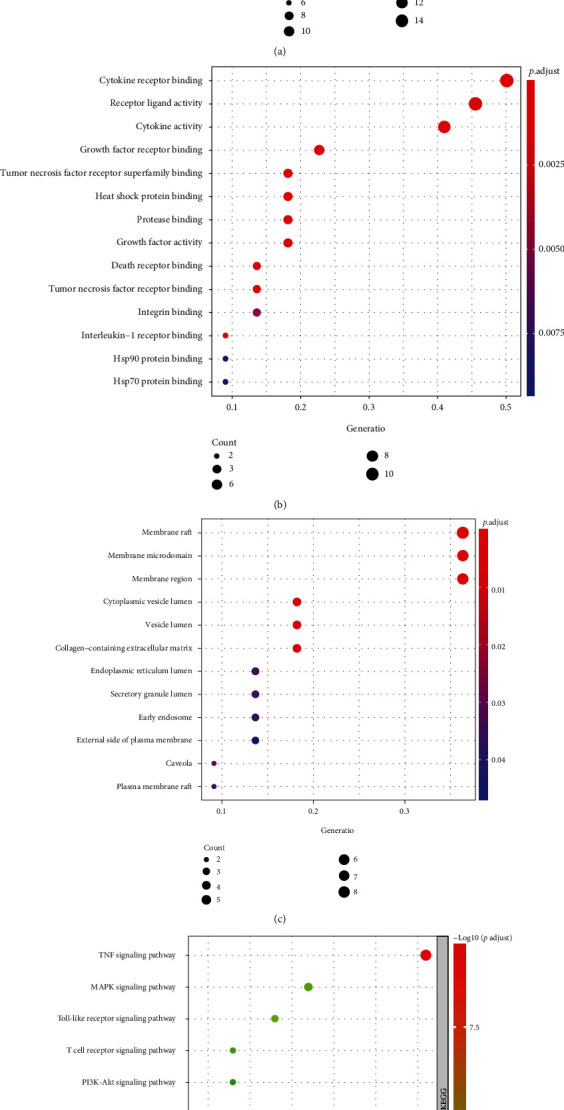
GO and KEGG analyses of twenty-two common target-related genes. (a) Top 20 biological processes (BPs). (b) Top 14 molecular functions (MFs). (c) Top 12 cellular components (CCs). (d) Top 10 KEGG signaling pathways.

Information of identified eight active ingredients of HZOL is shown in Table 2, while as shown in Figure 2(a), after normalizing in UniProt, 241 gene symbols of HZOL and 206 gene symbols of ALI were obtained, and 22 common targets were obtained after the process of taking the intersection. The active ingredient-common target-disease network is shown in Figure 2(b). In Figure 2(c), the results of a PPI network of twenty-two common targets showed that the top five hub genes were IL-6, IL-10, ICAM-1, IL-1*β*, and IL-4. As shown in Figures 3(a)–3(d), the fundamental mechanisms of HZOL on ALI were screened, including 1541 BPs, 12 CCs, 14 MFs, and 82 significant pathways (*P* value ≤ 0.05). Furthermore, the results shown in Figure 3 suggested that HZOL treating ALI mainly involved cell adhesion, immune response, inflammatory response, and signal transduction. In Figure 3(d), key signaling pathways were closely related to the TNF signaling pathway, MAPK signaling pathway, Toll-like receptor signaling pathway, NOD-like receptor signaling pathway, NF-kappa B signaling pathway, etc.

### 3.2. Potential Mechanisms of HZOL Treating ALI by Network Pharmacological Analysis

The functional annotation clustering results involved eight clusters. Information relating to the top 1 cluster, with an enrichment score of 5.51, is provided in Table 3. This finding indicated that the NF-*κ*B pathway (hsa04064) was the key mechanism. Functional annotation clustering analysis results have shown that the NF-*κ*B pathway (hsa04064) was the potential pathway of HZOL in treating ALI in the top 1 cluster. Combining with the top 10 key signaling pathways of KEGG enrichment results above by overlapping, the NF-*κ*B pathway was screened out as the potential mechanisms of HZOL in treating ALI. Network pharmacological results prove that the NF-*κ*B signaling pathway was the potential anti-inflammatory pathway of HZOL in treating ALI.

Furthermore, we explored the published efficacy evaluation of the eight active ingredients of HZOL regarding their mechanism of action in ALI/ARDS or inflammation. The published research results in Table 4 have shown that the 8 main active components of HZOL including honokiol, magnolol, hesperidin, liquiritin, naringin, thymol, imperatorin, and isoimperatorin performed an anti-inflammatory and immune regulation function, and their experimental results showed that the mechanisms were mainly revealed in inhibiting the NF-*κ*B pathway, MAPK pathway, and TLR4 receptor to restrain the inflammatory responses. Previous studies have reported that LPS can bind to a receptor complex consisting of TLR4 and CD14, and then, NF-*κ*B protein can be activated via a myeloid differentiation factor MyD88-dependent pathway, eventually leading to the production of NF-*κ*B-induced proinflammatory cytokines [26]. According to our network pharmacological prediction of the NF-*κ*B signaling pathway and the biological published effect mechanisms involving the NF-*κ*B signaling pathway and TLR4 receptor, targets in the TLR4/NF-*κ*B p65 pathway involving TLR4, CD14, MyD88, and NF-*κ*B p65 were selected as the key targets in our study to observe the treatment of HZOL in ALI. All the raw data of the network pharmacology analysis results are shown in Supplementary Material (1).

### 3.3. Molecular Docking Analysis between Active Ingredients and Core Proteins

**Figure 4:**
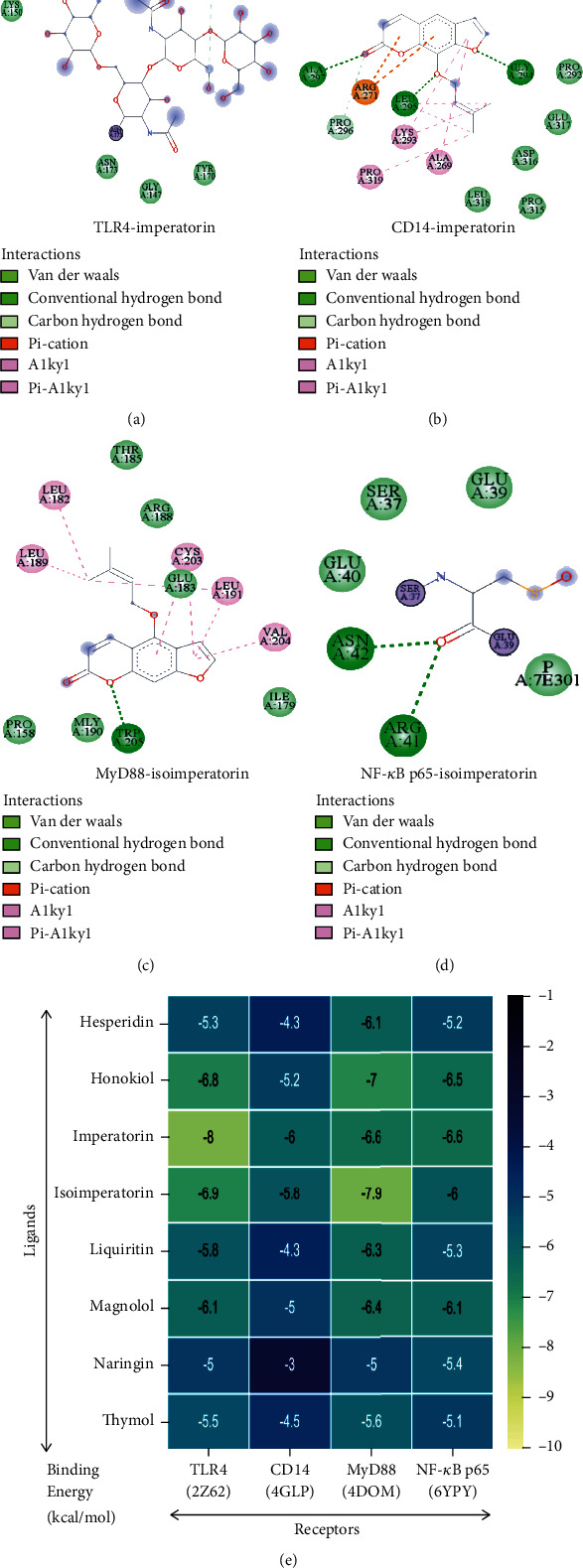
Molecular docking results of active ingredients and TLR4/NF-*κ*B p65 signaling pathway-related targets. (a) TLR4, with PDB ID 2Z62; (b) CD14, with PDB ID 4GLP; (c) MyD88, with PDB ID 4DOM; (d) NF-*κ*B p65, with PDB ID 6YPY; (e) docking results of binding energy between ligands and receptors.

It is known that the lower the binding energy, the stronger the binding pattern between the ligands and the receptors. Results showed that imperatorin bound well to TLR4, CD14, and NF-*κ*B p65 (Figures 4(a), 4(b), and 4(d)) with the lowest value of the binding energy, while isoimperatorin bound well to MyD88 (Figure 4(c)) with the minimum binding energy. Moreover, their binding patterns mainly involved hydrogen bonding and hydrophobic interactions. The molecular docking results of the binding energy between ligands and receptors are shown in Figure 4(e). These results suggested that eight compounds especially imperatorin and isoimperatorin could combine well with the targets in the NF-*κ*B inflammation pathway preliminary verifying the prediction results of network pharmacology.

### 3.4. HZOL Ameliorated Lung Edema and Lung Pathological Injury and Inhibited the Enhancement of Thymus and Spleen Indexes Induced by LPS

**Figure 5:**
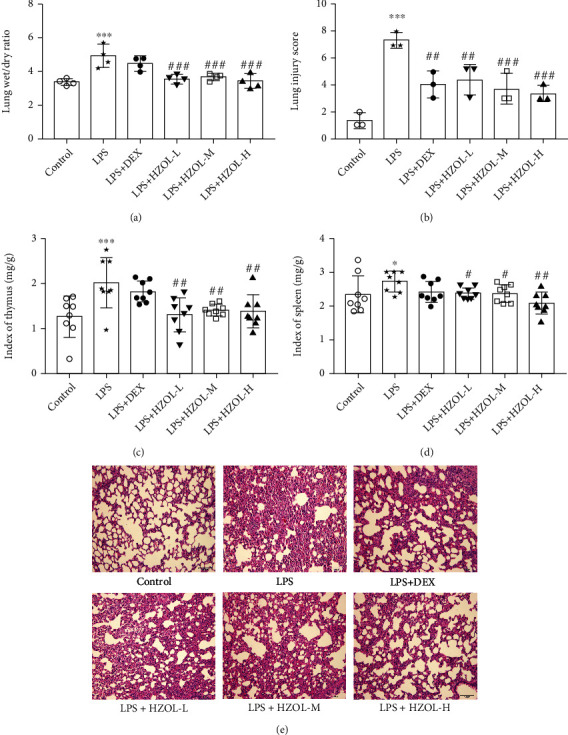
Lung wet/dry ratio, organ index, and pathohistological evaluations of lung tissues. (a) Lung wet/dry ratio (*n* = 4). (b) Lung injury score (*n* = 3). (c) Thymus index (*n* = 8). (d) Spleen index (*n* = 8). (e) H&E staining of lung tissues in control, lipopolysaccharide (LPS, 5 mg/kg), LPS+dexamethasone (DEX, 30 *μ*g/kg), LPS+HZOL-L (1.67 mL/kg), LPS+HZOL-M (3.33 mL/kg), and LPS+HZOL-H (6.67 mL/kg) groups (*n* = 3). Mean ± SD. ^∗^*P* < 0.05 and ^∗∗∗^*P* < 0.001 vs. the control group. ^#^*P* < 0.05, ^##^*P* < 0.01, and ^###^*P* < 0.001 vs. the LPS group.

As the results show in Figures 5(a) and 5(b), the wet/dry ratio and lung injury score were remarkably elevated after LPS stimulation of the lung. Conversely, they were distinctly attenuated by HZOL. In addition, the organ indexes of the thymus and spleen were calculated to assess the state of the host immune capability. In Figures 5(c) and 5(d), thymus and spleen indexes increased in model rats, while these results were reversed by treating with HZOL in different doses. However, the organ protection of the DEX group was inferior to HZOL in lung edema, and abnormal increases in the indexes of the thymus and spleen were observed—changes induced by LPS. Furthermore, in the LPS group in Figure 5(e), the alveolar structure was disordered and the alveolar wall was not complete. In contrast, the HZOL group and the DEX group showed that the continuity of alveolar structure and the incompleteness of the alveolar wall were improved, and the infiltration of inflammatory cells and interstitial cells was decreased with different doses of HZOL.

### 3.5. HZOL Alleviated Colonic Pathological Injury and Modulated Short-Chain Fatty Acids Induced by LPS

**Figure 6:**
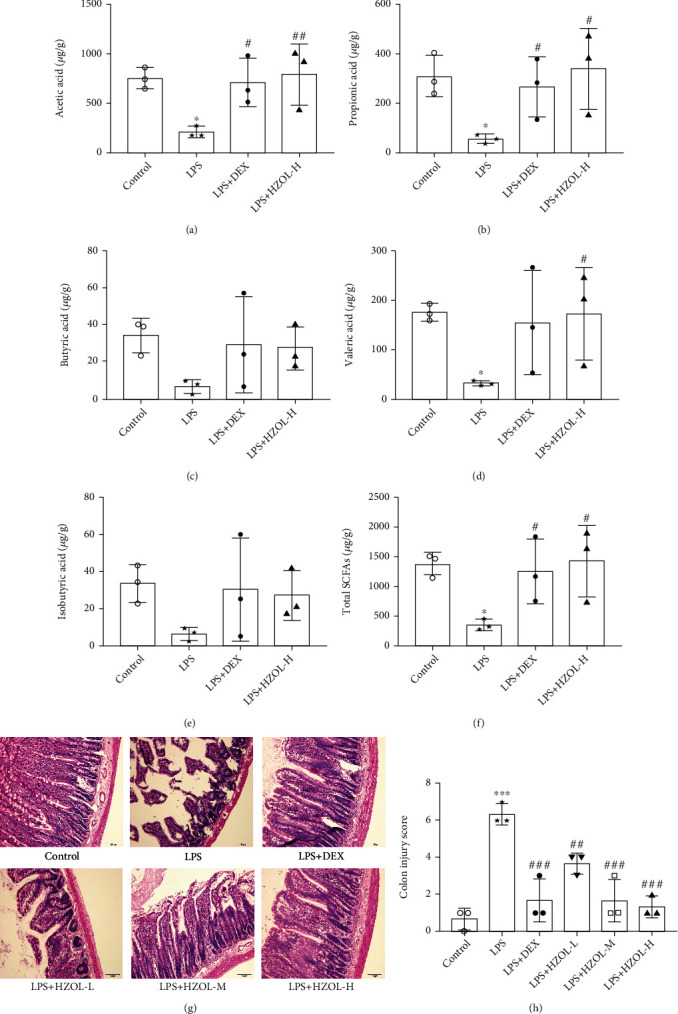
Analytical parameters for GC-MS method of SCFAs in cecum contents and pathohistological evaluation of colon tissues. (a) Acetic acids. (b) Propionic acids. (c) Butyric acids. (d) Valeric acids. (e) Isobutyric acids. (f) Total SCFAs. (g) H&E staining of colon tissues. (h) Colon injury score. Mean ± SD (*n* = 3). ^∗^*P* < 0.05 and ^∗∗∗^*P* < 0.001 vs. the control group. ^#^*P* < 0.05, ^##^*P* < 0.01, and ^###^*P* < 0.001 vs. the LPS group.

The results in Figures 6(a)–6(f) showed that total SCFAs and individual SCFAs (acetic, propionic, butyric, valeric, and isobutyric acids) in the cecum were reduced in LPS rats. After pretreatment with DEX and HZOL, total SCFAs and individual SCFAs in the cecum were significantly increased. In addition, pathohistological evaluations and colon injury score of colon tissues were performed, as shown in Figures 6(g) and 6(h). Colonic histopathology results revealed that in the control group, the colonic mucosa was intact and smooth, and the glandular layer was clear. However, in the LPS model group, the colonic mucosal epithelium was eroded, the crypt structure of the glands was destroyed, the goblet cells were lost, and inflammatory cells were infiltrated. After pretreatment with HZOL, the colonic mucosal damage was significantly alleviated, the crypts of glands were arranged in order, the epithelial structure was complete, and the inflammatory cell infiltration was reduced. Besides, HZOL attenuated the severity degree of colon injury score. These results demonstrated that HZOL has protective effects on LPS-induced gut injury.

### 3.6. HZOL Improving Hematologic Indices

**Figure 7:**
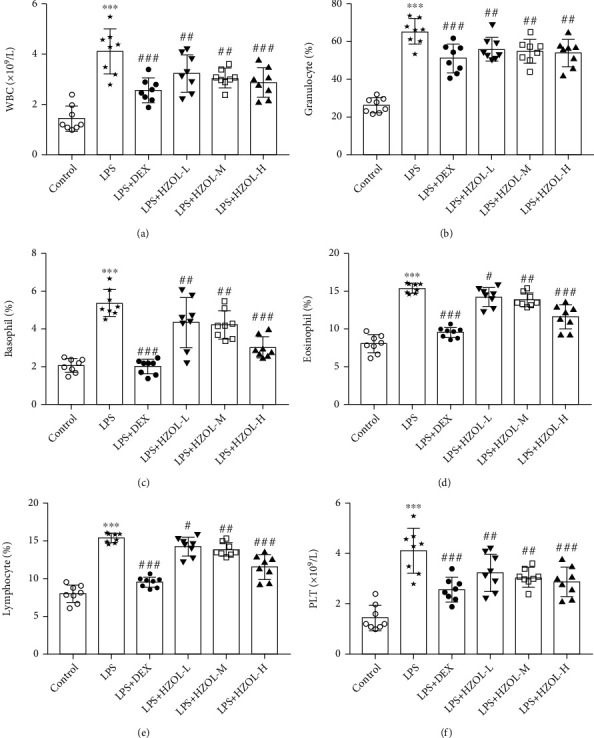
Hematologic indices of (a) white blood cells (WBC), (b) granulocyte percentage (GRAN%), (c) basophil percentage (BASO%), (d) eosinophil percentage (EOSIN%), (e) lymphocyte percentage (LYM%), and (f) blood platelets (PLT) on plasma of rats. Mean ± SD (*n* = 8). ^∗∗∗^*P* < 0.001 vs. the control group. ^#^*P* < 0.05, ^##^*P* < 0.01, and ^###^*P* < 0.001 vs. the LPS group.

We next explored the pathological changes of related organs and the physiological and pathological changes in the circulation system, and we assessed the alteration in blood cells from the rats' plasma. In our results in Figures 7(a)–7(f), the data showed that WBCs, GRAN%, BASO%, and EOSIN% were increased, while the PLT and LYM% were decreased in plasma—changes induced by LPS. Notably, DEX and HZOL in different doses reverted the hematologic indices markedly by reducing the WBC count, GRAN%, BASO%, and EOSIN% and increasing the PLT and LYM%.

### 3.7. HZOL Alleviated Inflammatory Responses Induced by LPS

**Figure 8:**
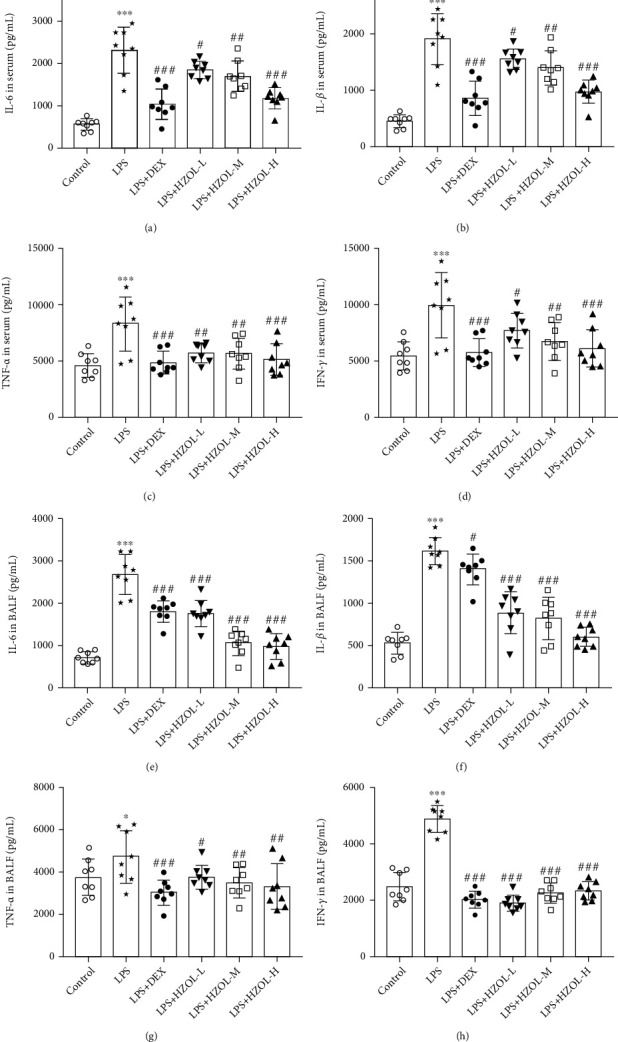
IL-6, IL-1*β*, TNF-*α*, and INF-*γ* concentrations in serum and BALF. The concentrations of (a) IL-6, (b) IL-1*β*, (c) TNF-*α*, and (d) INF-*γ* in serum and (e) IL-6, (f) IL-1*β*, (g) TNF-*α*, and (h) INF-*γ* in BALF were measured by ELISA kits. Mean ± SD (*n* = 8). ^∗^*P* < 0.05 and ^∗∗∗^*P* < 0.001 vs. the control group. ^#^*P* < 0.05, ^##^*P* < 0.01, and ^###^*P* < 0.001 vs. the LPS group.

The serum and bronchoalveolar lavage fluid concentrations of IL-6, IL-1*β*, TNF-*α*, and IFN-*γ* were evaluated to assess the anti-inflammatory activity of HZOL in this research. The results are shown in Figures 8(a)–8(h). LPS significantly increased the production of IL-6, IL-1*β*, TNF-*α*, and IFN-*γ*, while the HZOL and DEX groups inhibited these cytokines in the rats' serum and BALF.

### 3.8. Effects of HZOL Pretreatment on TLR4/NF-*κ*B p65 Signaling Pathway

**Figure 9:**
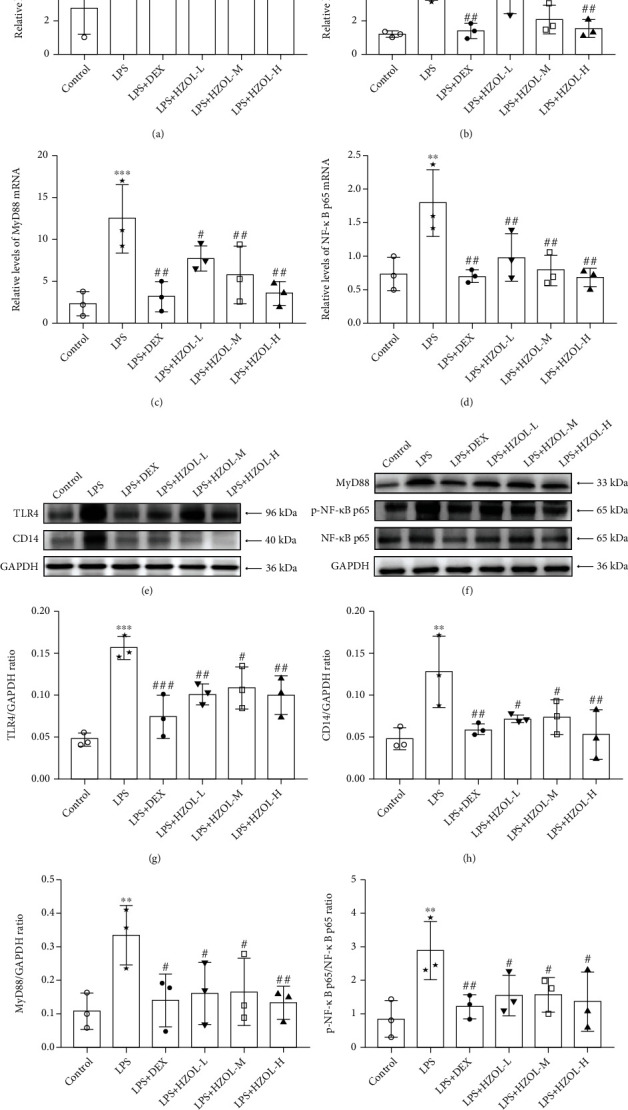
HZOL treatment effect on the expression of TLR4, CD14, MyD88, p-NF-*κ*B p65, and NF-*κ*B p65. Expression of mRNA of (a) TLR4, (b) CD14, (c) MyD88, and (d) NF-*κ*B p65 in lung tissue was detected by RT-qPCR, while the protein expression of (e, g) TLR4, (e, h) CD14, (f, i) MyD88, and (f, j) p-NF-*κ*B p65/NF-*κ*B p65 ratio in lung tissue was detected by western blotting. Mean ± SD (*n* = 3). ^∗∗^*P* < 0.01 and ^∗∗∗^*P* < 0.001 vs. the control group. ^#^*P* < 0.05, ^##^*P* < 0.01, and ^###^*P* < 0.001 vs. the LPS group.

**Figure 10:**
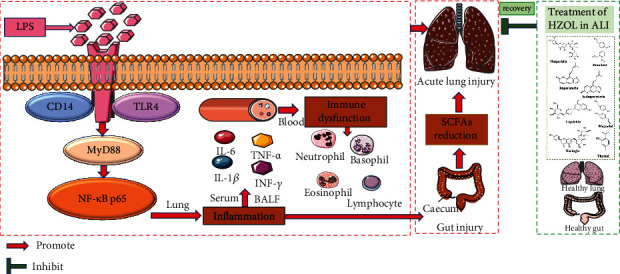
Schematic diagram of the TLR4/NF-*κ*B p65 signaling pathways related to the anti-inflammatory effects of HZOL on LPS-induced ALI.

The mRNA levels in the LPS group involving TLR4, CD14, MyD88, and NF-*κ*B p65 were significantly upregulated, respectively. In contrast, they were significantly downregulated by DEX and HZOL in different doses in Figures 9(a)–9(d). However, the results shown in Figures 9(f)–9(j) of our study indicated that HZOL only affected the protein expression level of p-NF-*κ*B p65, but not the expression level of total NF-*κ*B p65. Besides, in Figures 9(e)–9(j), upregulating protein levels of TLR4, CD14, MyD88, and p-NF-*κ*B p65/NF-*κ*B p65 ratio induced by LPS were significantly downregulated, respectively, after treating with HZOL or DEX. These observational findings demonstrated that expressions of TLR4, CD14, MyD88, and p-NF-*κ*B p65 on the TLR4/NF-*κ*B p65 signaling pathway in ALI mice were downregulated after pretreatment with HZOL. The mechanism is shown in Figure 10. All the raw data of the experimental validation results are shown in Supplementary Material (2).

## 4. Discussion

Lipopolysaccharides (LPS) can cause a bacterial type of pneumonia, which can eventually lead to the onset of ALI. ALI can be induced by different diseases and pathological states, such as trauma, pneumonia, sepsis, and endotoxemia [44]. Besides, studies demonstrated that ALI induced by LPS can release proinflammatory cytokines in the lung and lead to immune dysfunction, indicating that LPS can be used to establish animal models of ALI that exhibit similar clinical characteristics to ALI [45–47]. LPS-induced ALI is administered in three ways mainly. The first one to induce ALI directly is the intranasal perfusion or intratracheal instillation, but the course of disease rapidly develops from ALI to ARDS and causes respiratory failure and death finally [48]. The second one of ALI is induced by the caudal vein injection, which is suitable to study the early pathogenesis of ALI in a short time, but the operation is difficult and the success rate is relatively low [49]. The third one is intraperitoneal injection which is a common and easy method to induce the sepsis causing systemic inflammatory changes and then simulating secondary lung damage to establish ALI [50]. Moreover, colon barrier dysfunction, acute colon injury, and disturbance of microbiota appearance can be induced by LPS according to previous reported researches [51]. Therefore, anti-inflammatory on regulation of lung and colon injury is an effective strategy for the treatment of ALI rats induced by LPS. In our study, intraperitoneal injection method was used to induce ALI and we observed the inflammatory changes in the lung as well as in the gut as results.

Microbiome metabolite SCFAs can promote antimicrobial and anti-inflammatory pathways, participating in inflammation response in the lung and gut [52]. SCFAs are the important contributors in the modulation of gut-lung dysfunction. In a previous study of a mouse model either in pneumonia exposure high-dose propionate or in lung ischemia reperfusion injury, once lung inflammation occurred, SCFAs were produced in the gut which can enter the lungs through blood circulation, then activating the lung immunity, increasing the content of SCFAs in the lung, inhibiting the production of proinflammatory factors, and thus modulating lung immune responses to inhibit inflammation finally [53]. Moreover, once inflammation is triggered by LPS, the number of WBCs in rats increases significantly to help the host resist bacterial invasion. Besides, the recruitment of neutrophils, eosinophils, and basophils is essential to the host for killing bacteria; the GRAN%, EOSIN%, and BASO% contributed in regulating the inflammatory response [54]. In addition, the LYM% may reflect the immune status, and blood platelets enhance the stress elasticity of the host and accelerate the reconstruction rate of injured pulmonary vessels [55]. LPS may also cause extensive damage to organs, including the immune-related thymus and spleen, leading to immune dysfunction [22]. Consistently, the results of our in vivo experiments demonstrated that pretreatment with HZOL could alleviate pulmonary edema, downregulating the lung wet/dry ratio, improving the lung pathological assay, inhibiting LPS-induced abnormal enhancement of the immune-related thymus and spleen, and maintaining the balance in blood parameters by reducing the WBC, GRAN%, BASO%, and EOSIN%, while increasing the PLT and LYM%. It appears that HZOL can also repair colon damage and regulate immune disorders by increasing the levels of all SCFAs and individual SCFAs (acetic, propionic, butyric, valeric, and isobutyric acids) in the cecum during the stress of LPS-induced ALI. Our results suggested that HZOL exerts anti-inflammatory effects on LPS-induced ALI.

In the mechanism prediction of action relating to the therapeutic effect of HZOL in ALI, network pharmacology results suggested that the NF-*κ*B pathway was the principal pathway. Besides, published literature demonstrated that the NF-*κ*B pathway and TLR4 receptor were closely linked to cytokine production and crucial in regulating inflammatory responses, which are also related to the development of ALI [23]. Previous studies have reported that LPS can bind to a receptor complex consisting of TLR4 and CD14, and then, NF-*κ*B protein can be activated via a myeloid differentiation factor MyD88-dependent pathway, eventually leading to the production of NF-*κ*B-induced proinflammatory cytokines [26]. In addition, molecular docking results suggested that eight ingredients of HZOL especially imperatorin and isoimperatorin combine well with the targets in the NF-*κ*B inflammation pathway of TLR4, CD14, MyD88, and NF-*κ*B p65, preliminary verifying the prediction results of network pharmacology. Furthermore, the inflammatory response of the host is caused by IL-6, IL-1*β*, TNF-*α*, and IFN-*γ*, along with constant stimulation of excessive LPS, triggering an inflammatory storm that ultimately leads to the onset of ALI [56, 57]. According to our network pharmacological prediction results and related ALI pharmacological literature reports, we explored the effect of HZOL on the TLR4/NF-*κ*B p65 pathway in an ALI mouse model. Through a pharmacological experiment involving an LPS-induced ALI rat model, our results suggested that uncontrolled prolongation of inflammatory responses was remarkably inhibited by HZOL through downregulation IL-6, IL-1*β*, TNF-*α*, and IFN-*γ* in serum and the bronchoalveolar lavage fluid. Moreover, HZOL downregulated the protein expression and the mRNA levels of TLR4, CD14, MyD88, p-NF-*κ*B p65, and NF-*κ*B p65 in ALI model rats. Our theoretical prediction and in vivo experimental verification are consistent with those of previous animal studies, indicating that the TLR4/NF-*κ*B pathway is closely related to LPS-induced reactions [58–60]. Results indicated that the protective effect of HZOL against ALI was mainly involved with the TLR4/NF-*κ*B p65 inflammatory pathway.

HZOL increased the levels of SCFAs, inhibiting the accumulation of inflammatory cytokines and attenuating the expressions of TLR4, CD14, MyD88, p-NF-*κ*B p65, and NF-*κ*B p65 of the TLR4/NF-*κ*B p65 inflammatory pathway in our LPS-induced lung and gut injury model. Briefly, pretreatment with HZOL contributes to protective effect against LPS-induced ALI and gut injury. Mechanisms involved may lead to increased immune function and reduced inflammatory responses by increasing SCFA levels and inhibiting activation of the TLR4/NF-*κ*B p65 pathway. Although HZOL has been used for many centuries in humans, little is known about its pharmacological role in lung disease. Our study explored the therapeutic effect and mechanism of its antipneumonia effect. Finally, our study contributed to the pharmacology of HZOL in relation to its application in the treatment of ALI. This study provided experimental evidence regarding the role of HZOL in preventing and treating acute lung injury and gut injury. Future research should focus on the lung-gut axis to demonstrate the anti-inflammatory of the lung-protective qualities of HZOL in order to encourage a broader application of HZOL in clinical settings.

## 5. Conclusions

In summary, HZOL plays an important anti-inflammatory and immune regulation role in the lung-gut axis by balancing the levels of SCFAs in the cecum, reducing the accumulation of inflammatory cytokines, downregulating inflammatory signaling pathways, relieving pulmonary edema, and repairing damage to colon tissue in the LPS-induced ALI rat model. HZOL can reverse LPS-induced changes relating to the inflammatory cytokines and immune dysfunction and attenuating the expressions of TLR4, CD14, MyD88, NF-*κ*B p65, and p-NF-*κ*B p65 of the TLR4/NF-*κ*B p65 pathway. Our study provided evidences for the practical application of HZOL in the treatment of ALI to encourage a broader application of HZOL in clinical settings. Furthermore, our study further suggests that the therapeutic effects and mechanisms of HZOL in preventing and treating ALI and gut injury may also have other clinical applications.

## Figures and Tables

**Table 1:** Primers for RT-qPCR.

Primer name	Sequence (5′ to 3′)
TLR4	
Forward	CCAGGTGTGAAATTGAGACAATTG
Reverse	AAGCTGTCCAATATGGAAACCC
CD14	
Forward	CGGGTTCCTACTCAGATTCTATTCG
Reverse	CGAGCCAGGTATCCGTTGTT
MyD88	
Forward	AAGGTGTCGTCGCATGGTG
Reverse	TTGGTGCAAGGGTTGGTATAGT
NF-*κ*B p65	
Forward	ACCTGTTCCAAAGAGCACCCA
Reverse	GGTCTGTGAACACTCCTGGGTC
GAPDH	
Forward	CTGGAGAAACCTGCCAAGTATG
Reverse	GGTGGAAGAATGGGAGTTGCT

**Table 2:** Information of the 8 active ingredients of HZOL.

Compound	Molecular formula	PubChem CID	Molecule ID	OB (%)	DL
Honokiol	C_18_H_18_O_2_	72303	MOL005955	60.67	0.15
Magnolol	C_18_H_18_O_2_	72300	MOL000210	69.19	0.15
Hesperidin	C_28_H_34_O_15_	10621	MOL007930	13.33	0.67
Liquiritin	C_21_H_22_O_9_	503737	MOL004903	65.69	0.74
Naringin	C_27_H_32_O_14_	442428	MOL005812	6.92	0.78
Thymol	C_10_H_14_O	6989	MOL002042	41.47	0.03
Imperatorin	C_16_H_14_O_4_	10212	MOL001941	34.55	0.22
Isoimperatorin	C_16_H_14_O_4_	68081	MOL001942	45.46	0.23

**Table 3:** Detailed information of top 1 cluster of functional annotation clustering results.

Annotation cluster 1, enrichment score: 5.5093				
Category	Term	*P* value	Genes	Fold enrichment
GOTERM_BP_DIRECT	GO:0045429: positive regulation of nitric oxide biosynthetic process	1.71*E*-09	IL6, IFNG, IL1B, PTGS2, TNF, ICAM1	106.5032
GOTERM_BP_DIRECT	GO:0042346: positive regulation of NF-kappa B import into the nucleus	2.21*E*-06	IL1B, PTGS2, TNF, TLR3	145.3853
GOTERM_BP_DIRECT	GO:0031622: positive regulation of fever generation	1.49*E*-05	IL1B, PTGS2, TNF	457.9636
KEGG_PATHWAY	hsa04064: NF-kappa B signaling pathway	0.001636	IL1B, PTGS2, TNF, ICAM1	15.81379

**Table 4:** Efficacy evaluation of the 8 active ingredients of HZOL for ALI/ARDS or inflammatory.

Compound	Therapeutic effects	Mechanisms	References
Honokiol	Honokiol inhibited lung inflammatory injury in ARDS mice induced by LPS	Inhibited Sirt3/AMPK pathway	[27]
	Honokiol inhibited inflammatory response in human monocyte-derived dendritic cells	Inhibited NF-*κ*B and MAPK pathways	[28]
	Honokiol has anti-inflammatory bioactivities in ALI mice induced by LPS	Inhibited NF-*κ*B pathway	[29]
Magnolol	Magnolol reducing inflammatory response and oxidative stress in sepsis mice	Inhibited HMGB1/TLR4/NF-*κ*B pathway	[30]
	Magnolol inhibits LPS-induced inflammatory response in RAW264.7 cells	Inhibited NF-*κ*B and MAPK pathways	[31]
Hesperidin	Hesperidin has anti-inflammatory effect in a systematic review and meta-analysis of randomized controlled clinical trials	Moderated VCAM-1 levels	[32]
	Hesperidin has anti-inflammatory effect in ALI rat induced by A virus (H1N1)	Inhibited MAPK pathways	[33]
Liquiritin	Liquiritin has anti-inflammatory effect in ALI mice induced by LPS	Inhibited NF-*κ*B pathway, TRPV1, and TRPA1	[34]
Naringin	Naringin anti-inflammation in peritoneal macrophage cells and sepsis mice	Inhibited NF-*κ*B activation	[35]
	Naringin has anti-inflammatory effect in ALI mice induced by LPS	Inhibited NF-*κ*B pathway	[36]
Thymol	Thymol anti-inflammatory effects in LPS-induced RAW264.7 cells	NF-*κ*B and MAPK pathways	[37]
	Thymol has anti-inflammatory effects in porcine intestinal epithelial cells	Reduce ROS and proinflammatory cytokine expression	[38]
Imperatorin	Imperatorin has antibacterial, anti-inflammatory, and antioxidant effects in LPS-induced macrophage-like RAW264.7 cells	Binds to TLR4 coreceptor and activates the Nrf2 pathway	[39]
	Imperatorin antibacterial and anti-inflammatory in RAW 264.7 macrophages	Inhibited NF-*κ*B and MAPK activation	[40]
	Imperatorin has anti-inflammatory effect in ALI mice induced by LPS	Inhibited the p-I*κ*B, JNK, ERK, and p38/MAPK	[41]
Isoimperatorin	Isoimperatorin as a potential TLR4 antagonist inhibited inflammatory responses induced by LPS in a mouse model	Inhibited LPS-TLR4/MD-2-NF-kappa B pathway	[42]
	Isoimperatorin has anti-influenza and anti-inflammatory effects in in vitro experiments	Inhibited NA-mediated progeny virus release	[43]

## Data Availability

The raw data supporting the conclusions of this article are included in the paper.

## Supplementary Materials

Supplementary Materials Supplementary Material (1): all the data of network pharmacology analysis. Supplementary Material (2): all the data of experimental validation results.
