# Supplementary material for: Huoxiang Zhengqi Oral Liquid Attenuates LPS-Induced Acute Lung Injury by Modulating Short-Chain Fatty Acid Levels and TLR4/NF-κB p65 Pathway
Source: Biomed Res Int. 2023 Feb 17;2023:6183551. doi: 10.1155/2023/6183551 (PMC9957650; doi:10.1155/2023/6183551)
Supplement: Supplementary Materials — Supplementary Material (1): all the data of network pharmacology analysis. Supplementary Material (2): all the data of experimental validation results. [file 6183551.f1.zip › Supplementary Material (1).pdf]

Supplementary Material (1): all the data of network pharmacology analysis.

Table S1: Huoxiang Zhengqi Oral Liquid (HZOL) related targets.

Compound name	Molecule ID	Uniprot Entry	Protein names	Gene symbol
honokiol	MOL005955	P34972	Cannabinoid receptor 2	CNR2
honokiol	MOL005955	P21554	Cannabinoid receptor 1	CNR1
honokiol	MOL005955	Q12791	Calcium-activated potassium channel subunit alpha-1	KCNMA1
honokiol	MOL005955	Q16558	Calcium-activated potassium channel subunit beta-1	KCNMB1
honokiol	MOL005955	Q9Y691	Calcium-activated potassium channel subunit beta-2	KCNMB2
honokiol	MOL005955	Q86W47	Calcium-activated potassium channel subunit beta-4	KCNMB4
honokiol	MOL005955	Q2I0M4	Leucine-rich repeat-containing protein 26	LRRC26
honokiol	MOL005955	P09917	Polyunsaturated fatty acid 5-lipoxygenase	ALOX5
honokiol	MOL005955	P19793	Retinoic acid receptor RXR-alpha	RXRA
honokiol	MOL005955	Q9GZV3	High affinity choline transporter 1	SLC5A7
honokiol	MOL005955	P31644	Gamma-aminobutyric acid receptor subunit alpha-5	GABRA5
honokiol	MOL005955	P47870	Gamma-aminobutyric acid receptor subunit beta-2	GABRB2
honokiol	MOL005955	P10253	Lysosomal alpha-glucosidase	GAA
honokiol	MOL005955	P14867	Gamma-aminobutyric acid receptor subunit alpha-1	GABRA1
honokiol	MOL005955	P47869	Gamma-aminobutyric acid receptor subunit alpha-2	GABRA2
honokiol	MOL005955	O95180	Voltage-dependent T-type calcium channel subunit alpha-1H	CACNA1H
honokiol	MOL005955	P55789	FAD-linked sulfhydryl oxidase ALR	GFER
honokiol	MOL005955	Q9NPC2	Potassium channel subfamily K member 9	KCNK9
honokiol	MOL005955	P23219	Prostaglandin G/H synthase 1	PTGS1
honokiol	MOL005955	P20309	Muscarinic acetylcholine receptor M3	CHRM3
honokiol	MOL005955	P11229	Muscarinic acetylcholine receptor M1	CHRM1
honokiol	MOL005955	P03372	Estrogen receptor	ESR1
honokiol	MOL005955	P10275	Androgen receptor	AR

honokiol	MOL005955	P35354	Prostaglandin G/H synthase 2	PTGS2
honokiol	MOL005955	P08173	Muscarinic acetylcholine receptor M4	CHRM4
honokiol	MOL005955	P19793	Retinoic acid receptor RXR-alpha	RXRA
honokiol	MOL005955	P23975	Sodium-dependent noradrenaline transporter	SLC6A2
honokiol	MOL005955	P35348	Alpha-1A adrenergic receptor	ADRA1A
honokiol	MOL005955	Q01959	Sodium-dependent dopamine transporter	SLC6A3
honokiol	MOL005955	P07550	Beta-2 adrenergic receptor	ADRB2
honokiol	MOL005955	P31645	Sodium-dependent serotonin transporter	SLC6A4
honokiol	MOL005955	Q16539	Mitogen-activated protein kinase 14	MAPK14
honokiol	MOL005955	P49841	Glycogen synthase kinase-3 beta	GSK3B
honokiol	MOL005955	P09960	Leukotriene A-4 hydrolase	LTA4H
honokiol	MOL005955	P27338	Amine oxidase [flavin-containing] B	MAOB
honokiol	MOL005955	O14757	Serine/threonine-protein kinase Chk1	CHEK1
honokiol	MOL005955	P07477	Trypsin-1	PRSS1
honokiol	MOL005955	P20248	Cyclin-A2	CCNA2
honokiol	MOL005955	Q15596	Nuclear receptor coactivator 2	NCOA2
honokiol	MOL005955	P61925	cAMP-dependent protein kinase inhibitor alpha	PKIA
honokiol	MOL005955	Q15788	Nuclear receptor coactivator 1	NCOA1
honokiol	MOL005955	O14827	Ras-specific guanine nucleotide-releasing factor 2	RASGRF 2
honokiol	MOL005955	P35348	Alpha-1A adrenergic receptor	ADRA1A
honokiol	MOL005955	P25100	Alpha-1D adrenergic receptor	ADRA1D
honokiol	MOL005955	P51693	Amyloid-like protein 1	APLP1
honokiol	MOL005955	O15405	TOX high mobility group box family member 3	TOX3
honokiol	MOL005955	P49840	Glycogen synthase kinase-3 alpha	GSK3A
honokiol	MOL005955	Q8NER 1	Transient receptor potential cation channel subfamily V member 1	TRPV1
honokiol	MOL005955	P08588	Beta-1 adrenergic receptor	ADRB1
honokiol	MOL005955	Q86YN 6	Peroxisome proliferator-activated receptor gamma coactivator 1-beta	PPARGC 1B
honokiol	MOL005955	P21918	D	DRD5
honokiol	MOL005955	P30542	Adenosine receptor A1	ADORA1
honokiol	MOL005955	Q7Z2W 7	Transient receptor potential cation channel subfamily M member 8	TRPM8
honokiol	MOL005955	O15164	Transcription intermediary factor 1-alpha	TRIM24
honokiol	MOL005955	P62508	Estrogen-related receptor gamma	ESRRG

honokiol	MOL005955	P35368	Alpha-1B adrenergic receptor	ADRA1B
honokiol	MOL005955	P36544	Neuronal acetylcholine receptor subunit alpha-7	CHRNA7
honokiol	MOL005955	Q8IVL1	Neuron navigator 2	NAV2
honokiol	MOL005955	Q99801	Homeobox protein Nkx-3.1	NKX3-1
honokiol	MOL005955	Q15465	Sonic hedgehog protein	SHH
honokiol	MOL005955	P09936	Ubiquitin carboxyl-terminal hydrolase isozyme L1	UCHL1
honokiol	MOL005955	P56705	Protein Wnt-4	WNT4
honokiol	MOL005955	P23634	Plasma membrane calcium-transporting ATPase 4	ATP2B4
honokiol	MOL005955	P78536	Disintegrin and metalloproteinase domain-containing protein 17	ADAM17
honokiol	MOL005955	P48436	Transcription factor SOX-9	SOX9
honokiol	MOL005955	O75762	Transient receptor potential cation channel subfamily A member 1	TRPA1
honokiol	MOL005955	Q92731	Estrogen receptor beta	ESR2
honokiol	MOL005955	P18825	Alpha-2C adrenergic receptor	ADRA2C
honokiol	MOL005955	P01308	Insulin [Cleaved into: Insulin B chain; Insulin A chain]	INS
honokiol	MOL005955	P01137	Transforming growth factor beta-1 proprotein [Cleaved into: Latency-associated peptide	TGFB1
honokiol	MOL005955	P41208	Centrin-2	CETN2
honokiol	MOL005955	Q9UJU2	Lymphoid enhancer-binding factor 1	LEF1
honokiol	MOL005955	Q08499	cAMP-specific 3',5'-cyclic phosphodiesterase 4D	PDE4D
honokiol	MOL005955	P49407	Beta-arrestin-1	ARRB1
honokiol	MOL005955	Q96B67	Arrestin domain-containing protein 3	ARRDC3
honokiol	MOL005955	P13945	Beta-3 adrenergic receptor	ADRB3
honokiol	MOL005955	P11473	Vitamin D3 receptor	VDR
honokiol	MOL005955	P08913	Alpha-2A adrenergic receptor	ADRA2A
honokiol	MOL005955	P18089	Alpha-2B adrenergic receptor	ADRA2B
honokiol	MOL005955	P01258	Calcitonin [Cleaved into: Calcitonin; Katacalcin	CALCA
honokiol	MOL005955	P07550	Beta-2 adrenergic receptor	ADRB2
honokiol	MOL005955	P51843	Nuclear receptor subfamily 0 group B member 1	NR0B1
honokiol	MOL005955	P21802	Fibroblast growth factor receptor 2	FGFR2
honokiol	MOL005955	Q9Y6Q9	Nuclear receptor coactivator 3	NCOA3

honokiol	MOL005955	Q99527	G-protein coupled estrogen receptor 1	GPER1
honokiol	MOL005955	P29475	Nitric oxide synthase, brain	NOS1
honokiol	MOL005955	P09493	Tropomyosin alpha-1 chain	TPM1
honokiol	MOL005955	P03372	Estrogen receptor	ESR1
honokiol	MOL005955	P15735	Phosphorylase b kinase gamma catalytic chain, liver/testis isoform	PHKG2
honokiol	MOL005955	Q92754	Transcription factor AP-2 gamma	TFAP2C
honokiol	MOL005955	Q15648	Mediator of RNA polymerase II transcription subunit 1	MED1
honokiol	MOL005955	Q12798	Centrin-1	CETN1
honokiol	MOL005955	P32121	Beta-arrestin-2	ARRB2
honokiol	MOL005955	P06401	Progesterone receptor	PGR
honokiol	MOL005955	P15514	Amphiregulin	AREG
honokiol	MOL005955	P41221	Protein Wnt-5a	WNT5A
honokiol	MOL005955	P41220	Regulator of G-protein signaling 2	RGS2
honokiol	MOL005955	P20800	Endothelin-2	EDN2
honokiol	MOL005955	P09917	Arachidonate 5-lipoxygenase	ALOX5
honokiol	MOL005955	P21554	Cannabinoid receptor 1	CNR1
honokiol	MOL005955	P34972	Cannabinoid receptor 2	CNR2
Magnolol	MOL000210	P23219	Prostaglandin G/H synthase 1	PTGS1
Magnolol	MOL000210	P20309	Muscarinic acetylcholine receptor M3	CHRM3
Magnolol	MOL000210	P11229	Muscarinic acetylcholine receptor M1	CHRM1
Magnolol	MOL000210	P03372	Estrogen receptor	ESR1
Magnolol	MOL000210	P10275	Androgen receptor	AR
Magnolol	MOL000210	Q14524	Sodium channel protein type 5 subunit alpha	SCN5A
Magnolol	MOL000210	P35354	Prostaglandin G/H synthase 2	PTGS2
Magnolol	MOL000210	Q14432	CGMP-inhibited 3',5'-cyclic phosphodiesterase A	PDE3A
Magnolol	MOL000210	P23975	Sodium-dependent noradrenaline transporter	SLC6A2
Magnolol	MOL000210	P35348	Alpha-1A adrenergic receptor	ADRA1A
Magnolol	MOL000210	Q01959	Sodium-dependent dopamine transporter	SLC6A3
Magnolol	MOL000210	P07550	Beta-2 adrenergic receptor	ADRB2
Magnolol	MOL000210	P31645	Sodium-dependent serotonin transporter	SLC6A4
Magnolol	MOL000210	Q92731	Estrogen receptor beta	ESR2
Magnolol	MOL000210	P14867	Gamma-aminobutyric acid receptor subunit alpha-1	GABRA1

Magnolol	MOL000210	Q16539	Mitogen-activated protein kinase 14	MAPK14
Magnolol	MOL000210	P49841	Glycogen synthase kinase-3 beta	GSK3B
Magnolol	MOL000210	P27338	Amine oxidase [flavin-containing] B	MAOB
Magnolol	MOL000210	O14757	Serine/threonine-protein kinase Chk1	CHEK1
Magnolol	MOL000210	P07477	Trypsin-1	PRSS1
Magnolol	MOL000210	P34972	Cannabinoid receptor 2	CNR2
Magnolol	MOL000210	Q9GZV3	High affinity choline transporter 1	SLC5A7
Magnolol	MOL000210	P21554	Cannabinoid receptor 1	CNR1
Magnolol	MOL000210	P14867	Gamma-aminobutyric acid receptor subunit alpha-1	GABRA1
Magnolol	MOL000210	P47870	Gamma-aminobutyric acid receptor subunit beta-2	GABRB2
Magnolol	MOL000210	P25929	Neuropeptide Y receptor type 1	NPY1R
Magnolol	MOL000210	P49146	Neuropeptide Y receptor type 2	NPY2R
Magnolol	MOL000210	O43613	Orexin receptor type 1	HCRTR1
Magnolol	MOL000210	O95180	Voltage-dependent T-type calcium channel subunit alpha-1H	CACNA1H
Magnolol	MOL000210	Q9NPC2	Potassium channel subfamily K member 9	KCNK9
Magnolol	MOL000210	P32245	Melanocortin receptor 4	MC4R
Magnolol	MOL000210	P35348	Alpha-1A adrenergic receptor	ADRA1A
Magnolol	MOL000210	P25100	Alpha-1D adrenergic receptor	ADRA1D
Magnolol	MOL000210	P51693	Amyloid-like protein 1	APLP1
Magnolol	MOL000210	O15405	TOX high mobility group box family member 3	TOX3
Magnolol	MOL000210	P49840	Glycogen synthase kinase-3 alpha	GSK3A
Magnolol	MOL000210	Q8NER1	Transient receptor potential cation channel subfamily V member 1	TRPV1
Magnolol	MOL000210	P08588	Beta-1 adrenergic receptor	ADRB1
Magnolol	MOL000210	Q86YN6	Peroxisome proliferator-activated receptor gamma coactivator 1-beta	PPARGC1B
Magnolol	MOL000210	P21918	D	DRD5
Magnolol	MOL000210	P30542	Adenosine receptor A1	ADORA1
Magnolol	MOL000210	Q7Z2W7	Transient receptor potential cation channel subfamily M member 8	TRPM8
Magnolol	MOL000210	O15164	Transcription intermediary factor 1-alpha	TRIM24
Magnolol	MOL000210	P62508	Estrogen-related receptor gamma	ESRRG
Magnolol	MOL000210	P35368	Alpha-1B adrenergic receptor	ADRA1B
Magnolol	MOL000210	P36544	Neuronal acetylcholine receptor subunit alpha-7	CHRNA7
Magnolol	MOL000210	Q8IVL1	Neuron navigator 2	NAV2

Magnolol	MOL000210	Q99801	Homeobox protein Nkx-3.1	NKX3-1
Magnolol	MOL000210	Q15465	Sonic hedgehog protein	SHH
Magnolol	MOL000210	P09936	Ubiquitin carboxyl-terminal hydrolase isozyme L1	UCHL1
Magnolol	MOL000210	P56705	Protein Wnt-4	WNT4
Magnolol	MOL000210	P23634	Plasma membrane calcium-transporting ATPase 4	ATP2B4
Magnolol	MOL000210	P78536	Disintegrin and metalloproteinase domain-containing protein 17	ADAM17
Magnolol	MOL000210	P48436	Transcription factor SOX-9	SOX9
Magnolol	MOL000210	O75762	Transient receptor potential cation channel subfamily A member 1	TRPA1
Magnolol	MOL000210	Q92731	Estrogen receptor beta	ESR2
Magnolol	MOL000210	P18825	Alpha-2C adrenergic receptor	ADRA2C
Magnolol	MOL000210	P01308	Insulin [Cleaved into: Insulin B chain; Insulin A chain]	INS
Magnolol	MOL000210	P01137	Transforming growth factor beta-1 proprotein [Cleaved into: Latency-associated peptide	TGFB1
Magnolol	MOL000210	P41208	Centrin-2	CETN2
Magnolol	MOL000210	Q9UJU2	Lymphoid enhancer-binding factor 1	LEF1
Magnolol	MOL000210	Q08499	cAMP-specific 3',5'-cyclic phosphodiesterase 4D	PDE4D
Magnolol	MOL000210	P49407	Beta-arrestin-1	ARRB1
Magnolol	MOL000210	Q96B67	Arrestin domain-containing protein 3	ARRDC3
Magnolol	MOL000210	P13945	Beta-3 adrenergic receptor	ADRB3
Magnolol	MOL000210	P11473	Vitamin D3 receptor	VDR
Magnolol	MOL000210	P08913	Alpha-2A adrenergic receptor	ADRA2A
Magnolol	MOL000210	P18089	Alpha-2B adrenergic receptor	ADRA2B
Magnolol	MOL000210	P01258	Calcitonin [Cleaved into: Calcitonin; Katacalcin	CALCA
Magnolol	MOL000210	P07550	Beta-2 adrenergic receptor	ADRB2
Magnolol	MOL000210	P51843	Nuclear receptor subfamily 0 group B member 1	NR0B1
Magnolol	MOL000210	P21802	Fibroblast growth factor receptor 2	FGFR2
Magnolol	MOL000210	Q9Y6Q9	Nuclear receptor coactivator 3	NCOA3
Magnolol	MOL000210	Q99527	G-protein coupled estrogen receptor 1	GPER1
Magnolol	MOL000210	P29475	Nitric oxide synthase, brain	NOS1
Magnolol	MOL000210	P09493	Tropomyosin alpha-1 chain	TPM1
Magnolol	MOL000210	P03372	Estrogen receptor	ESR1

Magnolol	MOL000210	P15735	Phosphorylase b kinase gamma catalytic chain, liver/testis isoform	PHKG2
Magnolol	MOL000210	Q92754	Transcription factor AP-2 gamma	TFAP2C
Magnolol	MOL000210	Q15648	Mediator of RNA polymerase II transcription subunit 1	MED1
Magnolol	MOL000210	Q12798	Centrin-1	CETN1
Magnolol	MOL000210	P32121	Beta-arrestin-2	ARRB2
Magnolol	MOL000210	P06401	Progesterone receptor	PGR
Magnolol	MOL000210	P15514	Amphiregulin	AREG
Magnolol	MOL000210	P41221	Protein Wnt-5a	WNT5A
Magnolol	MOL000210	P41220	Regulator of G-protein signaling 2	RGS2
Magnolol	MOL000210	P20800	Endothelin-2	EDN2
Magnolol	MOL000210	P21554	Cannabinoid receptor 1	CNR1
Magnolol	MOL000210	P34972	Cannabinoid receptor 2	CNR2
Hesperidin	MOL007930	Q07812	Apoptosis regulator BAX	BAX
Hesperidin	MOL007930	P42574	Caspase-3	CASP3
Hesperidin	MOL007930	P35354	Prostaglandin G/H synthase 2	PTGS2
Hesperidin	MOL007930	P05362	Intercellular adhesion molecule 1	ICAM1
Hesperidin	MOL007930	P19320	Vascular cell adhesion protein 1	VCAM1
Hesperidin	MOL007930	Q8IVF5	T-lymphoma invasion and metastasis-inducing protein 2	TIAM2
Hesperidin	MOL007930	Q00975	Voltage-dependent N-type calcium channel subunit alpha-1B	CACNA1B
Hesperidin	MOL007930	O60341	Lysine-specific histone demethylase 1A	KDM1A
Hesperidin	MOL007930	Q9GZU7	Carboxy-terminal domain RNA polymerase II polypeptide A small phosphatase 1	CTDSP1
Hesperidin	MOL007930	P25098	Beta-adrenergic receptor kinase 1	GRK2
Hesperidin	MOL007930	Q9Y251	Heparanase	HPSE
Hesperidin	MOL007930	P56817	Beta-secretase 1	BACE1
Hesperidin	MOL007930	P05067	Amyloid-beta precursor protein	APP
Hesperidin	MOL007930	P35610	Sterol O-acyltransferase 1	SOAT1
Hesperidin	MOL007930	P07550	Beta-2 adrenergic receptor	ADRB2
Hesperidin	MOL007930	Q12809	Potassium voltage-gated channel subfamily H member 2	KCNH2
Hesperidin	MOL007930	P55157	Microsomal triglyceride transfer protein large subunit	MTTP
Hesperidin	MOL007930	Q92731	Estrogen receptor beta	ESR2
Hesperidin	MOL007930	P46098	5-hydroxytryptamine receptor 3A	HTR3A
Hesperidin	MOL007930	O75908	Sterol O-acyltransferase 2	SOAT2
Hesperidin	MOL007930	P03372	Estrogen receptor	ESR1

Hesperidin	MOL007930	P11473	Vitamin D3 receptor	VDR
Hesperidin	MOL007930	Q13639	5-hydroxytryptamine receptor 4	HTR4
Hesperidin	MOL007930	P08588	Beta-1 adrenergic receptor	ADRB1
Hesperidin	MOL007930	P28223	5-hydroxytryptamine receptor 2A	HTR2A
Hesperidin	MOL007930	O75469	Nuclear receptor subfamily 1 group I member 2	NR1I2
Liquiritin	MOL004903	P08709	Coagulation factor VII	F7
Liquiritin	MOL004903	P35354	Prostaglandin G/H synthase 2	PTGS2
Liquiritin	MOL004903	P35968	Vascular endothelial growth factor receptor 2	KDR
Liquiritin	MOL004903	P00441	Superoxide dismutase [Cu-Zn]	SOD1
Liquiritin	MOL004903	P07237	Protein disulfide-isomerase	P4HB
Liquiritin	MOL004903	P35610	Sterol O-acyltransferase 1	SOAT1
Liquiritin	MOL004903	P55157	Microsomal triglyceride transfer protein large subunit	MTTP
Liquiritin	MOL004903	O75908	Sterol O-acyltransferase 2	SOAT2
Naringin	MOL005812	P38936	Cyclin-dependent kinase inhibitor 1	CDKN1A
Naringin	MOL005812	O14827	Ras-specific guanine nucleotide-releasing factor 2	RASGRF 2
Naringin	MOL005812	P04049	RAF proto-oncogene serine/threonine-protein kinase	RAF1
Naringin	MOL005812	P11511	Aromatase	CYP19A1
Naringin	MOL005812	P61088	Ubiquitin-conjugating enzyme E2 N	UBE2N
Naringin	MOL005812	Q9Y25 1	Heparanase	HPSE
Naringin	MOL005812	P46721	Solute carrier organic anion transporter family member 1A2	SLCO1A 2
Naringin	MOL005812	P35610	Sterol O-acyltransferase 1	SOAT1
Naringin	MOL005812	P55157	Microsomal triglyceride transfer protein large subunit	MTTP
Naringin	MOL005812	O75908	Sterol O-acyltransferase 2	SOAT2
Thymol	MOL002042	P23219	Prostaglandin G/H synthase 1	PTGS1
Thymol	MOL002042	P11229	Muscarinic acetylcholine receptor M1	CHRM1
Thymol	MOL002042	P08588	Beta-1 adrenergic receptor	ADRB1
Thymol	MOL002042	P18825	Alpha-2C adrenergic receptor	ADRA2C
Thymol	MOL002042	P23975	Sodium-dependent noradrenaline transporter	SLC6A2
Thymol	MOL002042	P35348	Alpha-1A adrenergic receptor	ADRA1A
Thymol	MOL002042	Q01959	Sodium-dependent dopamine transporter	SLC6A3
Thymol	MOL002042	P07550	Beta-2 adrenergic receptor	ADRB2

Thymol	MOL002042	P20309	Muscarinic acetylcholine receptor M3	CHRM3
Thymol	MOL002042	P08172	Muscarinic acetylcholine receptor M2	CHRM2
Thymol	MOL002042	P35368	Alpha-1B adrenergic receptor	ADRA1B
Thymol	MOL002042	P25100	Alpha-1D adrenergic receptor	ADRA1D
Thymol	MOL002042	P08246	Neutrophil elastase	ELANE
Thymol	MOL002042	O75762	Transient receptor potential cation channel subfamily A member 1	TRPA1
Thymol	MOL002042	P11511	Aromatase	CYP19A1
Thymol	MOL002042	P47869	Gamma-aminobutyric acid receptor subunit alpha-2	GABRA2
Thymol	MOL002042	P01375	Tumor necrosis factor	TNF
Thymol	MOL002042	O00591	Gamma-aminobutyric acid receptor subunit pi	GABRP
Thymol	MOL002042	Q9UN88	Gamma-aminobutyric acid receptor subunit theta	GABRQ
Thymol	MOL002042	Q07343	cAMP-specific 3',5'-cyclic phosphodiesterase 4B	PDE4B
Thymol	MOL002042	Q9Y215	Acetylcholinesterase collagenic tail peptide	COLQ
Thymol	MOL002042	P31645	Sodium-dependent serotonin transporter	SLC6A4
Thymol	MOL002042	P35462	D	DRD3
Thymol	MOL002042	Q9NY46	Sodium channel protein type 3 subunit alpha	SCN3A
Thymol	MOL002042	Q92854	Semaphorin-4D	SEMA4D
Thymol	MOL002042	P14174	Macrophage migration inhibitory factor	MIF
Thymol	MOL002042	P48023	Tumor necrosis factor ligand superfamily member 6	FASLG
Thymol	MOL002042	P02741	C-reactive protein [Cleaved into: C-reactive protein	CRP
Thymol	MOL002042	P01584	Interleukin-1 beta	IL1B
Thymol	MOL002042	Q53GD3	Choline transporter-like protein 4	SLC44A4
Thymol	MOL002042	Q15389	Angiopoietin-1	ANGPT1
Thymol	MOL002042	P26678	Cardiac phospholamban	PLN
Thymol	MOL002042	P23327	Sarcoplasmic reticulum histidine-rich calcium-binding protein	HRC
Thymol	MOL002042	Q15847	Adipogenesis regulatory factor	ADIRF
Thymol	MOL002042	P22301	Interleukin-10	IL10

Thymol	MOL002042	P49354	Protein farnesyltransferase/geranylgeranyltransferase type-1 subunit alpha	FNTA
Thymol	MOL002042	P23526	Adenosylhomocysteinase	AHCY
Thymol	MOL002042	O14920	Inhibitor of nuclear factor kappa-B kinase subunit beta	IKBKB
Thymol	MOL002042	Q8NE62	Choline dehydrogenase, mitochondrial	CHDH
Thymol	MOL002042	Q9UL51	Potassium/sodium hyperpolarization-activated cyclic nucleotide-gated channel 2	HCN2
Thymol	MOL002042	O14764	Gamma-aminobutyric acid receptor subunit delta	GABRD
Thymol	MOL002042	P78334	Gamma-aminobutyric acid receptor subunit epsilon	GABRE
Thymol	MOL002042	P48169	Gamma-aminobutyric acid receptor subunit alpha-4	GABRA4
Thymol	MOL002042	P28472	Gamma-aminobutyric acid receptor subunit beta-3	GABRB3
Thymol	MOL002042	P06276	Cholinesterase	BCHE
Thymol	MOL002042	P01374	Lymphotoxin-alpha	LTA
Thymol	MOL002042	P21917	D	DRD4
Thymol	MOL002042	P21918	D	DRD5
Thymol	MOL002042	P52926	High mobility group protein HMGI-C	HMGA2
Thymol	MOL002042	Q02297	Pro-neuregulin-1, membrane-bound isoform	NRG1
Thymol	MOL002042	Q9UI17	Dimethylglycine dehydrogenase, mitochondrial	DMGDH
Thymol	MOL002042	P17931	Galectin-3	LGALS3
Thymol	MOL002042	O15455	Toll-like receptor 3	TLR3
Thymol	MOL002042	Q15465	Sonic hedgehog protein	SHH
Thymol	MOL002042	P29275	Adenosine receptor A2b	ADORA2B
Thymol	MOL002042	O14965	Aurora kinase A	AURKA
Thymol	MOL002042	Q9UQ16	Dynamin-3	DNM3
Thymol	MOL002042	P61812	Transforming growth factor beta-2 proprotein	TGFB2
Thymol	MOL002042	P30411	B2 bradykinin receptor	BDKRB2
Thymol	MOL002042	P55286	Cadherin-8	CDH8
Thymol	MOL002042	P05981	Serine protease hepsin	HPN
Thymol	MOL002042	P02647	Apolipoprotein A-I	APOA1

Thymol	MOL002042	P14902	Indoleamine 2,3-dioxygenase 1	IDO1
Thymol	MOL002042	P48436	Transcription factor SOX-9	SOX9
Thymol	MOL002042	Q14432	cGMP-inhibited 3',5'-cyclic phosphodiesterase A	PDE3A
Thymol	MOL002042	Q99250	Sodium channel protein type 2 subunit alpha	SCN2A
Thymol	MOL002042	P47870	Gamma-aminobutyric acid receptor subunit beta-2	GABRB2
Thymol	MOL002042	P31644	Gamma-aminobutyric acid receptor subunit alpha-5	GABRA5
Thymol	MOL002042	P18507	Gamma-aminobutyric acid receptor subunit gamma-2	GABRG2
Thymol	MOL002042	P19793	Retinoic acid receptor RXR-alpha	RXRA
Thymol	MOL002042	P34969	5-hydroxytryptamine receptor 7	HTR7
Thymol	MOL002042	P21728	D	DRD1
Thymol	MOL002042	O76074	cGMP-specific 3',5'-cyclic phosphodiesterase	PDE5A
Thymol	MOL002042	P04233	HLA class II histocompatibility antigen gamma chain	CD74
Thymol	MOL002042	Q99801	Homeobox protein Nkx-3.1	NKX3-1
Thymol	MOL002042	O43474	Krueppel-like factor 4	KLF4
Thymol	MOL002042	P01588	Erythropoietin	EPO
Thymol	MOL002042	Q13546	Receptor-interacting serine/threonine-protein kinase 1	RIPK1
Thymol	MOL002042	P49419	Alpha-aminoadipic semialdehyde dehydrogenase	ALDH7A 1
Thymol	MOL002042	P09544	Protein Wnt-2	WNT2
Thymol	MOL002042	P06734	Low affinity immunoglobulin epsilon Fc receptor	FCER2
Thymol	MOL002042	Q9Y4G 8	Rap guanine nucleotide exchange factor 2	RAPGEF 2
Thymol	MOL002042	P50553	Achaete-scute homolog 1	ASCL1
Thymol	MOL002042	Q15788	Nuclear receptor coactivator 1	NCOA1
Thymol	MOL002042	Q03164	Histone-lysine N-methyltransferase 2A	KMT2A
Thymol	MOL002042	Q92974	Rho guanine nucleotide exchange factor 2	ARHGEF 2
Thymol	MOL002042	P35222	Catenin beta-1	CTNNB1
Thymol	MOL002042	Q13555	Calcium/calmodulin-dependent protein kinase type II subunit gamma	CAMK2G
Thymol	MOL002042	P18505	Gamma-aminobutyric acid receptor subunit beta-1	GABRB1

Thymol	MOL002042	P34903	Gamma-aminobutyric acid receptor subunit alpha-3	GABRA3
Thymol	MOL002042	P22303	Acetylcholinesterase	ACHE
Thymol	MOL002042	Q16445	Gamma-aminobutyric acid receptor subunit alpha-6	GABRA6
Thymol	MOL002042	Q13370	cGMP-inhibited 3',5'-cyclic phosphodiesterase B	PDE3B
Thymol	MOL002042	Q01959	Sodium-dependent dopamine transporter	SLC6A3
Thymol	MOL002042	P08908	5-hydroxytryptamine receptor 1A	HTR1A
Thymol	MOL002042	P23975	Sodium-dependent noradrenaline transporter	SLC6A2
Thymol	MOL002042	P02652	Apolipoprotein A-II	APOA2
Thymol	MOL002042	P02790	Hemopexin	HPX
Thymol	MOL002042	O95343	Homeobox protein SIX3	SIX3
Thymol	MOL002042	Q8NHL6	Leukocyte immunoglobulin-like receptor subfamily B member 1	LILRB1
Thymol	MOL002042	Q13163	Dual specificity mitogen-activated protein kinase kinase 5	MAP2K5
Thymol	MOL002042	O15520	Fibroblast growth factor 10	FGF10
Thymol	MOL002042	Q13158	FAS-associated death domain protein	FADD
Thymol	MOL002042	P16989	Y-box-binding protein 3	YBX3
Thymol	MOL002042	Q93097	Protein Wnt-2b	WNT2B
Thymol	MOL002042	P58753	Toll/interleukin-1 receptor domain-containing adapter protein	TIRAP
Thymol	MOL002042	Q6UWR7	Glycerophosphocholine cholinephosphodiesterase ENPP6	ENPP6
Thymol	MOL002042	P05231	Interleukin-6	IL6
Thymol	MOL002042	Q9HC29	Nucleotide-binding oligomerization domain-containing protein 2	NOD2
Thymol	MOL002042	P05112	Interleukin-4	IL4
Thymol	MOL002042	P35790	Choline kinase alpha	CHKA
Thymol	MOL002042	P25445	Tumor necrosis factor receptor superfamily member 6	FAS
Thymol	MOL002042	Q99928	Gamma-aminobutyric acid receptor subunit gamma-3	GABRG3
Thymol	MOL002042	Q8N1C3	Gamma-aminobutyric acid receptor subunit gamma-1	GABRG1
Thymol	MOL002042	P35499	Sodium channel protein type 4 subunit alpha	SCN4A
Thymol	MOL002042	P14867	Gamma-aminobutyric acid receptor subunit alpha-1	GABRA1

Thymol	MOL002042	Q08499	cAMP-specific 3',5'-cyclic phosphodiesterase 4D	PDE4D
Thymol	MOL002042	P14416	D	DRD2
Thymol	MOL002042	P09172	Dopamine beta-hydroxylase	DBH
Thymol	MOL002042	P35498	Sodium channel protein type 1 subunit alpha	SCN1A
Thymol	MOL002042	Q9GZV3	High affinity choline transporter 1	SLC5A7
Thymol	MOL002042	Q8IXJ6	NAD-dependent protein deacetylase sirtuin-2	SIRT2
Thymol	MOL002042	Q9Y3Q4	Potassium/sodium hyperpolarization-activated cyclic nucleotide-gated channel 4	HCN4
Thymol	MOL002042	P55160	Nck-associated protein 1-like	NCKAP1L
Thymol	MOL002042	Q9BQI3	Eukaryotic translation initiation factor 2-alpha kinase 1	EIF2AK1
Thymol	MOL002042	P56730	Neurotrypsin	PRSS12
Thymol	MOL002042	P21802	Fibroblast growth factor receptor 2	FGFR2
Thymol	MOL002042	P18510	Interleukin-1 receptor antagonist protein	IL1RN
Thymol	MOL002042	P01579	Interferon gamma	IFNG
Thymol	MOL002042	O75116	Rho-associated protein kinase 2	ROCK2
Thymol	MOL002042	Q13557	Calcium/calmodulin-dependent protein kinase type II subunit delta	CAMK2D
Thymol	MOL002042	Q13464	Rho-associated protein kinase 1	ROCK1
Thymol	MOL002042	P12644	Bone morphogenetic protein 4	BMP4
Thymol	MOL002042	O00468	Agrin [Cleaved into: Agrin N-terminal 110 kDa subunit; Agrin C-terminal 110 kDa subunit; Agrin C-terminal 90 kDa fragment	AGRN
Thymol	MOL002042	O00408	cGMP-dependent 3',5'-cyclic phosphodiesterase	PDE2A
Thymol	MOL002042	Q05586	Glutamate receptor ionotropic, NMDA 1	GRIN1
Thymol	MOL002042	O75762	Transient receptor potential cation channel subfamily A member 1	TRPA1
Imperatorin	MOL001941	P11229	Muscarinic acetylcholine receptor M1	CHRM1
Imperatorin	MOL001941	P35354	Prostaglandin G/H synthase 2	PTGS2
Imperatorin	MOL001941	P14867	Gamma-aminobutyric acid receptor subunit alpha-1	GABRA1
Imperatorin	MOL001941	P27338	Amine oxidase [flavin-containing] B	MAOB

Imperatorin	MOL001941	P01584	Interleukin-1 beta	IL1B
Imperatorin	MOL001941	Q16678	Cytochrome P450 1B1	CYP1B1
Imperatorin	MOL001941	P35869	Aryl hydrocarbon receptor	AHR
Imperatorin	MOL001941	P08684	Cytochrome P450 3A4	CYP3A4
Imperatorin	MOL001941	Q14994	Nuclear receptor subfamily 1 group I member 3	NR1I3
Imperatorin	MOL001941	P53350	Serine/threonine-protein kinase PLK1	PLK1
Imperatorin	MOL001941	P08183	ATP-dependent translocase ABCB1	ABCB1
Imperatorin	MOL001941	P56817	Beta-secretase 1	BACE1
Imperatorin	MOL001941	O75469	Nuclear receptor subfamily 1 group I member 2	NR1I2
Imperatorin	MOL001941	P06401	Progesterone receptor	PGR
Isoimperatorin	MOL001942	P35354	Prostaglandin G/H synthase 2	PTGS2
Isoimperatorin	MOL001942	Q16678	Cytochrome P450 1B1	CYP1B1
Isoimperatorin	MOL001942	P35869	Aryl hydrocarbon receptor	AHR

Table S2: ALI related targets.

**number**	**ALI related targets**
1	GNB1
2	TAL1
3	CPT2
4	RBM15
5	COPA
6	FMO2
7	FASLG
8	LPIN1
9	DNMT3A
10	HADHA
11	KCNK3
12	SFTPB
13	RANBP2
14	CASP8
15	BMPR2
16	CPS1
17	FARSB
18	DLEC1
19	GATA2
20	MME
21	TERC
22	PIK3CA
23	EIF4G1

24	LPP
25	SLC34A2
26	CHIC2
27	KIT
28	IGFBP7
29	RAP1GDS1
30	TLR3
31	TERT
32	LUCAT1
33	ACSL6
34	IRF1
35	NPM1
36	DEK
37	HLA-B
38	MYB
39	PRKN
40	EGFR
41	CAV1
42	BRAF
43	BLACE
44	SFTPC
45	STAR
46	NBN
47	BAALC
48	JAK2
49	PAX5
50	TAL2
51	ALAD
52	SET
53	NUP214
54	MLLT10
55	MAP3K8
56	ERCC6
57	SFTPA2
58	SFTPA1
59	SFTPD
60	MUC5B
61	SLC22A18
62	LMO1
63	LMO2
64	FAM111B
65	MEN1
66	MALAT1

67	UNC93B1
68	NUMA1
69	PICALM
70	MMP1
71	PPP2R1B
72	ZBTB16
73	HMBS
74	NINJ2
75	ETV6
76	KRAS
77	MARS1
78	TBK1
79	ALDH2
80	ZCCHC8
81	FLT3
82	SMAD9
83	RB1
84	TRAF3
85	NSMCE3
86	EIF2AK4
87	PML
88	CHRNA5
89	CHRNA3
90	ABCA3
91	MEFV
92	CREBBP
93	PARN
94	MYH11
95	CBFB
96	FOXF1
97	ERBB2
98	RARA
99	STAT5B
100	STAT3
101	ITGA3
102	MPO
103	TBX4
104	SH3GL1
105	TICAM1
106	FARSA
107	LYL1
108	CEBPA
109	SARS2

110	CYP2A6
111	TGFB1
112	BAX
113	IRF3
114	SLC13A3
115	RTEL1
116	RUNX1
117	BCR
118	HMOX1
119	CSF2RB
120	CSF2RA
121	SERPINE1
122	F3
123	IL1B
124	NQO1
125	C3
126	ELANE
127	IL10
128	GJA1
129	ADRA2A
130	ADAR
131	GOT1
132	IL13
133	OPA1
134	P2RY12
135	ICAM1
136	PAK1
137	ALB
138	PLA2G2A
139	EDN1
140	IL17A
141	PPARG
142	MST1R
143	GSR
144	FADD
145	MMP9
146	MFN2
147	TNF
148	ASCL1
149	FCGR2A
150	NOD2
151	FAS
152	EPHX1

153	TIMP2
154	CYP1A1
155	AKAP12
156	SLPI
157	AGER
158	NOS2
159	HMGB1
160	AQP5
161	PECAM1
162	IL1RL1
163	F2
164	MFN1
165	NFE2L2
166	FCGR2B
167	TXN
168	FLT1
169	ITGB2
170	PAWR
171	MMP2
172	CXCL2
173	PLAU
174	CAT
175	CCL4
176	BCL2
177	IL18
178	CCL2
179	VIP
180	CASP3
181	MIR96
182	THBD
183	FGA
184	IL2
185	APOA1
186	CXCL6
187	MIR330
188	IL4
189	SCGB1A1
190	MMP7
191	KDR
192	TFF1
193	ACVR1
194	IFNG
195	MYLK

196	FCGR1A
197	TLR4
198	EPHX2
199	IL1RN
200	TNFAIP6
201	IL6
202	PROCR
203	TFF2
204	PTGS2
205	VEGFA
206	XDH

Table S3: Common targets between HZOL and ALI.

number	Potential targets for HZOL treatment of ALI
1	FASLG
2	KDR
3	TNF
4	IL10
5	ELANE
6	PTGS2
7	IL1B
8	FADD
9	FAS
10	IFNG
11	IL4
12	NOD2
13	APOA1
14	ASCL1
15	ADRA2A
16	TGFB1
17	ICAM1
18	BAX
19	TLR3
20	IL6
21	CASP3
22	IL1RN

Table S4: Detail information of 8 clusters of Functional annotation clustering results.

**Annotation Cluster 1**	**Enrichment Score: 5.509280338614713**											
Category	Term	Count	%	PValue	Genes	List Total	Pop Hits	Pop Total	Fold Enrichment	Bonferroni	Benjamini	FDR
GOTERM_BP_DIRECT	GO:0045429~positive regulation of nitric oxide biosynthetic process	6	27.27273	1.71E-09	IL6, IFNG, IL1B, PTGS2, TNF, ICAM1	22	43	16792	106.5032	1.59E-06	2.03E-07	1.85E-07
GOTERM_BP_DIRECT	GO:0042346~positive regulation of NF-kappaB import into nucleus	4	18.18182	2.21E-06	IL1B, PTGS2, TNF, TLR3	22	21	16792	145.3853	0.002049	8.20E-05	7.49E-05
GOTERM_BP_DIRECT	GO:0031622~positive regulation of fever generation	3	13.63636	1.49E-05	IL1B, PTGS2, TNF	22	5	16792	457.9636	0.013698	4.06E-04	3.70E-04
KEGG_PATHWAY	hsa04064:NF-kappa B signaling pathway	4	18.18182	0.001636	IL1B, PTGS2, TNF, ICAM1	20	87	6879	15.81379	0.149601	0.004907	0.002775

**Annotation Cluster 2**	**Enrichment Score: 4.794343089001299**											
Category	Term	Count	%	PValue	Genes	List Total	Pop Hits	Pop Total	Fold Enrichment	Bonferroni	Benjamini	FDR
KEGG_PATHWAY	hsa05330:Allograft rejection	6	27.27273	3.74E-08	IL10, IL4, IFNG, FAS, FASLG, TNF	20	37	6879	55.77568	3.71E-06	3.37E-07	1.91E-07

BIOCARTA	h_asbcellPathway:Antigen Dependent B Cell Activation	4	18.18182	3.05E-04	IL10, IL4, FAS, FASLG	19	13	1625	26.31579	0.024402	0.00716	0.006187
KEGG_PATHWAY	hsa05320:Autoimmune thyroid disease	4	18.18182	3.63E-04	IL10, IL4, FAS, FASLG	20	52	6879	26.45769	0.035258	0.001329	7.52E-04

**Annotation Cluster 3**	**Enrichment Score: 4.778139586872909**											
Category	Term	Count	%	PValue	Genes	List Total	Pop Hits	Pop Total	Fold Enrichment	Bonferroni	Benjamini	FDR
UP_KEYWORDS	Glycoprotein	16	72.72727	1.97E-06	IL10, IL1RN, TGFB1, APOA1, FASLG, PTGS2, TNF, ADRA2A, ICAM1, IL4, IL6, IFNG, KDR, FAS, ELANE, TLR3	22	4551	20581	3.288947	1.95E-04	6.50E-05	6.04E-05
UP_KEYWORDS	Disulfide bond	14	63.63636	4.07E-06	IL10, IL1RN, TGFB1, FASLG, PTGS2, TNF, ADRA2A, ICAM1, IL4, IL6, KDR, FAS, ELANE, TLR3	22	3434	20581	3.81392	4.03E-04	1.01E-04	9.37E-05
UP_SEQ_FEATURE	disulfide bond	13	59.09091	7.10E-06	IL10, IL1RN, TGFB1, FASLG, PTGS2, TNF, ADRA2A, ICAM1, IL4, IL6, FAS, ELANE, TLR3	22	2917	20063	4.064247	8.38E-04	8.45E-04	8.45E-04
UP_SEQ_FEATURE	signal peptide	13	59.09091	3.01E-05	IL10, IL1RN, TGFB1, APOA1, PTGS2, ICAM1, IL4, IL6, IFNG, KDR, FAS, ELANE, TLR3	22	3346	20063	3.543159	0.003545	0.001791	0.001791

UP_SEQ_FEATURE	glycosylation site:N-linked (GlcNAc...)	14	63.63636	5.85E-05	IL10, IL1RN, TGFB1, FASLG, PTGS2, ADRA2A, ICAM1, IL4, IL6, IFNG, KDR, FAS, ELANE, TLR3	22	4234	20063	3.015438	0.006875	0.002319	0.002319
UP_KEYWORDS	Signal	13	59.09091	2.14E-04	IL10, IL1RN, TGFB1, APOA1, PTGS2, ICAM1, IL4, IL6, IFNG, KDR, FAS, ELANE, TLR3	22	4160	20581	2.923438	0.020955	0.004235	0.003936

**Annotation Cluster 4**	**Enrichment Score: 4.627778068430032**											
Category	Term	Count	%	PValue	Genes	List Total	Pop Hits	Pop Total	Fold Enrichment	Bonferroni	Benjamini	FDR
GOTERM_BP_DIRECT	GO:0051092~positive regulation of NF-kappaB transcription factor activity	7	31.81818	1.08E-08	IL6, TGFB1, IL1B, NOD2, TNF, TLR3, ICAM1	22	133	16792	40.17225	1.01E-05	1.12E-06	1.02E-06
GOTERM_BP_DIRECT	GO:0032757~positive regulation of interleukin-8 production	5	22.72727	2.65E-08	IL1B, FADD, NOD2, TNF, TLR3	22	26	16792	146.7832	2.46E-05	1.89E-06	1.73E-06
GOTERM_BP_DIRECT	GO:0032755~positive regulation of interleukin-6 production	5	22.72727	2.60E-07	IL6, IL1B, NOD2, TNF, TLR3	22	45	16792	84.80808	2.42E-04	1.34E-05	1.23E-05
GOTERM_BP_DIRECT	GO:0071407~cellular response to organic cyclic compound	5	22.72727	7.87E-07	TGFB1, IL1B, CASP3, NOD2, TNF	22	59	16792	64.68413	7.30E-04	3.48E-05	3.17E-05
GOTERM_BP_DIRECT	GO:0042346~positive regulation of NF-kappaB import into nucleus	4	18.18182	2.21E-06	IL1B, PTGS2, TNF, TLR3	22	21	16792	145.3853	0.002049	8.20E-05	7.49E-05

GOTERM_BP_DIRECT	GO:0010628~positive regulation of gene expression	6	27.27273	1.48E-05	IL6, TGFB1, IFNG, IL1B, TNF, TLR3	22	262	16792	17.47953	0.013605	4.06E-04	3.70E-04
GOTERM_BP_DIRECT	GO:0046330~positive regulation of JNK cascade	4	18.18182	7.00E-05	IL1B, NOD2, TNF, TLR3	22	65	16792	46.97063	0.06294	0.001413	0.00129
KEGG_PATHWAY	hsa04620:Toll-like receptor signaling pathway	5	22.72727	1.73E-04	IL6, IL1B, FADD, TNF, TLR3	20	106	6879	16.22406	0.01696	7.13E-04	4.03E-04
GOTERM_BP_DIRECT	GO:0032722~positive regulation of chemokine production	3	13.63636	2.00E-04	IL6, TNF, TLR3	22	17	16792	134.6952	0.169646	0.003507	0.003201
KEGG_PATHWAY	hsa05134:Legionellosis	4	18.18182	4.05E-04	IL6, IL1B, CASP3, TNF	20	54	6879	25.47778	0.039351	0.001434	8.11E-04
KEGG_PATHWAY	hsa04621:NOD-like receptor signaling pathway	4	18.18182	4.52E-04	IL6, IL1B, NOD2, TNF	20	56	6879	24.56786	0.043726	0.001541	8.72E-04
GOTERM_BP_DIRECT	GO:0032729~positive regulation of interferon-gamma production	3	13.63636	0.001491	IL1B, FADD, TNF	22	46	16792	49.77866	0.749698	0.02097	0.01914
KEGG_PATHWAY	hsa04640:Hematopoietic cell lineage	4	18.18182	0.001636	IL4, IL6, IL1B, TNF	20	87	6879	15.81379	0.149601	0.004907	0.002775

GOTERM_BP_DIRECT	GO:0000187~activation of MAPK activity	3	13.63636	0.007806	IL1B, NOD2, TNF	22	107	16792	21.40017	0.999305	0.080484	0.073459

**Annotation Cluster 5**	**Enrichment Score: 4.614158604382831**											
Category	Term	Count	%	PValue	Genes	List Total	Pop Hits	Pop Total	Fold Enrichment	Bonferroni	Benjamini	FDR
GOTERM_BP_DIRECT	GO:0097191~extrinsic apoptotic signaling pathway	8	36.36364	4.10E-14	TGFB1, IFNG, FAS, BAX, FASLG, FADD, TNF, TLR3	22	42	16792	145.3853	3.80E-11	3.80E-11	3.47E-11
GOTERM_BP_DIRECT	GO:0097527~necroptotic signaling pathway	5	22.72727	2.71E-11	FAS, FASLG, FADD, TNF, TLR3	22	6	16792	636.0606	2.51E-08	1.26E-08	1.15E-08
KEGG_PATHWAY	hsa04668:TNF signaling pathway	9	40.90909	1.73E-10	IL6, IL1B, CASP3, FAS, FADD, NOD2, PTGS2, TNF, ICAM1	20	107	6879	28.93037	1.71E-08	4.92E-09	2.78E-09
GOTERM_BP_DIRECT	GO:0097192~extrinsic apoptotic signaling pathway in absence of ligand	6	27.27273	4.98E-10	IL4, IL1B, CASP3, FAS, BAX, FADD	22	34	16792	134.6952	4.62E-07	9.24E-08	8.43E-08
GOTERM_BP_DIRECT	GO:0043065~positive regulation of apoptotic process	9	40.90909	1.57E-09	IL6, TGFB1, FAS, BAX, FASLG, FADD, PTGS2, TNF, TLR3	22	300	16792	22.89818	1.46E-06	2.03E-07	1.85E-07

KEGG_PATHWAY	hsa05161:Hepatitis B	9	40.90909	2.00E-09	IL6, TGFB1, CASP3, FAS, BAX, FASLG, FADD, TNF, TLR3	20	145	6879	21.34862	1.98E-07	3.29E-08	1.86E-08
KEGG_PATHWAY	hsa05168:Herpes simplex infection	9	40.90909	1.27E-08	IL6, IFNG, IL1B, CASP3, FAS, FASLG, FADD, TNF, TLR3	20	183	6879	16.91557	1.25E-06	1.57E-07	8.87E-08
KEGG_PATHWAY	hsa05332:Graft-versus-host disease	6	27.27273	2.05E-08	IL6, IFNG, IL1B, FAS, FASLG, TNF	20	33	6879	62.53636	2.03E-06	2.26E-07	1.28E-07
GOTERM_BP_DIRECT	GO:0097190~apoptotic signaling pathway	6	27.27273	2.26E-08	CASP3, FAS, BAX, FASLG, FADD, TLR3	22	71	16792	64.50192	2.10E-05	1.75E-06	1.60E-06
GOTERM_BP_DIRECT	GO:0006919~activation of cysteine-type endopeptidase activity involved in apoptotic process	6	27.27273	4.99E-08	CASP3, FAS, BAX, FASLG, FADD, TNF	22	83	16792	55.17634	4.63E-05	3.31E-06	3.02E-06
KEGG_PATHWAY	hsa04932:Non-alcoholic fatty liver disease (NAFLD)	8	36.36364	8.63E-08	IL6, TGFB1, IL1B, CASP3, FAS, BAX, FASLG, TNF	20	151	6879	18.22252	8.55E-06	7.12E-07	4.03E-07
GOTERM_CC_DIRECT	GO:0009897~external side of plasma membrane	7	31.81818	1.11E-07	IL4, IL6, IFNG, FAS, FASLG, TNF, ICAM1	22	213	18224	27.22322	8.91E-06	4.45E-06	4.01E-06
GOTERM_BP_DIRECT	GO:0008625~extrinsic apoptotic signaling	5	22.72727	1.30E-07	FAS, BAX, FASLG, FADD, TNF	22	38	16792	100.4306	1.20E-04	7.53E-06	6.87E-06

	pathway via death domain receptors											
GOTERM_BP_DIRECT	GO:0097296~activation of cysteine-type endopeptidase activity involved in apoptotic signaling pathway	4	18.18182	4.78E-07	FAS, BAX, FASLG, FADD	22	13	16792	234.8531	4.44E-04	2.34E-05	2.13E-05
KEGG_PATHWAY	hsa04210:Apoptosis	6	27.27273	5.33E-07	CASP3, FAS, BAX, FASLG, FADD, TNF	20	62	6879	33.28548	5.27E-05	3.30E-06	1.86E-06
GOTERM_CC_DIRECT	GO:0045121~membrane raft	6	27.27273	3.09E-06	CASP3, KDR, FAS, FADD, TNF, ICAM1	22	206	18224	24.1271	2.47E-04	6.17E-05	5.56E-05
KEGG_PATHWAY	hsa04940:Type I diabetes mellitus	5	22.72727	4.35E-06	IFNG, IL1B, FAS, FASLG, TNF	20	42	6879	40.94643	4.31E-04	2.40E-05	1.35E-05
GOTERM_BP_DIRECT	GO:1902042~negative regulation of extrinsic apoptotic signaling pathway via death domain receptors	4	18.18182	8.98E-06	FAS, FASLG, FADD, ICAM1	22	33	16792	92.51791	0.008297	2.78E-04	2.53E-04
KEGG_PATHWAY	hsa04650:Natural killer cell mediated cytotoxicity	6	27.27273	1.54E-05	IFNG, CASP3, FAS, FASLG, TNF, ICAM1	20	122	6879	16.91557	0.001524	8.03E-05	4.54E-05

GOTERM_CC_DIRECT	GO:0031264~death-inducing signaling complex	3	13.63636	2.65E-05	CASP3, FAS, FADD	22	7	18224	355.013	0.002115	3.53E-04	3.18E-04
GOTERM_BP_DIRECT	GO:0032496~response to lipopolysaccharide	5	22.72727	4.61E-05	CASP3, FAS, FASLG, PTGS2, ELANE	22	164	16792	23.27051	0.041889	0.001044	9.53E-04
GOTERM_BP_DIRECT	GO:0006915~apoptotic process	7	31.81818	5.08E-05	IFNG, IL1B, CASP3, FAS, BAX, FASLG, FADD	22	567	16792	9.42312	0.046032	0.001122	0.001024
KEGG_PATHWAY	hsa05200:Pathways in cancer	8	36.36364	5.21E-05	IL6, TGFB1, CASP3, FAS, BAX, FASLG, FADD, PTGS2	20	393	6879	7.001527	0.005144	2.34E-04	1.33E-04
GOTERM_BP_DIRECT	GO:0042981~regulation of apoptotic process	5	22.72727	1.27E-04	TGFB1, FAS, BAX, FADD, NOD2	22	213	16792	17.9172	0.111314	0.00236	0.002154
KEGG_PATHWAY	hsa05205:Proteoglycans in cancer	6	27.27273	1.65E-04	TGFB1, CASP3, KDR, FAS, FASLG, TNF	20	200	6879	10.3185	0.016181	7.09E-04	4.01E-04
GOTERM_MF_DIRECT	GO:0005123~death receptor binding	3	13.63636	1.75E-04	CASP3, FASLG, FADD	22	16	16881	143.8722	0.018383	0.006775	0.006263
GOTERM_BP_DIRECT	GO:1902041~regulation of extrinsic apoptotic signaling pathway via death domain receptors	3	13.63636	2.00E-04	FAS, FASLG, FADD	22	17	16792	134.6952	0.169646	0.003507	0.003201

GOTERM_MF_DIRECT	GO:0002020~protease binding	4	18.18182	2.56E-04	CASP3, FADD, TNF, ELANE	22	101	16881	30.38884	0.026738	0.006775	0.006263
KEGG_PATHWAY	hsa04010:MAPK signaling pathway	6	27.27273	4.92E-04	TGFB1, IL1B, CASP3, FAS, FASLG, TNF	20	253	6879	8.156917	0.047578	0.001624	9.19E-04
BIOCARTA	h_hsp27Pathway:Stress Induction of HSP Regulation	4	18.18182	5.85E-04	CASP3, FAS, FASLG, TNF	19	16	1625	21.38158	0.046274	0.009473	0.008187
GOTERM_MF_DIRECT	GO:0005164~tumor necrosis factor receptor binding	3	13.63636	5.86E-04	FASLG, FADD, TNF	22	29	16881	79.37774	0.060285	0.012432	0.011494
KEGG_PATHWAY	hsa05010:Alzheimer's disease	5	22.72727	9.99E-04	IL1B, CASP3, FAS, FADD, TNF	20	168	6879	10.23661	0.094175	0.003189	0.001804
GOTERM_BP_DIRECT	GO:0043123~positive regulation of I-kappaB kinase/NF-kappaB signaling	4	18.18182	0.001013	FASLG, FADD, NOD2, TNF	22	161	16792	18.9633	0.609742	0.015169	0.013845
GOTERM_MF_DIRECT	GO:0042802~identical protein binding	6	27.27273	0.001907	FAS, BAX, APOA1, FADD, TNF, TLR3	22	749	16881	6.146741	0.183221	0.028885	0.026705
UP_KEYWORDS	Apoptosis	5	22.72727	0.001914	CASP3, FAS, BAX, FASLG, FADD	22	536	20581	8.726679	0.172743	0.027066	0.025152

BBID	46.P13K_PTEN	4	18.1818 2	0.003261	CASP3, FAS, BAX, FADD	12	12	388	10.77778	0.153426	0.016303	0.007825
BIOCARTA	h_hivnefPathway:HIV-I Nef: negative effector of Fas and TNF	5	22.7272 7	0.003707	CASP3, FAS, FASLG, FADD, TNF	19	61	1625	7.010354	0.259794	0.050045	0.043249
BIOCARTA	h_fasPathway:FAS signaling pathway (CD95)	4	18.1818 2	0.004633	CASP3, FAS, FASLG, FADD	19	32	1625	10.69079	0.313513	0.05012	0.043313
INTERPRO	IPR011029:Death-like domain	3	13.6363 6	0.005006	FAS, FADD, NOD2	22	94	18559	26.92311	0.350547	0.107636	0.101378
UP_SEQ_FEATURE	mutagenesis site	8	36.3636 4	0.005346	IL6, BAX, FASLG, FADD, NOD2, TNF, ADRA2A, TLR3	22	2191	20063	3.32982	0.468719	0.127224	0.127224
BBID	38.Cell_cycle_arrest_and_apoptosis_ceramide	3	13.6363 6	0.006989	CASP3, FAS, TNF	12	5	388	19.4	0.300725	0.026882	0.012904
KEGG_PATHWAY	hsa05014:Amyotrophic lateral sclerosis (ALS)	3	13.6363 6	0.008182	CASP3, BAX, TNF	20	50	6879	20.637	0.556641	0.019287	0.01091
GOTERM_BP_DIRECT	GO:0033209~tumor necrosis factor-mediated signaling pathway	3	13.6363 6	0.009423	FAS, FASLG, TNF	22	118	16792	19.40524	0.999847	0.093392	0.08524

GOTERM_BP_DIRECT	GO:0007165~signal transduction	6	27.27273	0.012552	IL1B, FAS, FASLG, FADD, ADRA2A, TLR3	22	1161	16792	3.944562	0.999992	0.112007	0.10223
KEGG_PATHWAY	hsa04115:p53 signaling pathway	3	13.63636	0.014362	CASP3, FAS, BAX	20	67	6879	15.40075	0.761206	0.032315	0.018279
BIOCARTA	h_pmlPathway:Regulation of transcriptional activity by PML	3	13.63636	0.015969	FAS, FASLG, TNF	19	18	1625	14.25439	0.728542	0.099501	0.085988
BIOCARTA	h_tnfr1Pathway:TNFR1 Signaling Pathway	3	13.63636	0.041977	CASP3, FADD, TNF	19	30	1625	8.552632	0.968993	0.200007	0.172846
BBID	72.IAP_interaction_with_cell_death_pathways	3	13.63636	0.062755	CASP3, FAS, FADD	12	15	388	6.466667	0.963313	0.165144	0.079269
BIOCARTA	h_keratinocytePathway:Keratinocyte Differentiation	3	13.63636	0.096841	FAS, FASLG, TNF	19	48	1625	5.345395	0.999739	0.389118	0.336275
GOTERM_CC_DIRECT	GO:0005829~cytosol	7	31.81818	0.168369	IL1B, CASP3, FAS, BAX, APOA1, FADD, NOD2	22	3315	18224	1.749184	1	0.748306	0.673475

**Annotation Cluster 6**	**Enrichment Score: 4.356604954003153**											
Category	Term	Count	%	PValue	Genes	List Total	Pop Hits	Pop Total	Fold Enrichment	Bonferroni	Benjamini	FDR

KEGG_PATHWAY	hsa05143:African trypanosomiasis	9	40.9090 9	8.14E-15	IL10, IL6, IFNG, IL1B, FAS, APOA1, FASLG, TNF, ICAM1	20	33	6879	93.80455	8.02E-13	8.06E-13	4.56 E-13
GOTERM_MF_DIRECT	GO:0005125~cytokine activity	9	40.9090 9	2.16E-11	IL10, IL4, IL6, IL1RN, TGFB1, IFNG, IL1B, FASLG, TNF	22	176	16881	39.23786	2.29E-09	2.29E-09	2.11 E-09
KEGG_PATHWAY	hsa05142:Chagas disease (American trypanosomiasis)	9	40.9090 9	1.37E-10	IL10, IL6, TGFB1, IFNG, IL1B, FAS, FASLG, FADD, TNF	20	104	6879	29.7649	1.36E-08	4.92E-09	2.78 E-09
KEGG_PATHWAY	hsa05321:Inflammatory bowel disease (IBD)	8	36.3636 4	1.99E-10	IL10, IL4, IL6, TGFB1, IFNG, IL1B, NOD2, TNF	20	64	6879	42.99375	1.97E-08	4.92E-09	2.78 E-09
KEGG_PATHWAY	hsa05152:Tuberculosis	10	45.4545 5	2.99E-10	IL10, IL6, TGFB1, IFNG, IL1B, CASP3, BAX, FADD, NOD2, TNF	20	177	6879	19.4322	2.96E-08	5.92E-09	3.35 E-09
GOTERM_BP_DIRECT	GO:0045080~positive regulation of chemokine biosynthetic process	5	22.7272 7	3.78E-10	IL4, IFNG, IL1B, TNF, TLR3	22	10	16792	381.6364	3.50E-07	8.76E-08	8.00 E-08
GOTERM_BP_DIRECT	GO:0045429~positive regulation of nitric oxide biosynthetic process	6	27.2727 3	1.71E-09	IL6, IFNG, IL1B, PTGS2, TNF, ICAM1	22	43	16792	106.5032	1.59E-06	2.03E-07	1.85 E-07
KEGG_PATHWAY	hsa05144:Malaria	7	31.8181 8	2.41E-09	IL10, IL6, TGFB1, IFNG, IL1B, TNF, ICAM1	20	49	6879	49.13571	2.39E-07	3.41E-08	1.93 E-08

GOTERM_BP_DIRECT	GO:0051092~positive regulation of NF-kappaB transcription factor activity	7	31.81818	1.08E-08	IL6, TGFB1, IL1B, NOD2, TNF, TLR3, ICAM1	22	133	16792	40.17225	1.01E-05	1.12E-06	1.02E-06
KEGG_PATHWAY	hsa05332:Graft-versus-host disease	6	27.27273	2.05E-08	IL6, IFNG, IL1B, FAS, FASLG, TNF	20	33	6879	62.53636	2.03E-06	2.26E-07	1.28E-07
GOTERM_BP_DIRECT	GO:0006955~immune response	9	40.90909	2.23E-08	IL10, IL4, IL6, IL1RN, IFNG, IL1B, FAS, FASLG, TNF	22	421	16792	16.31699	2.07E-05	1.75E-06	1.60E-06
KEGG_PATHWAY	hsa05140:Leishmaniasis	7	31.81818	2.38E-08	IL10, IL4, TGFB1, IFNG, IL1B, PTGS2, TNF	20	71	6879	33.91056	2.36E-06	2.36E-07	1.33E-07
UP_KEYWORDS	Cytokine	7	31.81818	2.77E-08	IL10, IL4, IL6, IFNG, IL1B, FASLG, TNF	22	190	20581	34.46579	2.74E-06	2.74E-06	2.54E-06
KEGG_PATHWAY	hsa05330:Allograft rejection	6	27.27273	3.74E-08	IL10, IL4, IFNG, FAS, FASLG, TNF	20	37	6879	55.77568	3.71E-06	3.37E-07	1.91E-07
GOTERM_CC_DIRECT	GO:0005615~extracellular space	12	54.54545	6.10E-08	IL10, IL4, IL6, IL1RN, TGFB1, IFNG, IL1B, APOA1, FASLG, TNF, ELANE, ICAM1	22	1347	18224	7.379632	4.88E-06	4.45E-06	4.01E-06
GOTERM_BP_DIRECT	GO:0045944~positive regulation of transcription from RNA polymerase II promoter	11	50	8.64E-08	IL10, IL4, IL6, TGFB1, IFNG, IL1B, FADD, NOD2, ASCL1, TNF, TLR3	22	981	16792	8.558614	8.02E-05	5.35E-06	4.88E-06

GOTERM_CC_DIRECT	GO:0009897~external side of plasma membrane	7	31.81818	1.11E-07	IL4, IL6, IFNG, FAS, FASLG, TNF, ICAM1	22	213	18224	27.22322	8.91E-06	4.45E-06	4.01E-06
KEGG_PATHWAY	hsa04060:Cytokine-cytokine receptor interaction	9	40.90909	1.17E-07	IL10, IL4, IL6, TGFB1, IFNG, IL1B, FAS, FASLG, TNF	20	243	6879	12.73889	1.16E-05	8.89E-07	5.03E-07
KEGG_PATHWAY	hsa05164:Influenza A	8	36.36364	2.29E-07	IL6, IFNG, IL1B, FAS, FASLG, TNF, TLR3, ICAM1	20	174	6879	15.81379	2.27E-05	1.62E-06	9.16E-07
GOTERM_BP_DIRECT	GO:0006954~inflammatory response	8	36.36364	2.50E-07	IL10, IL6, TGFB1, IL1B, FAS, PTGS2, TNF, TLR3	22	379	16792	16.1113	2.32E-04	1.34E-05	1.23E-05
KEGG_PATHWAY	hsa05146:Amoebiasis	7	31.81818	2.68E-07	IL10, IL6, TGFB1, IFNG, IL1B, CASP3, TNF	20	106	6879	22.71368	2.65E-05	1.77E-06	1.00E-06
GOTERM_BP_DIRECT	GO:0051044~positive regulation of membrane protein ectodomain proteolysis	4	18.18182	7.60E-07	IFNG, IL1B, TNF, ADRA2A	22	15	16792	203.5394	7.05E-04	3.48E-05	3.17E-05
GOTERM_BP_DIRECT	GO:0071407~cellular response to organic cyclic compound	5	22.72727	7.87E-07	TGFB1, IL1B, CASP3, NOD2, TNF	22	59	16792	64.68413	7.30E-04	3.48E-05	3.17E-05
UP_KEYWORDS	Secreted	12	54.54545	8.33E-07	IL10, IL4, IL6, IL1RN, TGFB1, IFNG, IL1B, KDR, FAS, APOA1, FASLG, TNF	22	1965	20581	5.712977	8.25E-05	4.12E-05	3.83E-05

GOTERM_BP_DIRECT	GO:0044130~negative regulation of growth of symbiont in host	4	18.18182	9.34E-07	IL10, IFNG, TNF, ELANE	22	16	16792	190.8182	8.67E-04	3.94E-05	3.60E-05
GOTERM_BP_DIRECT	GO:0042832~defense response to protozoan	4	18.18182	1.61E-06	IL10, IL4, IL6, IFNG	22	19	16792	160.689	0.001495	6.51E-05	5.94E-05
GOTERM_BP_DIRECT	GO:0070374~positive regulation of ERK1 and ERK2 cascade	6	27.27273	2.06E-06	IL6, TGFB1, KDR, NOD2, TNF, ICAM1	22	175	16792	26.16935	0.001913	7.98E-05	7.28E-05
KEGG_PATHWAY	hsa05323:Rheumatoid arthritis	6	27.27273	3.09E-06	IL6, TGFB1, IFNG, IL1B, TNF, ICAM1	20	88	6879	23.45114	3.05E-04	1.80E-05	1.02E-05
GOTERM_CC_DIRECT	GO:0005576~extracellular region	11	50	4.00E-06	IL10, IL4, IL6, TGFB1, IFNG, IL1B, KDR, APOA1, FASLG, TNF, ELANE	22	1610	18224	5.659627	3.20E-04	6.40E-05	5.76E-05
KEGG_PATHWAY	hsa04940:Type I diabetes mellitus	5	22.72727	4.35E-06	IFNG, IL1B, FAS, FASLG, TNF	20	42	6879	40.94643	4.31E-04	2.40E-05	1.35E-05
GOTERM_BP_DIRECT	GO:0060559~positive regulation of calcidiol 1-monooxygenase activity	3	13.63636	4.47E-06	IFNG, IL1B, TNF	22	3	16792	763.2727	0.004135	1.59E-04	1.45E-04
GOTERM_BP_DIRECT	GO:0051091~positive regulation of sequence-specific DNA binding transcription factor activity	5	22.72727	7.96E-06	IL10, IL4, IL6, IL1B, TNF	22	105	16792	36.34632	0.007359	2.64E-04	2.41E-04

GOTERM_BP_DIRECT	GO:0071677~positive regulation of mononuclear cell migration	3	13.63636	8.92E-06	IL4, TGFB1, TNF	22	4	16792	572.4545	0.008247	2.78E-04	2.53E-04
BIOCARTA	h_inflamPathway:Cytokines and Inflammatory Response	6	27.27273	9.22E-06	IL10, IL4, IL6, TGFB1, IFNG, TNF	19	29	1625	17.6951	7.47E-04	7.47E-04	6.46E-04
GOTERM_BP_DIRECT	GO:0071222~cellular response to lipopolysaccharide	5	22.72727	1.07E-05	IL10, IL6, IFNG, TNF, ICAM1	22	113	16792	33.77313	0.009836	3.19E-04	2.91E-04
GOTERM_BP_DIRECT	GO:0010628~positive regulation of gene expression	6	27.27273	1.48E-05	IL6, TGFB1, IFNG, IL1B, TNF, TLR3	22	262	16792	17.47953	0.013605	4.06E-04	3.70E-04
KEGG_PATHWAY	hsa04650:Natural killer cell mediated cytotoxicity	6	27.27273	1.54E-05	IFNG, CASP3, FAS, FASLG, TNF, ICAM1	20	122	6879	16.91557	0.001524	8.03E-05	4.54E-05
KEGG_PATHWAY	hsa05162:Measles	6	27.27273	2.34E-05	IL4, IL6, IFNG, IL1B, FAS, FASLG	20	133	6879	15.51654	0.002317	1.16E-04	6.56E-05
BBID	58.(CD40L)_immnosurveillance	6	27.27273	2.44E-05	IL10, IL6, IFNG, FAS, TNF, ICAM1	12	16	388	12.125	0.001245	6.11E-04	2.93E-04
BBID	97.Immune_injury_MS-lesions_MS_antigen	6	27.27273	2.44E-05	IL10, IL4, IL6, IFNG, FAS, TNF	12	16	388	12.125	0.001245	6.11E-04	2.93E-04

INTERPRO	IPR012351:Four-helical cytokine, core	4	18.1818 2	2.51E-05	IL10, IL4, IL6, IFNG	22	51	18559	66.16399	0.002 157	0.0012 83	0.00 120 9
GOTERM_B P_DIRECT	GO:0042742~defense response to bacterium	5	22.7 272 7	2.85E-05	IL10, IFNG, NOD2, ELANE, TLR3	22	145	16792	26.31975	0.026 08	6.95E-04	6.35 E-04
INTERPRO	IPR009079:Four-helical cytokine-like, core	4	18.1 818 2	2.98E-05	IL10, IL4, IL6, IFNG	22	54	18559	62.48822	0.002 563	0.0012 83	0.00 120 9
GOTERM_B P_DIRECT	GO:0045348~positive regulation of MHC class II biosynthetic process	3	13.6 363 6	4.15E-05	IL10, IL4, IFNG	22	8	16792	286.2273	0.037 799	9.63E-04	8.79 E-04
KEGG_PAT HWAY	hsa05133:Pertussis	5	22.7 272 7	4.46E-05	IL10, IL6, IL1B, CASP3, TNF	20	75	6879	22.93	0.004 409	2.10E-04	1.19 E-04
GOTERM_B P_DIRECT	GO:0042102~positive regulation of T cell proliferation	4	18.1 818 2	5.51E-05	IL4, IL6, IFNG, IL1B	22	60	16792	50.88485	0.049 848	0.0011 36	0.00 103 7
BBID	56.Macrophage_regulation _of_CD4+T_cells	5	22.7 272 7	6.82E-05	IL10, IL4, IL6, IFNG, TNF	12	10	388	16.16667	0.003 473	0.0011 37	5.46 E-04
BIOCARTA	h_cytokinePathway:Cytoki ne Network	5	22.7 272 7	6.82E-05	IL10, IL4, IL6, IFNG, TNF	19	22	1625	19.4378	0.005 512	0.0027 64	0.00 238 8

GOTERM_BP_DIRECT	GO:0050796~regulation of insulin secretion	4	18.18182	7.67E-05	IFNG, IL1B, TNF, ADRA2A	22	67	16792	45.56852	0.068707	0.001514	0.001382
GOTERM_BP_DIRECT	GO:0034116~positive regulation of heterotypic cell-cell adhesion	3	13.63636	8.14E-05	IL10, IL1B, TNF	22	11	16792	208.1653	0.072737	0.001573	0.001436
BBID	19.Cytokine_microglia	5	22.72727	1.06E-04	IL10, IL6, TGFB1, IFNG, TNF	12	11	388	14.69697	0.005373	0.00132	6.34E-04
GOTERM_BP_DIRECT	GO:0050995~negative regulation of lipid catabolic process	3	13.63636	1.77E-04	IL1B, TNF, ADRA2A	22	16	16792	143.1136	0.151388	0.003218	0.002937
KEGG_PATHWAY	hsa05145:Toxoplasmosis	5	22.72727	1.99E-04	IL10, TGFB1, IFNG, CASP3, TNF	20	110	6879	15.63409	0.019546	7.90E-04	4.47E-04
GOTERM_BP_DIRECT	GO:0008284~positive regulation of cell proliferation	6	27.27273	2.27E-04	IL6, TGFB1, IFNG, KDR, FASLG, ADRA2A	22	466	16792	9.827546	0.189868	0.003899	0.003558
KEGG_PATHWAY	hsa04672:Intestinal immune network for IgA production	4	18.18182	2.68E-04	IL10, IL4, IL6, TGFB1	20	47	6879	29.27234	0.026216	0.001022	5.78E-04
BBID	111.Stress_influences_immunity	4	18.18182	3.26E-04	IL10, IL4, IFNG, TNF	12	6	388	21.55556	0.016485	0.002716	0.001303

BBID	112.StressandCRHinfluence	4	18.18182	3.26E-04	IL10, IL4, IFNG, TNF	12	6	388	21.55556	0.016485	0.002716	0.001303
BIOCARTA	h_il1rPathway:Signal transduction through IL1R	5	22.72727	3.54E-04	IL6, IL1RN, TGFB1, IL1B, TNF	19	33	1625	12.95853	0.028238	0.00716	0.006187
GOTERM_BP_DIRECT	GO:0045893~positive regulation of transcription, DNA-templated	6	27.27273	3.60E-04	IL10, IL4, IL6, TGFB1, IL1B, TNF	22	515	16792	8.892498	0.284234	0.006079	0.005548
KEGG_PATHWAY	hsa05134:Legionellosis	4	18.18182	4.05E-04	IL6, IL1B, CASP3, TNF	20	54	6879	25.47778	0.039351	0.001434	8.11E-04
KEGG_PATHWAY	hsa04621:NOD-like receptor signaling pathway	4	18.18182	4.52E-04	IL6, IL1B, NOD2, TNF	20	56	6879	24.56786	0.043726	0.001541	8.72E-04
GOTERM_BP_DIRECT	GO:0001934~positive regulation of protein phosphorylation	4	18.18182	5.09E-04	TGFB1, IL1B, KDR, TNF	22	127	16792	24.04009	0.376328	0.008353	0.007624
GOTERM_BP_DIRECT	GO:0010575~positive regulation of vascular endothelial growth factor production	3	13.63636	5.13E-04	TGFB1, IL1B, PTGS2	22	27	16792	84.80808	0.3789	0.008353	0.007624
BBID	18.Cytokine_astocytes	5	22.72727	5.41E-04	IL6, TGFB1, IFNG, TNF, ICAM1	12	16	388	10.10417	0.027208	0.003862	0.001854

GOTERM_BP_DIRECT	GO:0000060~protein import into nucleus, translocation	3	13.63636	5.52E-04	TGFB1, IFNG, TNF	22	28	16792	81.77922	0.401018	0.008834	0.008063
GOTERM_BP_DIRECT	GO:0031663~lipopolysaccharide-mediated signaling pathway	3	13.63636	7.22E-04	TGFB1, IL1B, TNF	22	32	16792	71.55682	0.488573	0.011172	0.010197
GOTERM_BP_DIRECT	GO:0043491~protein kinase B signaling	3	13.63636	7.68E-04	TGFB1, IL1B, TNF	22	33	16792	69.38843	0.509971	0.011689	0.010669
GOTERM_MF_DIRECT	GO:0008083~growth factor activity	4	18.18182	0.001016	IL10, IL4, IL6, TGFB1	22	162	16881	18.94613	0.102166	0.017953	0.016598
GOTERM_BP_DIRECT	GO:0045599~negative regulation of fat cell differentiation	3	13.63636	0.001244	IL6, TGFB1, TNF	22	42	16792	54.51948	0.68512	0.018044	0.016469
GOTERM_BP_DIRECT	GO:0008285~negative regulation of cell proliferation	5	22.72727	0.001326	IL10, IL6, TGFB1, IL1B, PTGS2	22	396	16792	9.637282	0.708169	0.018935	0.017282
KEGG_PATHWAY	hsa04640:Hematopoietic cell lineage	4	18.18182	0.001636	IL4, IL6, IL1B, TNF	20	87	6879	15.81379	0.149601	0.004907	0.002775
GOTERM_BP_DIRECT	GO:0000122~negative regulation of transcription from RNA polymerase II promoter	6	27.27273	0.00164	TGFB1, IFNG, FASLG, ASCL1, TNF, ELANE	22	720	16792	6.360606	0.781931	0.022712	0.020729

GOTERM_BP_DIRECT	GO:0006959~humoral immune response	3	13.63636	0.002281	IL6, IFNG, TNF	22	57	16792	40.17225	0.879856	0.031127	0.02841
KEGG_PATHWAY	hsa04660:T cell receptor signaling pathway	4	18.18182	0.002439	IL10, IL4, IFNG, TNF	20	100	6879	13.758	0.214758	0.007102	0.004017
BBID	88.Alternatively_Activated_APC	4	18.18182	0.002484	IL10, IL4, IFNG, TNF	12	11	388	11.75758	0.119145	0.015528	0.007453
GOTERM_BP_DIRECT	GO:0050679~positive regulation of epithelial cell proliferation	3	13.63636	0.002524	IL6, TGFB1, NOD2	22	60	16792	38.16364	0.90416	0.032084	0.029283
KEGG_PATHWAY	hsa05310:Asthma	3	13.63636	0.003003	IL10, IL4, TNF	20	30	6879	34.395	0.25748	0.008493	0.004804
BBID	22.Cytokine-chemokine_CNS	4	18.18182	0.003261	IL10, IL4, IL6, IFNG	12	12	388	10.77778	0.153426	0.016303	0.007825
GOTERM_BP_DIRECT	GO:0033138~positive regulation of peptidyl-serine phosphorylation	3	13.63636	0.003418	IL6, TGFB1, TNF	22	70	16792	32.71169	0.958293	0.04286	0.039119
BBID	20.Cytokine_oligodendrocytes	3	13.63636	0.00426	IL6, TGFB1, IFNG	12	4	388	24.25	0.195646	0.017749	0.00852

BBID	21.Cytokine_neurons	3	13.6363636	0.00426	IL6, TGFB1, IFNG	12	4	388	24.25	0.195646	0.017749	0.00852
KEGG_PATHWAY	hsa05166:HTLV-I infection	5	22.7272727	0.004539	IL6, TGFB1, BAX, TNF, ICAM1	20	254	6879	6.770669	0.362618	0.012145	0.00687
GOTERM_BP_DIRECT	GO:0050731~positive regulation of peptidyl-tyrosine phosphorylation	3	13.6363636	0.004658	IL6, TGFB1, ICAM1	22	82	16792	27.92461	0.986864	0.053362	0.048704
GOTERM_BP_DIRECT	GO:0051897~positive regulation of protein kinase B signaling	3	13.6363636	0.004882	IL6, TGFB1, TNF	22	84	16792	27.25974	0.989341	0.054517	0.049758
BIOCARTA	h_il5Pathway:IL 5 Signaling Pathway	3	13.6363636	0.00495	IL4, IL6, IL1B	19	10	1625	25.65789	0.330989	0.05012	0.043313
KEGG_PATHWAY	hsa04380:Osteoclast differentiation	4	18.1818182	0.005232	TGFB1, IFNG, IL1B, TNF	20	131	6879	10.50229	0.405078	0.013631	0.00771
KEGG_PATHWAY	hsa04068:FoxO signaling pathway	4	18.1818182	0.005573	IL10, IL6, TGFB1, FASLG	20	134	6879	10.26716	0.424955	0.013794	0.007803
KEGG_PATHWAY	hsa05322:Systemic lupus erythematosus	4	18.1818182	0.005573	IL10, IFNG, TNF, ELANE	20	134	6879	10.26716	0.424955	0.013794	0.007803

KEGG_PATHWAY	hsa04630:Jak-STAT signaling pathway	4	18.18182	0.006939	IL10, IL4, IL6, IFNG	20	145	6879	9.488276	0.498116	0.016756	0.009478
UP_KEYWORDS	Growth factor	3	13.63636	0.0078	IL4, IL6, TGFB1	22	131	20581	21.42366	0.539421	0.085805	0.079738
BIOCARTA	h_il10Pathway:IL-10 Anti-inflammatory Signaling Pathway	3	13.63636	0.008413	IL10, IL6, TNF	19	13	1625	19.73684	0.495562	0.075714	0.065432
BIOCARTA	h_granulocytesPathway:Adhesion and Diapedesis of Granulocytes	3	13.63636	0.011177	IFNG, TNF, ICAM1	19	15	1625	17.10526	0.597653	0.082304	0.071127
GOTERM_BP_DIRECT	GO:0010629~negative regulation of gene expression	3	13.63636	0.012537	TGFB1, IFNG, TNF	22	137	16792	16.714	0.999992	0.112007	0.10223
BBID	113.Th1andTh2cells	3	13.63636	0.014225	IL10, IL4, IFNG	12	7	388	13.85714	0.518423	0.050804	0.024386
BIOCARTA	h_LairPathway:Cells and Molecules involved in local acute inflammatory response	3	13.63636	0.014288	IL6, TNF, ICAM1	19	17	1625	15.09288	0.688295	0.096446	0.083348
BIOCARTA	h_tob1Pathway:Role of Tob in T-cell activation	3	13.63636	0.017731	IL4, TGFB1, IFNG	19	19	1625	13.50416	0.765226	0.102589	0.088657

GOTERM_BP_DIRECT	GO:0007568~aging	3	13.63636	0.017831	IL10, IL6, TGFB1	22	165	16792	13.87769	1	0.143885	0.131326
BBID	35.Chemokines_EAE_Autoreactive_T_Cells	3	13.63636	0.018672	IL10, IL4, IFNG	12	8	388	12.125	0.617596	0.05835	0.028008
KEGG_PATHWAY	hsa05410:Hypertrophic cardiomyopathy (HCM)	3	13.63636	0.019158	IL6, TGFB1, TNF	20	78	6879	13.22885	0.852672	0.042149	0.023842
KEGG_PATHWAY	hsa05132:Salmonella infection	3	13.63636	0.021534	IL6, IFNG, IL1B	20	83	6879	12.43193	0.884109	0.046344	0.026215
KEGG_PATHWAY	hsa04350:TGF-beta signaling pathway	3	13.63636	0.022023	TGFB1, IFNG, TNF	20	84	6879	12.28393	0.889707	0.046388	0.02624
BIOCARTA	h_dcPathway:Dendritic cells in regulating TH1 and TH2 Development	3	13.63636	0.023487	IL10, IL4, IFNG	19	22	1625	11.66268	0.854146	0.12683	0.109606
BBID	80.T_cell_Activation	4	18.18182	0.027708	IL10, IL4, IL6, IFNG	12	25	388	5.173333	0.761427	0.081496	0.039118
BIOCARTA	h_nktPathway:Selective expression of chemokine receptors during T-cell polarization	3	13.63636	0.036955	IL4, TGFB1, IFNG	19	28	1625	9.163534	0.952646	0.187087	0.16168

GOTERM_BP_DIRECT	GO:0000165~MAPK cascade	3	13.63636	0.041912	TGFB1, IL1B, TNF	22	262	16792	8.739764	1	0.254213	0.232024
BBID	15.T-cell_polarization-chemokine_receptors	3	13.63636	0.07878	IL4, IL6, IFNG	12	17	388	5.705882	0.984776	0.19695	0.094536

**Annotation Cluster 7**	**Enrichment Score: 3.1473494363499976**											
Category	Term	Count	%	PValue	Genes	List Total	Pop Hits	Pop Total	Fold Enrichment	Bonferroni	Benjamini	FDR
GOTERM_BP_DIRECT	GO:0032757~positive regulation of interleukin-8 production	5	22.72727	2.65E-08	IL1B, FADD, NOD2, TNF, TLR3	22	26	16792	146.7832	2.46E-05	1.89E-06	1.73E-06
GOTERM_BP_DIRECT	GO:0032760~positive regulation of tumor necrosis factor production	4	18.18182	2.64E-05	IFNG, FADD, NOD2, TLR3	22	47	16792	64.95938	0.024186	6.62E-04	6.04E-04
UP_KEYWORDS	Innate immunity	3	13.63636	0.028711	FADD, NOD2, TLR3	22	261	20581	10.75287	0.944087	0.236863	0.220115
UP_KEYWORDS	Immunity	3	13.63636	0.091341	FADD, NOD2, TLR3	22	500	20581	5.613	0.999924	0.53193	0.494319
GOTERM_BP_DIRECT	GO:0045087~innate immune response	3	13.63636	0.09984	FADD, NOD2, TLR3	22	430	16792	5.325159	1	0.503539	0.459588

**Annotation Cluster 8**	**Enrichment Score: 1.0512101876673332**											
Category	Term	Count	%	PValue	Genes	List Total	Pop Hits	Pop Total	Fold Enrichment	Bonferroni	Benjamini	FDR
GOTERM_CC_DIRECT	GO:0005887~integral component of plasma membrane	7	31.81818	0.004256	KDR, FAS, FASLG, TNF, ADRA2A, TLR3, ICAM1	22	1415	18224	4.097912	0.289096	0.034049	0.030644
GOTERM_BP_DIRECT	GO:0033209~tumor necrosis factor-mediated signaling pathway	3	13.63636	0.009423	FAS, FASLG, TNF	22	118	16792	19.40524	0.999847	0.093392	0.08524
BIOCARTA	h_ctlPathway:CTL mediated immune response against target cells	3	13.63636	0.009751	FAS, FASLG, ICAM1	19	14	1625	18.32707	0.547819	0.07898	0.068254
BIOCARTA	h_pmlPathway:Regulation of transcriptional activity by PML	3	13.63636	0.015969	FAS, FASLG, TNF	19	18	1625	14.25439	0.728542	0.099501	0.085988
UP_KEYWORDS	Receptor	6	27.27273	0.022543	IL1RN, KDR, FAS, ADRA2A, TLR3, ICAM1	22	1648	20581	3.405947	0.895368	0.202888	0.188542
GOTERM_BP_DIRECT	GO:0030198~extracellular matrix organization	3	13.63636	0.024607	KDR, TNF, ICAM1	22	196	16792	11.68275	1	0.182684	0.166738
GOTERM_MF_DIRECT	GO:0004888~transmembrane signaling receptor activity	3	13.63636	0.028675	FAS, TLR3, ICAM1	22	214	16881	10.7568	0.954222	0.239891	0.221786

GOTERM_MF_DIRECT	GO:0004872~receptor activity	3	13.63636	0.029421	FAS, TLR3, ICAM1	22	217	16881	10.60809	0.957802	0.239891	0.221786
BIOCARTA	h_keratinocytePathway:Keratinocyte Differentiation	3	13.63636	0.096841	FAS, FASLG, TNF	19	48	1625	5.345395	0.999739	0.389118	0.336275
UP_SEQ_FEATURE	topological domain:Cytoplasmic	7	31.81818	0.138995	KDR, FAS, FASLG, TNF, ADRA2A, TLR3, ICAM1	22	3456	20063	1.84713	1	1	1
UP_SEQ_FEATURE	topological domain:Extracellular	6	27.27273	0.15692	KDR, FAS, FASLG, TNF, ADRA2A, ICAM1	22	2787	20063	1.963304	1	1	1
UP_KEYWORDS	Cell membrane	6	27.27273	0.213975	KDR, FAS, FASLG, NOD2, TNF, ADRA2A	22	3175	20581	1.767874	1	1	0.938776
GOTERM_CC_DIRECT	GO:0016020~membrane	5	22.72727	0.242885	FAS, BAX, TNF, TLR3, ICAM1	22	2200	18224	1.882645	1	0.89032	0.801288
UP_SEQ_FEATURE	transmembrane region	8	36.36364	0.263426	KDR, FAS, BAX, FASLG, TNF, ADRA2A, TLR3, ICAM1	22	5056	20063	1.442966	1	1	1
UP_KEYWORDS	Membrane	10	45.45455	0.343247	KDR, FAS, BAX, FASLG, NOD2, PTGS2, TNF, ADRA2A, TLR3, ICAM1	22	7494	20581	1.248332	1	1	0.938776

UP_KEYWORDS	Transmembrane helix	8	36.36364	0.344956	KDR, FAS, BAX, FASLG, TNF, ADRA2A, TLR3, ICAM1	22	5634	20581	1.328364	1	1	0.938776
UP_KEYWORDS	Transmembrane	8	36.36364	0.348174	KDR, FAS, BAX, FASLG, TNF, ADRA2A, TLR3, ICAM1	22	5651	20581	1.324367	1	1	0.938776
UP_KEYWORDS	Phosphoprotein	10	45.45455	0.478779	IL6, CASP3, KDR, FAS, APOA1, FADD, TNF, ADRA2A, TLR3, ICAM1	22	8246	20581	1.134489	1	1	0.938776
GOTERM_CC_DIRECT	GO:0016021~integral component of membrane	7	31.81818	0.572373	KDR, FAS, BAX, FASLG, TNF, TLR3, ICAM1	22	5163	18224	1.123096	1	1	0.911392
UP_KEYWORDS	Alternative splicing	7	31.81818	0.990604	IL4, IL1RN, KDR, FAS, BAX, FASLG, TLR3	22	10587	20581	0.618542	1	1	0.990604
