# Supplementary material for: Huoxiang Zhengqi Oral Liquid Attenuates LPS-Induced Acute Lung Injury by Modulating Short-Chain Fatty Acid Levels and TLR4/NF-κB p65 Pathway
Source: Biomed Res Int. 2023 Feb 17;2023:6183551. doi: 10.1155/2023/6183551 (PMC9957650; doi:10.1155/2023/6183551)

Supplementary Material (2): all the data of experimental validation results.

Table S1: Raw data of lung wet/dry ratio (Table S1-1), thymus index (Table S1-2), spleen index (Table S1-3), and lung injury score and colon injury score (Table S1-4).

Table S1-1: Data of wet weight, dry weight, wet weight to dry weight ratio of lung tissues. (n=4)

group	number	lung wet weight（g）	lung dry weight（g）	Lung Wet/Dry ratio
control group	1	2.27	0.62	3.63
control group	2	2.44	0.77	3.16
control group	3	1.86	0.56	3.33
control group	4	1.73	0.50	3.46
LPS group	1	2.61	0.51	5.10
LPS group	2	1.77	0.30	5.81
LPS group	3	2.47	0.59	4.21
LPS group	4	0.69	0.15	4.65
LPS + DEX group	1	2.91	0.67	4.35
LPS + DEX group	2	2.41	0.49	4.87
LPS + DEX group	3	3.42	0.87	3.94
LPS + DEX group	4	2.36	0.49	4.81
LPS + HZOL-L group	1	2.28	0.63	3.62
LPS + HZOL-L group	2	2.26	0.59	3.85
LPS + HZOL-L group	3	2.27	0.71	3.20
LPS + HZOL-L group	4	2.12	0.59	3.59
LPS + HZOL-M group	1	2.23	0.57	3.89
LPS + HZOL-M group	2	2.37	0.64	3.69
LPS + HZOL-M group	3	2.97	0.78	3.80
LPS + HZOL-M group	4	2.49	0.73	3.43
LPS + HZOL-H group	1	2.30	0.71	3.22
LPS + HZOL-H group	2	2.20	0.72	3.06
LPS + HZOL-H group	3	2.49	0.72	3.47
LPS + HZOL-H group	4	0.55	0.14	4.01

Table S1-2: Data of thymus weight, body weight, thymus index. (n=8)

group	number	thymus weight（mg）	body weight（g）	thymus index
control group	1	640	427	1.50
control group	2	128	403	0.32
control group	3	667	392	1.70
control group	4	399	395	1.01
control group	5	421	324	1.30

control group	6	465	418	1.11
control group	7	630	376	1.67
control group	8	595	403	1.48
LPS group	1	855	343	2.49
LPS group	2	769	405	1.90
LPS group	3	951	379	2.51
LPS group	4	934	338	2.76
LPS group	5	690	376	1.84
LPS group	6	722	391	1.85
LPS group	7	734	400	1.83
LPS group	8	397	405	0.98
LPS + DEX group	1	645	385	1.68
LPS + DEX group	2	766	361	2.12
LPS + DEX group	3	768	370	2.07
LPS + DEX group	4	605	358	1.69
LPS + DEX group	5	577	365	1.58
LPS + DEX group	6	586	381	1.54
LPS + DEX group	7	728	402	1.81
LPS + DEX group	8	758	375	2.02
LPS + HZOL-L group	1	667	400	1.67
LPS + HZOL-L group	2	556	380	1.46
LPS + HZOL-L group	3	433	366	1.18
LPS + HZOL-L group	4	502	381	1.32
LPS + HZOL-L group	5	364	393	0.92
LPS + HZOL-L group	6	239	381	0.63
LPS + HZOL-L group	7	699	390	1.79
LPS + HZOL-L group	8	506	343	1.47
LPS + HZOL-M group	1	674	418	1.61
LPS + HZOL-M group	2	503	364	1.38
LPS + HZOL-M group	3	563	388	1.45
LPS + HZOL-M group	4	495	404	1.22
LPS + HZOL-M group	5	546	388	1.41
LPS + HZOL-M group	6	508	389	1.31
LPS + HZOL-M group	7	573	367	1.56
LPS + HZOL-M group	8	532	399	1.33
LPS + HZOL-H group	1	438	350	1.25
LPS + HZOL-H group	2	496	386	1.29
LPS + HZOL-H group	3	771	360	2.14
LPS + HZOL-H group	4	586	382	1.54
LPS + HZOL-H group	5	385	418	0.92
LPS + HZOL-H group	6	569	372	1.53
LPS + HZOL-H group	7	435	384	1.13
LPS + HZOL-H group	8	524	418	1.25

Table S1-3: Data of spleen weight, body weight, spleen index. (n=8)				
**group**	**number**	**spleen weight （mg）**	**body weight （g）**	**spleen index**
control group	1	947	427	2.22
control group	2	747	403	1.85
control group	3	823	392	2.10
control group	4	742	395	1.88
control group	5	1095	324	3.38
control group	6	862	418	2.06
control group	7	1142	376	3.04
control group	8	945	403	2.35
LPS group	1	1043	343	3.04
LPS group	2	928	405	2.29
LPS group	3	1149	379	3.03
LPS group	4	918	338	2.72
LPS group	5	1075	376	2.86
LPS group	6	1185	391	3.03
LPS group	7	968	400	2.42
LPS group	8	998	405	2.46
LPS + DEX group	1	890	385	2.31
LPS + DEX group	2	965	361	2.68
LPS + DEX group	3	1059	370	2.86
LPS + DEX group	4	1000	358	2.80
LPS + DEX group	5	730	365	2.00
LPS + DEX group	6	888	381	2.33
LPS + DEX group	7	900	402	2.24
LPS + DEX group	8	822	375	2.19
LPS + HZOL-L group	1	1050	400	2.62
LPS + HZOL-L group	2	831	380	2.19
LPS + HZOL-L group	3	811	366	2.21
LPS + HZOL-L group	4	898	381	2.36
LPS + HZOL-L group	5	1029	393	2.62
LPS + HZOL-L group	6	933	381	2.45
LPS + HZOL-L group	7	865	390	2.22
LPS + HZOL-L group	8	801	343	2.33
LPS + HZOL-M group	1	1135	418	2.71
LPS + HZOL-M group	2	854	364	2.35
LPS + HZOL-M group	3	826	388	2.13
LPS + HZOL-M group	4	838	404	2.07
LPS + HZOL-M group	5	971	388	2.50
LPS + HZOL-M group	6	1022	389	2.63
LPS + HZOL-M group	7	939	367	2.56
LPS + HZOL-M group	8	825	399	2.07

LPS + HZOL-H group	1	664	350	1.89
LPS + HZOL-H group	2	794	386	2.06
LPS + HZOL-H group	3	553	360	1.54
LPS + HZOL-H group	4	993	382	2.60
LPS + HZOL-H group	5	828	418	1.98
LPS + HZOL-H group	6	743	372	2.00
LPS + HZOL-H group	7	894	384	2.33
LPS + HZOL-H group	8	979	418	2.34

Table S1-4: Data of lung injury score and colon injury score. (n=3)

**group**	**number**	**Lung injury score**	**Colon injury score**
control group	1	1	1
control group	2	2	1
control group	3	1	0
LPS group	1	8	6
LPS group	2	7	7
LPS group	3	7	6
LPS + DEX group	1	5	3
LPS + DEX group	2	3	1
LPS + DEX group	3	4	1
LPS + HZOL-L group	1	5	4
LPS + HZOL-L group	2	3	3
LPS + HZOL-L group	3	5	4
LPS + HZOL-M group	1	3	3
LPS + HZOL-M group	2	3	1
LPS + HZOL-M group	3	5	1
LPS + HZOL-H group	1	3	1
LPS + HZOL-H group	2	3	2
LPS + HZOL-H group	3	4	1

Figure S1: Original images of H&E staining of lung tissues for three repeat in each group.

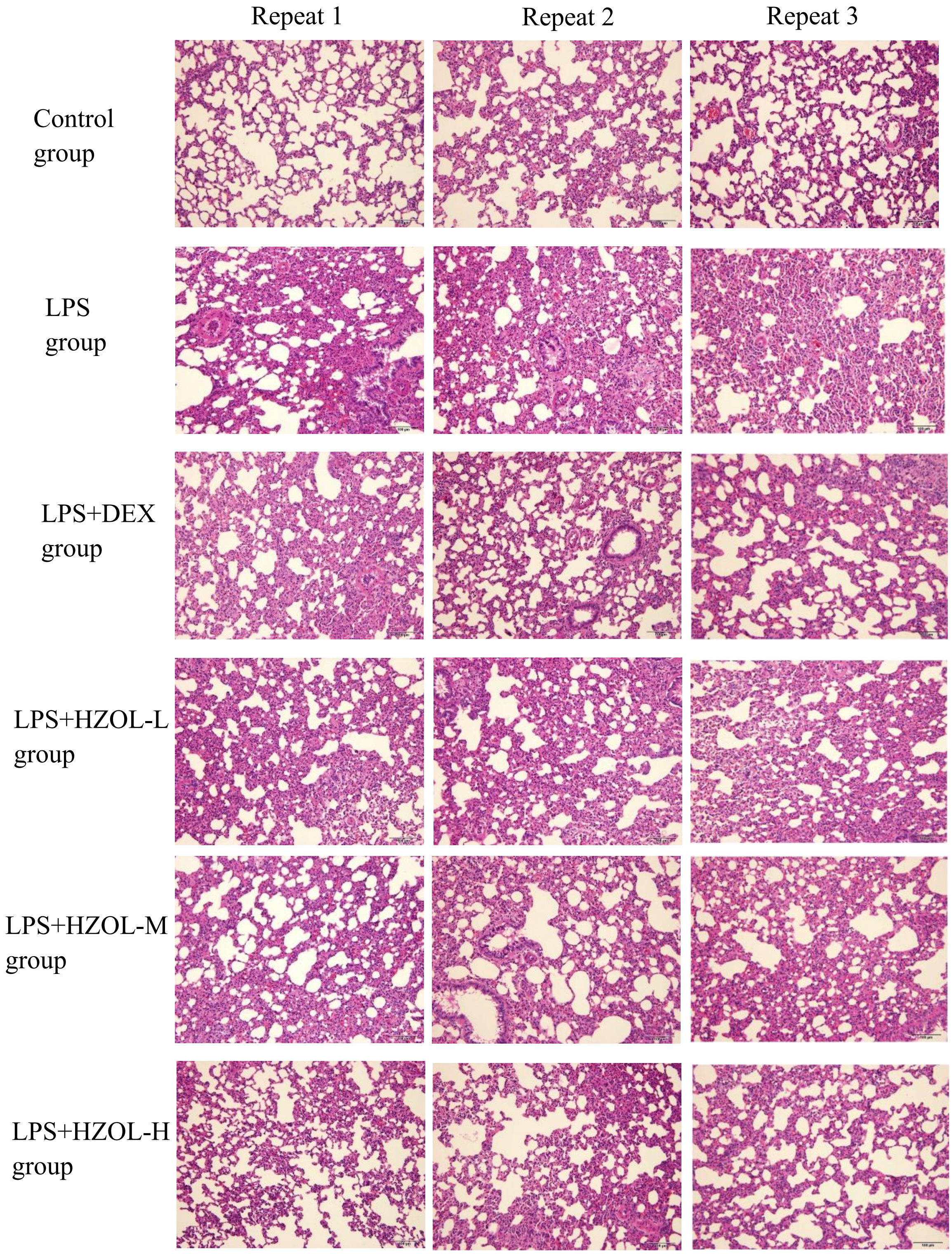

Table S2: Methodological survey data and sample content determination original data of short chain fatty acid by GC-MS method (from Table S2-1 to Table S2-8).

Table S2-1: Material information of short-chain fatty acids.

Number	Name	CAS
1	Acetic acid	64-19-7
2	Propionic acid	1979/9/4
3	Isobutyric acid	79-31-2
4	Butyric acid	107-92-6
5	Isovaleric acid	503-74-2
6	Valeric acid	109-52-4
7	Caproic acid	142-62-1

Table S2-2: Linear regression equation, precision, reproducibility and limit of quantification of 7 short chain fatty acid standards.

Number	Acid	Retention time （min）	Quantitative ion	Linear equation	Coefficient of association （r）	Linearity range (μg/mL)	Within-day precisionRSD (%)	Day to day precisionRSD (%)	Repeatability	Limit of quantitation (μg/mL)
	Name								RSD (%)	
1	Acetic acid	4.57	60	y=0.0064x + 2e-04	0.9922	0.1-100	1.85	11.73	5.44	0.1
2	Propionic acid	5.63	74	y=0.0112x + 2e-04	0.9925	0.1-500	1.73	5.28	4.72	0.1
3	Isobutyric acid	6.03	73	y=0.0179x - 1e-04	0.9965	0.02-500	1.31	4.76	5.65	0.02

4	Butyric acid	6.92	60	y=0.0377x + 2e-04	0.9955	0.02-500	1.57	6.87	5.51	0.02
5	Isovaleric acid	7.57	60	y=0.0451x + 2e-04	0.9977	0.02-500	1.35	6.63	7.59	0.02
6	Valeric acid	8.7	60	y=0.0432x - 3e-04	0.9961	0.02-500	1.3	5.66	7.68	0.02
7	Caproic acid	10.19	60	y=0.0397x + 8e-04	0.9935	0.02-500	1	7.71	9.53	0.02

Table S2-3: Recovery results.

	Acetic acid	Propionic acid	Isobutyric acid	Butyric acid	Isovaleric acid	Valeric acid	Caproic acid
LQC（μg/mL）	1	1	1	1	1	1	1
Recovery (%)	90.34%	94.55%	107.07%	96.35%	92.82%	99.25%	85.22%
MQC（μg/mL）	25	25	25	25	25	25	25
Recovery (%)	97.25%	87.81%	86.17%	85.56%	85.50%	88.16%	96.97%
HQC（μg/mL）	100	100	100	100	100	100	100
Recovery (%)	106.31%	87.53%	85.20%	86.38%	85.80%	89.19%	96.76%

Table S2-4: Stability of 7 kinds of short chain fatty acids in QC samples.

Number	Acid name	Stability RSD（%）
1	Acetic acid	2.04
2	Propionic acid	1.56
3	Isobutyric acid	1.24
4	Butyric acid	1.08
5	Isovaleric acid	0.78

6	Valeric acid	0.95
7	Caproic acid	1.77

Table S2-5: QC quality control.

	IS	Acetic.acid	Ratio.of.Acetic.acid	Mean.of.Acetic.acid	SD.of.Acetic.acid	RSD....of.Acetic.acid
QC1	227616665	83325808	0.3661	0.3630	0.0074	2.0366
QC2	242896237	91272437	0.3758			
QC3	212978443	78038797	0.3664			
QC4	227955380	83428471	0.3660			
QC5	239944648	84699775	0.3530			
QC6	230911010	83210298	0.3604			
QC7	214196153	75807494	0.3539			
QC8	209423005	75972472	0.3628			
	**IS**	**Propionic.acid**	**Ratio.of.Propionic.acid**	**Mean.of.Propionic.acid**	**SD.of.Propionic.acid**	**RSD....of.Propionic.acid**
QC1	227616665	54355400	0.2388	0.2394	0.0037	1.5640
QC2	242896237	59198343	0.2437			
QC3	212978443	51633222	0.2424			
QC4	227955380	54945465	0.2410			
QC5	239944648	55953674	0.2332			
QC6	230911010	55096212	0.2386			
QC7	214196153	50374306	0.2352			
QC8	209423005	50809135	0.2426			
	**IS**	**Isobutyric.acid**	**Ratio.of.Isobutyric.acid**	**Mean.of.Isobutyric.acid**	**SD.of.Isobutyric.acid**	**RSD....of.Isobutyric.acid**
QC1	227616665	8533102	0.0375	0.0375	0.0005	1.2362
QC2	242896237	9212202	0.0379			

QC3	212978443	8023863	0.0377			
QC4	227955380	8598413	0.0377			
QC5	239944648	8850804	0.0369			
QC6	230911010	8664188	0.0375			
QC7	214196153	7854029	0.0367			
QC8	209423005	7941439	0.0379			
	**IS**	**Butyric.acid**	**Ratio.of.Butyric.acid**	**Mean.of.Butyric.acid**	**SD.of.Butyric.acid**	**RSD....of.Butyric.acid**
QC1	227616665	104544395	0.4593	0.4629	0.0050	1.0828
QC2	242896237	113509140	0.4673			
QC3	212978443	98340117	0.4617			
QC4	227955380	106723630	0.4682			
QC5	239944648	109479463	0.4563			
QC6	230911010	107090360	0.4638			
QC7	214196153	97945773	0.4573			
QC8	209423005	98229323	0.4690			
	**IS**	**Isovaleric.acid**	**Ratio.of.Isovaleric.acid**	**Mean.of.Isovaleric.acid**	**SD.of.Isovaleric.acid**	**RSD....of.Isovaleric.acid**
QC1	227616665	23180494	0.1018	0.1018	0.0008	0.7777
QC2	242896237	24834593	0.1022			
QC3	212978443	21884543	0.1028			
QC4	227955380	23191402	0.1017			
QC5	239944648	24141896	0.1006			
QC6	230911010	23489420	0.1017			
QC7	214196153	21600438	0.1008			
QC8	209423005	21523653	0.1028			
	**IS**	**Valeric.acid**	**Ratio.of.Valeric.acid**	**Mean.of.Valeric.acid**	**SD.of.Valeric.acid**	**RSD....of.Valeric.acid**

QC1	227616665	28602031	0.1257	0.1252	0.0012	0.9453
QC2	242896237	30518743	0.1256			
QC3	212978443	26651014	0.1251			
QC4	227955380	28522648	0.1251			
QC5	239944648	29650054	0.1236			
QC6	230911010	28949245	0.1254			
QC7	214196153	26504780	0.1237			
QC8	209423005	26667560	0.1273			
	**IS**	**Caproic.acid**	**Ratio.of.Caproic.acid**	**Mean.of.Caproic.acid**	**SD.of.Caproic.acid**	**RSD....of.Caproic.acid**
QC1	227616665	2307813	0.0101	0.0101	0.0002	1.7659
QC2	242896237	2436356	0.0100			
QC3	212978443	2163445	0.0102			
QC4	227955380	2325992	0.0102			
QC5	239944648	2477646	0.0103			
QC6	230911010	2352569	0.0102			
QC7	214196153	2116659	0.0099			
QC8	209423005	2050222	0.0098			

Table S2-6: Peak area data of SCFAs.

Sampling amount (mg)	Computer code	The sample name	IS	Acetic acid	Propionic acid	Isobutyric acid	Butyric acid	Isovaleric acid	Valeric acid	Caproic acid
49.8	P201204440	control-1	210120626	116154510	67595619	14536986	136720817	32061526	41082509	1448884
49.7	P201204444	control-2	202035551	96080590	90504558	14388333	145233304	39059302	46424281	1705702

49.6	P201204442	control-3	201755147	83748333	53639406	8340602	119551982	20367126	35805328	1597246
50.4	P201204443	LPS-1	200238125	23131698	8456965	700895	20792179	1813960	2504776	1011067
50	P201204441	LPS-2	200330394	35640382	16182794	3420593	28083996	7868085	6148217	1793427
49.6	P201204445	LPS-3	194761709	22616357	12228521	2679941	21577652	6313475	5773708	1126951
50.3	P201204818	LPS+DEX-1	201462480	67247454	30777683	2167094	42517239	4402718	5775065	1164881
49.3	P201204819	LPS+DEX-2	211855880	132092137	88443138	21374925	207986823	56496869	47919612	6414731
49.8	P201204820	LPS+DEX-3	202504352	82019227	63766231	8644925	109395166	22928343	26143891	1442541
49.8	P201204446	LPS+HZOL-H	238884821	154418379	126765602	17140318	220747165	44977270	61128736	1776050
50.1	P201204447	LPS+HZOL-H	215779912	129107718	93418400	9085894	165915538	20562266	42287525	2106635
49.1	P201204448	LPS+HZOL-H	197382772	54555104	33964390	6351772	48863803	15348803	16147548	2301761

Table S2-7: Calculation of SCFAs content.

			Acetic acid	Propionic acid	Isobutyric acid	Butyric acid	Isovaleric acid	Valeric acid	Caproic acid	Total SCFAs
**Sampling amount (mg)**	**Computer code**	**The sample name**	**Content (μg/g)**	**Content (μg/g)**	**Content (μg/g)**	**Content (μg/g)**	**Content (μg/g)**	**Content (μg/g)**	**Content (μg/g)**	**Content (μg/g)**
49.8	P201204440	control-1	866.904	288.206	38.862	173.233	33.924	45.51	1.542	1448.181
49.7	P201204444	control-2	747.238	402.202	40.082	191.774	43.081	53.581	1.937	1479.895
49.6	P201204442	control-3	653.508	239.113	23.338	158.392	22.519	41.482	1.807	1140.159
50.4	P201204443	LPS-1	178.759	37.233	1.995	27.272	1.949	2.942	1.062	251.212
50	P201204441	LPS-2	277.669	71.947	9.595	37.132	8.664	7.174	2.053	414.234
49.6	P201204445	LPS-3	182.591	56.332	7.806	29.571	7.201	6.988	1.266	291.755
50.3	P201204818	LPS+DEX-1	518.136	135.412	6.029	55.593	4.773	6.665	1.247	727.855
49.3	P201204819	LPS+DEX-2	987.735	377.851	57.222	264.051	59.925	53.173	7.531	1807.488
49.8	P201204820	LPS+DEX-3	635.079	282.1	24.001	143.814	25.161	30.075	1.599	1141.829
49.8	P201204446	LPS+HZOL-H	1013.764	475.523	40.302	246.043	41.87	59.542	1.678	1878.722

50.1	P201204447	LPS+HZOL-H	932.714	385.598	23.532	203.495	21.043	45.343	2.253	1613.978
49.1	P201204448	LPS+HZOL-H	439.461	156.272	18.364	66.815	17.513	19.355	2.786	720.566

Table S2-8: Raw reading of SCFAs.

Test item name	Sampling amount (mg)	Computer code	The sample name	IS	Peak area data	Ratio	Concentration (μg/mL)	Content (μg/g)	Calibration.curve	R value
Acetic acid	49.8	P201204440	control-1	210120626	116154510	0.553	86.344	866.904	y=0.0064x + 2e-04	r = 0.9922
	49.7	P201204444	control-2	202035551	96080590	0.476	74.275	747.238		
	49.6	P201204442	control-3	201755147	83748333	0.415	64.828	653.508		
	50.4	P201204443	LPS-1	200238125	23131698	0.116	18.019	178.759		
	50	P201204441	LPS-2	200330394	35640382	0.178	27.767	277.669		
	49.6	P201204445	LPS-3	194761709	22616357	0.116	18.113	182.591		
	50.3	P201204818	LPS+DEX-1	201462480	67247454	0.334	52.124	518.136		

	49.3	P201204819	LPS+DEX-2	211855880	132092137	0.623	97.391	987.735		
	49.8	P201204820	LPS+DEX-3	202504352	82019227	0.405	63.254	635.079		
	49.8	P201204446	LPS+HZOL-H	238884821	154418379	0.646	100.971	1013.764		
	50.1	P201204447	LPS+HZOL-H	215779912	129107718	0.598	93.458	932.714		
	49.1	P201204448	LPS+HZOL-H	197382772	54555104	0.276	43.155	439.461		
Propionic acid	49.8	P201204440	control-1	210120626	67595619	0.322	28.705	288.206	y=0.0112x + 2e-04	r = 0.9925
	49.7	P201204444	control-2	202035551	90504558	0.448	39.979	402.202		
	49.6	P201204442	control-3	201755147	53639406	0.266	23.72	239.113		
	50.4	P201204443	LPS-1	200238125	8456965	0.042	3.753	37.233		
	50	P201204441	LPS-2	200330394	16182794	0.081	7.195	71.947		

	49.6	P201204445	LPS-3	194761709	12228521	0.063	5.588	56.332		
	50.3	P201204818	LPS+DEX-1	201462480	30777683	0.153	13.622	135.412		
	49.3	P201204819	LPS+DEX-2	211855880	88443138	0.417	37.256	377.851		
	49.8	P201204820	LPS+DEX-3	202504352	63766231	0.315	28.097	282.1		
	49.8	P201204446	LPS+HZOL-H	238884821	126765602	0.531	47.362	475.523		
	50.1	P201204447	LPS+HZOL-H	215779912	93418400	0.433	38.637	385.598		
	49.1	P201204448	LPS+HZOL-H	197382772	33964390	0.172	15.346	156.272		
Isobutyric acid	49.8	P201204440	control-1	210120626	14536986	0.069	3.871	38.862	y=0.0179x - 1e-04	r = 0.9965
	49.7	P201204444	control-2	202035551	14388333	0.071	3.984	40.082		
	49.6	P201204442	control-3	201755147	8340602	0.041	2.315	23.338		

	50.4	P201204443	LPS-1	200238125	700895	0.004	0.201	1.995		
	50	P201204441	LPS-2	200330394	3420593	0.017	0.959	9.595		
	49.6	P201204445	LPS-3	194761709	2679941	0.014	0.774	7.806		
	50.3	P201204818	LPS+DEX-1	201462480	2167094	0.011	0.607	6.029		
	49.3	P201204819	LPS+DEX-2	211855880	21374925	0.101	5.642	57.222		
	49.8	P201204820	LPS+DEX-3	202504352	8644925	0.043	2.391	24.001		
	49.8	P201204446	LPS+HZOL-H	238884821	17140318	0.072	4.014	40.302		
	50.1	P201204447	LPS+HZOL-H	215779912	9085894	0.042	2.358	23.532		
	49.1	P201204448	LPS+HZOL-H	197382772	6351772	0.032	1.803	18.364		
Butyric acid	49.8	P201204440	control-1	210120626	136720817	0.651	17.254	173.233	y=0.0377x + 2e-04	r = 0.9955

	49.7	P201204444	control-2	202035551	145233304	0.719	19.062	191.774		
	49.6	P201204442	control-3	201755147	119551982	0.593	15.712	158.392		
	50.4	P201204443	LPS-1	200238125	20792179	0.104	2.749	27.272		
	50	P201204441	LPS-2	200330394	28083996	0.14	3.713	37.132		
	49.6	P201204445	LPS-3	194761709	21577652	0.111	2.933	29.571		
	50.3	P201204818	LPS+DEX-1	201462480	42517239	0.211	5.593	55.593		
	49.3	P201204819	LPS+DEX-2	211855880	207986823	0.982	26.035	264.051		
	49.8	P201204820	LPS+DEX-3	202504352	109395166	0.54	14.324	143.814		
	49.8	P201204446	LPS+HZOL-H	238884821	220747165	0.924	24.506	246.043		
	50.1	P201204447	LPS+HZOL-H	215779912	165915538	0.769	20.39	203.495		

	49.1	P201204448	LPS+HZOL-H	197382772	48863803	0.248	6.561	66.815		
Isovaleric acid	49.8	P201204440	control-1	210120626	32061526	0.153	3.379	33.924	y=0.0451x + 2e-04	r = 0.9977
	49.7	P201204444	control-2	202035551	39059302	0.193	4.282	43.081		
	49.6	P201204442	control-3	201755147	20367126	0.101	2.234	22.519		
	50.4	P201204443	LPS-1	200238125	1813960	0.009	0.196	1.949		
	50	P201204441	LPS-2	200330394	7868085	0.039	0.866	8.664		
	49.6	P201204445	LPS-3	194761709	6313475	0.032	0.714	7.201		
	50.3	P201204818	LPS+DEX-1	201462480	4402718	0.022	0.48	4.773		
	49.3	P201204819	LPS+DEX-2	211855880	56496869	0.267	5.909	59.925		
	49.8	P201204820	LPS+DEX-3	202504352	22928343	0.113	2.506	25.161		

	49.8	P201204446	LPS+HZOL-H	238884821	44977270	0.188	4.17	41.87		
	50.1	P201204447	LPS+HZOL-H	215779912	20562266	0.095	2.108	21.043		
	49.1	P201204448	LPS+HZOL-H	197382772	15348803	0.078	1.72	17.513		
Valeric acid	49.8	P201204440	control-1	210120626	41082509	0.196	4.533	45.51	y=0.0432x - 3e-04	r = 0.9961
	49.7	P201204444	control-2	202035551	6148217	0.031	0.717	7.174		
	49.6	P201204442	control-3	201755147	35805328	0.177	4.115	41.482		
	50.4	P201204443	LPS-1	200238125	2504776	0.013	0.297	2.942		
	50	P201204441	LPS-2	200330394	46424281	0.23	5.326	53.581		
	49.6	P201204445	LPS-3	194761709	5773708	0.03	0.693	6.988		
	50.3	P201204818	LPS+DEX-1	201462480	5775065	0.029	0.671	6.665		

	49.3	P201204819	LPS+DEX-2	211855880	47919612	0.226	5.243	53.173		
	49.8	P201204820	LPS+DEX-3	202504352	26143891	0.129	2.995	30.075		
	49.8	P201204446	LPS+HZOL-H	238884821	61128736	0.256	5.93	59.542		
	50.1	P201204447	LPS+HZOL-H	215779912	42287525	0.196	4.543	45.343		
	49.1	P201204448	LPS+HZOL-H	197382772	16147548	0.082	1.901	19.355		
Caproic acid	49.8	P201204440	control-1	210120626	1448884	0.007	0.154	1.542	y=0.0397x + 8e-04	r = 0.9935
	49.7	P201204444	control-2	202035551	1705702	0.008	0.193	1.937		
	49.6	P201204442	control-3	201755147	1597246	0.008	0.179	1.807		
	50.4	P201204443	LPS-1	200238125	1011067	0.005	0.107	1.062		
	50	P201204441	LPS-2	200330394	1793427	0.009	0.205	2.053		

	49.6	P201204445	LPS-3	194761709	1126951	0.006	0.126	1.266		
	50.3	P201204818	LPS+DEX-1	201462480	1164881	0.006	0.125	1.247		
	49.3	P201204819	LPS+DEX-2	211855880	6414731	0.03	0.743	7.531		
	49.8	P201204820	LPS+DEX-3	202504352	1442541	0.007	0.159	1.599		
	49.8	P201204446	LPS+HZOL-H	238884821	1776050	0.007	0.167	1.678		
	50.1	P201204447	LPS+HZOL-H	215779912	2106635	0.01	0.226	2.253		
	49.1	P201204448	LPS+HZOL-H	197382772	2301761	0.012	0.274	2.786		

Figure S2: Total Ion Flow Chromatography of blank (Figure S2-1), mixed standard (Figure S2-2), sample (Figure S2-3), and overlap chromatogram of QC samples (Figure S2-4).

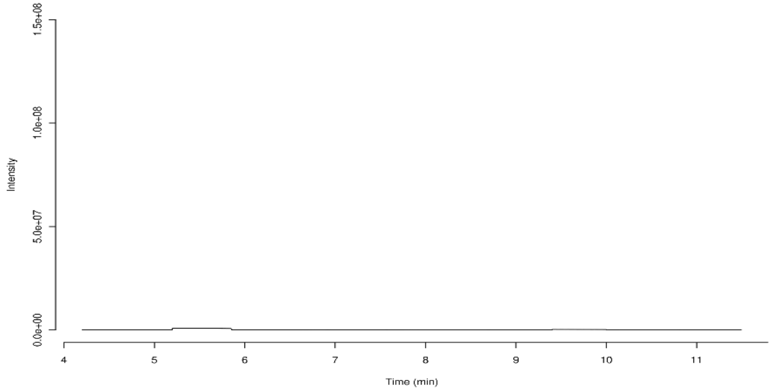

Figure S2-1: Total Ion Flow Chromatography (TIC) of blank.

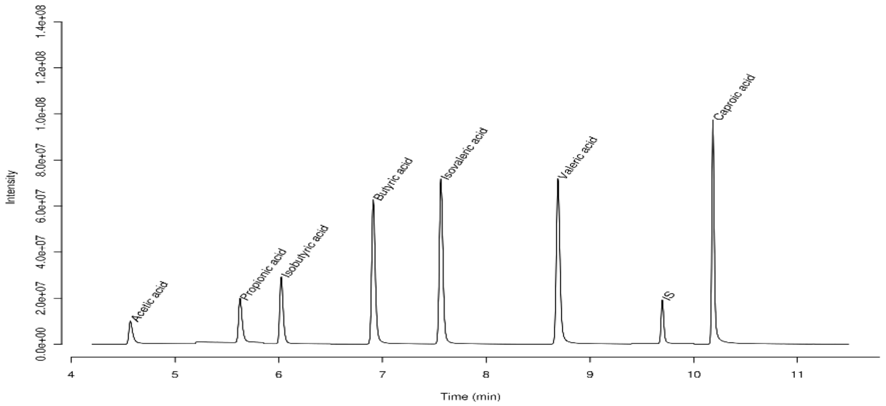

Figure S2-2: Total Ion Flow Chromatography (TIC) of mixed standard.

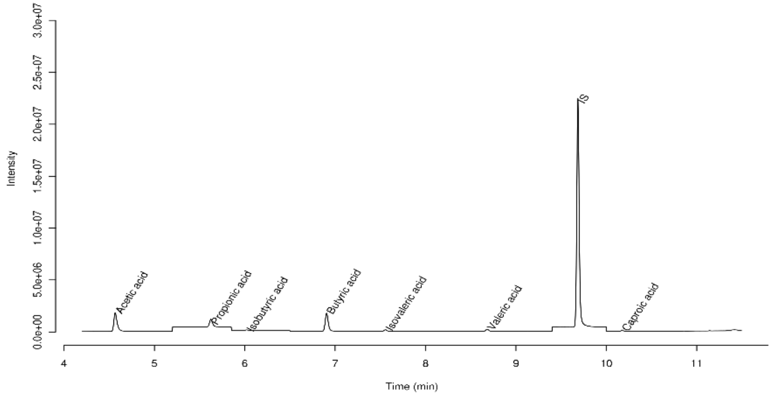

Figure S2-3: Total Ion Flow Chromatography (TIC) of sample.

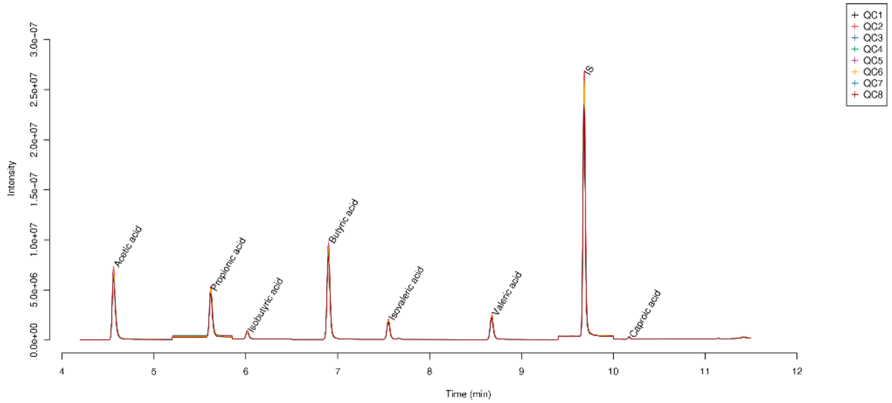

Figure S2-4: Overlap chromatogram of QC samples.

Figure S3: Original images of H&E staining of colon tissues for three repeat in each group.

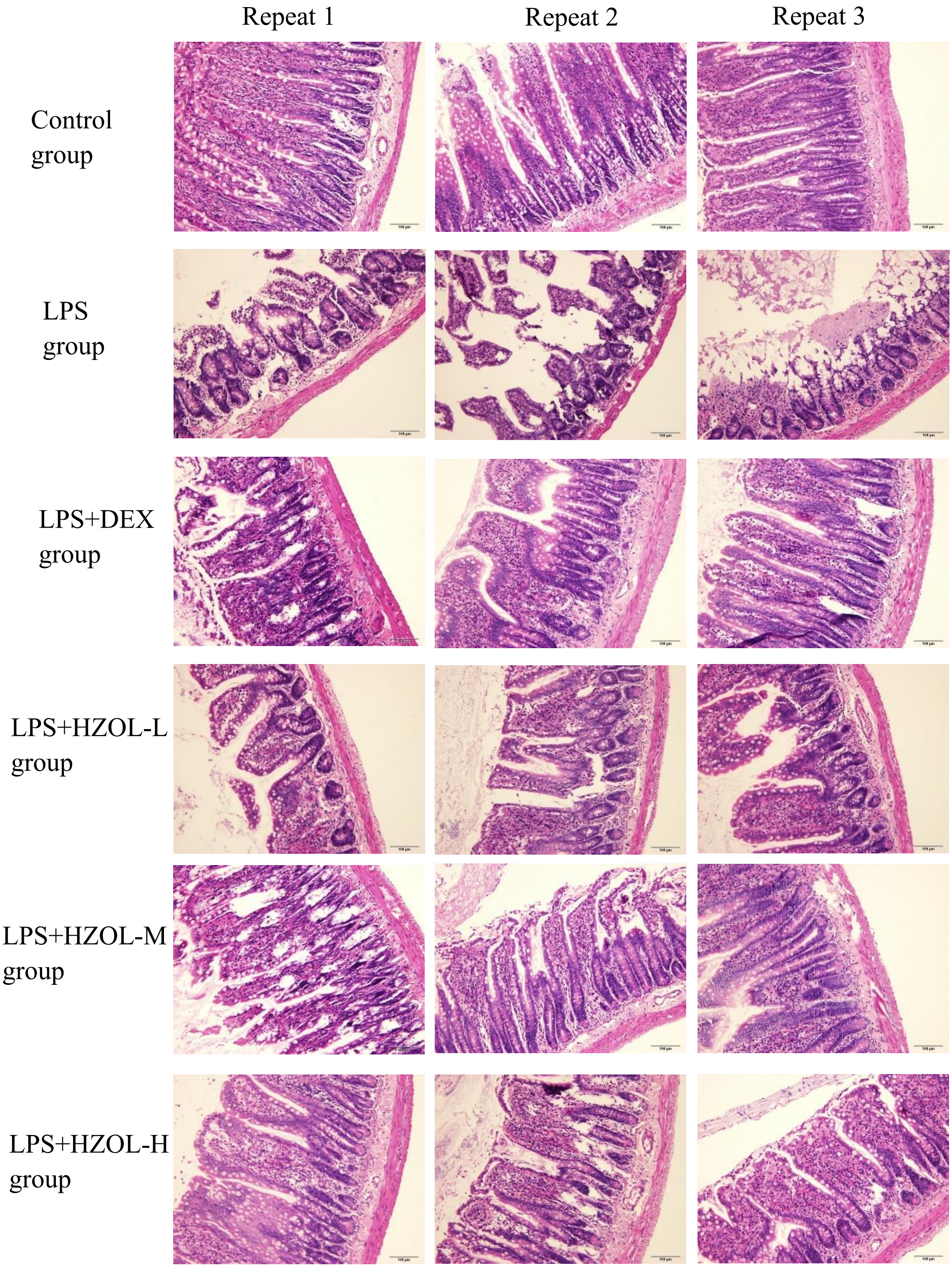

Table S3: Data of WBC, GRAN%, BASO%, EOS%, LYM%, and PLT for eight repeats in each group.

Table S3-1: Data of total white blood cells (WBC).

group	number	white blood cells count (WBC) (×10^9/L)
control group	1	2.00
control group	2	1.60
control group	3	1.20
control group	4	1.00
control group	5	2.40
control group	6	1.20
control group	7	1.10
control group	8	1.10
LPS group	1	4.30
LPS group	2	3.50
LPS group	3	3.20
LPS group	4	4.40
LPS group	5	5.50
LPS group	6	2.80
LPS group	7	4.70
LPS group	8	4.60
LPS + DEX group	1	2.80
LPS + DEX group	2	2.30
LPS + DEX group	3	1.90
LPS + DEX group	4	2.20
LPS + DEX group	5	2.90
LPS + DEX group	6	2.50
LPS + DEX group	7	3.40
LPS + DEX group	8	2.60
LPS + HZOL-L group	1	3.80
LPS + HZOL-L group	2	3.30
LPS + HZOL-L group	3	2.20
LPS + HZOL-L group	4	2.40
LPS + HZOL-L group	5	3.10
LPS + HZOL-L group	6	4.20
LPS + HZOL-L group	7	2.90
LPS + HZOL-L group	8	4.10
LPS + HZOL-M group	1	3.40
LPS + HZOL-M group	2	3.10
LPS + HZOL-M group	3	2.40
LPS + HZOL-M group	4	3.00
LPS + HZOL-M group	5	3.00
LPS + HZOL-M group	6	2.90
LPS + HZOL-M group	7	3.10

LPS + HZOL-M group	8	3.60
LPS + HZOL-H group	1	3.50
LPS + HZOL-H group	2	3.80
LPS + HZOL-H group	3	3.10
LPS + HZOL-H group	4	2.20
LPS + HZOL-H group	5	2.10
LPS + HZOL-H group	6	3.00
LPS + HZOL-H group	7	2.80
LPS + HZOL-H group	8	2.60

Table S3-2: Data of granulocyte concentration (GRAN%).

**group**	**number**	**granulocyte concentration (GRAN%) (%)**
control group	1	22.00
control group	2	22.50
control group	3	30.00
control group	4	28.00
control group	5	23.33
control group	6	32.50
control group	7	29.09
control group	8	24.55
LPS group	1	66.51
LPS group	2	60.57
LPS group	3	74.06
LPS group	4	66.36
LPS group	5	73.27
LPS group	6	68.57
LPS group	7	61.49
LPS group	8	53.91
LPS + DEX group	1	46.07
LPS + DEX group	2	41.30
LPS + DEX group	3	54.74
LPS + DEX group	4	62.27
LPS + DEX group	5	48.97
LPS + DEX group	6	57.20
LPS + DEX group	7	43.53
LPS + DEX group	8	57.69
LPS + HZOL-L group	1	59.21
LPS + HZOL-L group	2	52.73
LPS + HZOL-L group	3	50.91
LPS + HZOL-L group	4	54.58
LPS + HZOL-L group	5	52.90

LPS + HZOL-L group	6	50.71
LPS + HZOL-L group	7	60.00
LPS + HZOL-L group	8	69.02
LPS + HZOL-M group	1	50.59
LPS + HZOL-M group	2	65.48
LPS + HZOL-M group	3	57.50
LPS + HZOL-M group	4	57.67
LPS + HZOL-M group	5	44.00
LPS + HZOL-M group	6	52.07
LPS + HZOL-M group	7	58.71
LPS + HZOL-M group	8	54.17
LPS + HZOL-H group	1	56.29
LPS + HZOL-H group	2	57.37
LPS + HZOL-H group	3	51.61
LPS + HZOL-H group	4	56.82
LPS + HZOL-H group	5	58.10
LPS + HZOL-H group	6	65.67
LPS + HZOL-H group	7	46.43
LPS + HZOL-H group	8	42.31

Table S3-3: Data of basophil concentration (BASO%).

**group**	**number**	**basophil concentration (BASO%) (%)**
control group	1	1.70
control group	2	2.20
control group	3	2.00
control group	4	2.40
control group	5	2.50
control group	6	2.40
control group	7	1.50
control group	8	1.90
LPS group	1	6.10
LPS group	2	5.00
LPS group	3	5.10
LPS group	4	5.60
LPS group	5	4.90
LPS group	6	4.50
LPS group	7	5.20
LPS group	8	6.70
LPS + DEX group	1	1.70
LPS + DEX group	2	2.30
LPS + DEX group	3	2.20
LPS + DEX group	4	1.60

LPS + DEX group	5	2.20
LPS + DEX group	6	2.20
LPS + DEX group	7	1.40
LPS + DEX group	8	2.50
LPS + HZOL-L group	1	4.30
LPS + HZOL-L group	2	5.80
LPS + HZOL-L group	3	4.40
LPS + HZOL-L group	4	4.60
LPS + HZOL-L group	5	2.80
LPS + HZOL-L group	6	4.70
LPS + HZOL-L group	7	2.20
LPS + HZOL-L group	8	6.10
LPS + HZOL-M group	1	3.70
LPS + HZOL-M group	2	4.30
LPS + HZOL-M group	3	5.10
LPS + HZOL-M group	4	4.20
LPS + HZOL-M group	5	3.50
LPS + HZOL-M group	6	5.50
LPS + HZOL-M group	7	4.20
LPS + HZOL-M group	8	3.40
LPS + HZOL-H group	1	2.90
LPS + HZOL-H group	2	3.00
LPS + HZOL-H group	3	2.60
LPS + HZOL-H group	4	2.80
LPS + HZOL-H group	5	3.80
LPS + HZOL-H group	6	2.50
LPS + HZOL-H group	7	2.70
LPS + HZOL-H group	8	4.00

Table S3-4: Data of eosinophil concentration (EOS%).

**group**	**number**	**eosinophil concentration (EOS%) (%)**
control group	1	9.10
control group	2	7.90
control group	3	9.70
control group	4	8.80
control group	5	6.70
control group	6	6.10
control group	7	8.20
control group	8	7.80
LPS group	1	14.60
LPS group	2	15.20
LPS group	3	14.90

LPS group	4	14.80
LPS group	5	16.10
LPS group	6	15.90
LPS group	7	16.00
LPS group	8	16.00
LPS + DEX group	1	9.80
LPS + DEX group	2	9.90
LPS + DEX group	3	10.00
LPS + DEX group	4	10.70
LPS + DEX group	5	9.70
LPS + DEX group	6	9.10
LPS + DEX group	7	8.80
LPS + DEX group	8	8.60
LPS + HZOL-L group	1	14.50
LPS + HZOL-L group	2	14.90
LPS + HZOL-L group	3	14.60
LPS + HZOL-L group	4	12.70
LPS + HZOL-L group	5	15.80
LPS + HZOL-L group	6	15.30
LPS + HZOL-L group	7	12.20
LPS + HZOL-L group	8	14.00
LPS + HZOL-M group	1	13.60
LPS + HZOL-M group	2	15.00
LPS + HZOL-M group	3	15.40
LPS + HZOL-M group	4	13.20
LPS + HZOL-M group	5	12.90
LPS + HZOL-M group	6	14.30
LPS + HZOL-M group	7	13.40
LPS + HZOL-M group	8	13.50
LPS + HZOL-H group	1	13.00
LPS + HZOL-H group	2	11.00
LPS + HZOL-H group	3	13.70
LPS + HZOL-H group	4	9.30
LPS + HZOL-H group	5	11.40
LPS + HZOL-H group	6	12.70
LPS + HZOL-H group	7	9.40
LPS + HZOL-H group	8	12.30

Table S3-5: Data of lymphocyte concentration (LYM%).

**group**	**number**	**lymphocyte concentration (LYM%) (%)**
control group	1	9.10
control group	2	7.90

control group	3	9.70
control group	4	8.80
control group	5	6.70
control group	6	6.10
control group	7	8.20
control group	8	7.80
LPS group	1	14.60
LPS group	2	15.20
LPS group	3	14.90
LPS group	4	14.80
LPS group	5	16.10
LPS group	6	15.90
LPS group	7	16.00
LPS group	8	16.00
LPS + DEX group	1	9.80
LPS + DEX group	2	9.90
LPS + DEX group	3	10.00
LPS + DEX group	4	10.70
LPS + DEX group	5	9.70
LPS + DEX group	6	9.10
LPS + DEX group	7	8.80
LPS + DEX group	8	8.60
LPS + HZOL-L group	1	14.50
LPS + HZOL-L group	2	14.90
LPS + HZOL-L group	3	14.60
LPS + HZOL-L group	4	12.70
LPS + HZOL-L group	5	15.80
LPS + HZOL-L group	6	15.30
LPS + HZOL-L group	7	12.20
LPS + HZOL-L group	8	14.00
LPS + HZOL-M group	1	13.60
LPS + HZOL-M group	2	15.00
LPS + HZOL-M group	3	15.40
LPS + HZOL-M group	4	13.20
LPS + HZOL-M group	5	12.90
LPS + HZOL-M group	6	14.30
LPS + HZOL-M group	7	13.40
LPS + HZOL-M group	8	13.50
LPS + HZOL-H group	1	13.00
LPS + HZOL-H group	2	11.00
LPS + HZOL-H group	3	13.70
LPS + HZOL-H group	4	9.30
LPS + HZOL-H group	5	11.40

LPS + HZOL-H group	6	12.70
LPS + HZOL-H group	7	9.40
LPS + HZOL-H group	8	12.30

Table S3-6: Data of platelet count (PLT).

group	number	platelet count (PLT) (×10^9/L)
control group	1	2.00
control group	2	1.60
control group	3	1.20
control group	4	1.00
control group	5	2.40
control group	6	1.20
control group	7	1.10
control group	8	1.10
LPS group	1	4.30
LPS group	2	3.50
LPS group	3	3.20
LPS group	4	4.40
LPS group	5	5.50
LPS group	6	2.80
LPS group	7	4.70
LPS group	8	4.60
LPS + DEX group	1	2.80
LPS + DEX group	2	2.30
LPS + DEX group	3	1.90
LPS + DEX group	4	2.20
LPS + DEX group	5	2.90
LPS + DEX group	6	2.50
LPS + DEX group	7	3.40
LPS + DEX group	8	2.60
LPS + HZOL-L group	1	3.80
LPS + HZOL-L group	2	3.30
LPS + HZOL-L group	3	2.20
LPS + HZOL-L group	4	2.40
LPS + HZOL-L group	5	3.10
LPS + HZOL-L group	6	4.20
LPS + HZOL-L group	7	2.90
LPS + HZOL-L group	8	4.10
LPS + HZOL-M group	1	3.40
LPS + HZOL-M group	2	3.10
LPS + HZOL-M group	3	2.40
LPS + HZOL-M group	4	3.00
LPS + HZOL-M group	5	3.00

LPS + HZOL-M group	6	2.90
LPS + HZOL-M group	7	3.10
LPS + HZOL-M group	8	3.60
LPS + HZOL-H group	1	3.50
LPS + HZOL-H group	2	3.80
LPS + HZOL-H group	3	3.10
LPS + HZOL-H group	4	2.20
LPS + HZOL-H group	5	2.10
LPS + HZOL-H group	6	3.00
LPS + HZOL-H group	7	2.80
LPS + HZOL-H group	8	2.60

Table S4: Data of IL-6, IL-1β, TNF-α, and IFN-γ in serum and bronchoalveolar lavage fluid for eight repeats in each group. (from Table S4-1 to Table S4-16)

Table S4-1: Standard curve of IL-6 in serum drawing by the help of ELISACalc.exe software.

Groups	Numbers	Absorbance value	Corrected absorbance value	Diluted concentration (pg/mL)
standards sample	1	0.084	0.000	0
standards sample	2	0.104	0.020	40.96
standards sample	3	0.139	0.055	102.4
standards sample	4	0.261	0.177	256
standards sample	5	0.542	0.458	640
standards sample	6	1.041	0.957	1600
standards sample	7	1.588	1.504	4000
standards sample	8	2.020	1.936	10000

Table S4-2: Data of absorbance value and its concentration of IL-6 in serum.

Groups	Numbers	Absorbance value	Diluted concentration (pg/mL)	Actual concentration (pg/mL)
control group	1	0.115	168.45	336.90
control group	2	0.214	296.64	593.29
control group	3	0.186	260.03	520.07
control group	4	0.274	376.63	753.27
control group	5	0.209	290.08	580.15
control group	6	0.215	297.96	595.92
control group	7	0.220	304.54	609.08
control group	8	0.129	186.43	372.87
LPS group	1	0.889	1459.81	2919.63

LPS group	2	0.842	1350.96	2701.93
LPS group	3	0.773	1201.72	2403.43
LPS group	4	0.841	1348.71	2697.43
LPS group	5	0.753	1160.59	2321.19
LPS group	6	0.473	664.35	1328.71
LPS group	7	0.720	1094.67	2189.34
LPS group	8	0.587	850.60	1701.19
LPS + DEX group	1	0.558	801.42	1602.84
LPS + DEX group	2	0.513	727.61	1455.21
LPS + DEX group	3	0.306	420.35	840.70
LPS + DEX group	4	0.350	481.88	963.75
LPS + DEX group	5	0.332	456.50	912.99
LPS + DEX group	6	0.160	226.35	452.69
LPS + DEX group	7	0.343	471.97	943.94
LPS + DEX group	10	0.390	539.41	1078.81
LPS + HZOL-L group	1	0.568	818.23	1636.46
LPS + HZOL-L group	2	0.549	786.42	1572.84
LPS + HZOL-L group	3	0.677	1012.16	2024.33
LPS + HZOL-L group	4	0.712	1079.04	2158.08
LPS + HZOL-L group	5	0.624	915.31	1830.62
LPS + HZOL-L group	6	0.584	845.45	1690.90
LPS + HZOL-L group	7	0.658	976.85	1953.70
LPS + HZOL-L group	8	0.642	947.63	1895.27
LPS + HZOL-M group	1	0.508	719.58	1439.17
LPS + HZOL-M group	2	0.436	607.66	1215.32
LPS + HZOL-M group	3	0.575	830.09	1660.18
LPS + HZOL-M group	4	0.491	692.56	1385.11
LPS + HZOL-M group	5	0.582	842.02	1684.05
LPS + HZOL-M group	6	0.682	1021.57	2043.14
LPS + HZOL-M group	7	0.754	1162.63	2325.26
LPS + HZOL-M group	8	0.563	809.81	1619.61
LPS + HZOL-H group	1	0.235	324.38	648.75
LPS + HZOL-H group	2	0.409	567.31	1134.63
LPS + HZOL-H group	3	0.396	548.18	1096.35
LPS + HZOL-H group	4	0.469	658.14	1316.29
LPS + HZOL-H group	5	0.430	598.62	1197.24
LPS + HZOL-H group	6	0.527	750.26	1500.52
LPS + HZOL-H group	7	0.436	607.66	1215.32
LPS + HZOL-H group	8	0.451	630.44	1260.89

Note 1. The dilution factor of the samples to be tested was 2 times.

Note 2. Diluted concentration was calculated by the help of ELISACalc.exe software according to the standard curve in Table S8-1.

Table S4-3: Standard curve of IL-1β in serum drawing by the help of ELISACalc.exe software.

Groups	Numbers	Absorbance value	Corrected absorbance value	Diluted concentration (pg/mL)
standards sample	1	0.021	0.000	0
standards sample	2	0.035	0.014	31.25
standards sample	3	0.052	0.031	62.5
standards sample	4	0.225	0.204	125
standards sample	5	0.472	0.451	250
standards sample	6	1.107	1.086	500
standards sample	7	1.686	1.665	1000
standards sample	8	2.045	2.024	2000

Table S4-4: Data of absorbance value and its concentration of IL-1β in serum.

Groups	Numbers	Absorbance value	Diluted concentration (pg/mL)	Actual concentration (pg/mL)
control group	1	0.043	56.57	282.84
control group	2	0.116	99.96	499.78
control group	3	0.093	87.76	438.78
control group	4	0.173	127.24	636.18
control group	5	0.112	97.90	489.49
control group	6	0.117	100.47	502.34
control group	7	0.121	102.50	512.49
control group	8	0.052	62.90	314.48
LPS group	1	1.054	493.26	2466.31
LPS group	2	0.980	456.62	2283.10
LPS group	3	0.870	406.13	2030.63
LPS group	4	0.977	455.18	2275.92
LPS group	5	0.837	391.74	1958.71
LPS group	6	0.413	224.24	1121.18
LPS group	7	0.785	369.67	1848.35
LPS group	8	0.578	287.11	1435.54
LPS + DEX group	1	0.536	271.02	1355.09
LPS + DEX group	2	0.470	245.91	1229.55
LPS + DEX group	3	0.206	141.79	708.94
LPS + DEX group	4	0.256	162.75	813.76
LPS + DEX group	5	0.235	154.08	770.39
LPS + DEX group	6	0.073	76.27	381.34
LPS + DEX group	7	0.248	159.47	797.33
LPS + DEX group	10	0.305	182.44	912.21
LPS + HZOL-L group	1	0.603	296.75	1483.77

LPS + HZOL-L group	2	0.522	265.68	1328.40
LPS + HZOL-L group	3	0.545	274.46	1372.28
LPS + HZOL-L group	4	0.707	337.72	1688.60
LPS + HZOL-L group	5	0.803	377.23	1886.17
LPS + HZOL-L group	6	0.610	299.47	1497.33
LPS + HZOL-L group	7	0.704	336.52	1682.58
LPS + HZOL-L group	8	0.663	320.20	1600.99
LPS + HZOL-M group	1	0.463	243.25	1216.26
LPS + HZOL-M group	2	0.364	205.46	1027.29
LPS + HZOL-M group	3	0.561	280.58	1402.90
LPS + HZOL-M group	4	0.438	233.75	1168.77
LPS + HZOL-M group	5	0.571	284.42	1422.08
LPS + HZOL-M group	6	0.726	345.39	1726.95
LPS + HZOL-M group	7	0.839	392.60	1963.02
LPS + HZOL-M group	8	0.543	273.69	1368.45
LPS + HZOL-H group	1	0.135	109.44	547.18
LPS + HZOL-H group	2	0.329	191.88	959.38
LPS + HZOL-H group	3	0.312	185.21	926.03
LPS + HZOL-H group	4	0.408	222.33	1111.64
LPS + HZOL-H group	5	0.355	201.98	1009.91
LPS + HZOL-H group	6	0.490	253.51	1267.53
LPS + HZOL-H group	7	0.364	205.46	1027.29
LPS + HZOL-H group	8	0.383	212.76	1063.82

Note 1. The dilution factor of the samples to be tested was 5 times.

Note 2. Diluted concentration was calculated by the help of ELISACalc.exe software according to the standard curve in Table S8-3.

Table S4-5: Standard curve of TNF-α in serum drawing by the help of ELISACalc.exe software.

Groups	Numbers	Absorbance value	Corrected absorbance value	Diluted concentration (pg/mL)
standards sample	1	0.055	0.000	0
standards sample	2	0.069	0.014	82.3
standards sample	3	0.081	0.026	246.9
standards sample	4	0.116	0.061	740.7
standards sample	5	0.241	0.186	2222
standards sample	6	0.601	0.546	6667
standards sample	7	1.256	1.201	20000
standards sample	8	2.075	2.020	100000

Table S4-6: Data of absorbance value and its concentration of TNF-α in serum.

Groups	Numbers	Absorbance value	Diluted concentration (pg/mL)	Actual concentration (pg/mL)
control group	1	0.271	3174.18	6348.35
control group	2	0.215	2521.36	5042.72
control group	3	0.213	2498.27	4996.54
control group	4	0.172	2027.04	4054.07
control group	5	0.174	2049.95	4099.90
control group	6	0.239	2799.56	5599.12
control group	7	0.139	1649.18	3298.36
control group	8	0.146	1729.35	3458.71
LPS group	1	0.418	4974.35	9948.70
LPS group	2	0.213	2498.27	4996.54
LPS group	3	0.481	5798.53	11597.06
LPS group	4	0.426	5077.00	10154.00
LPS group	5	0.370	4369.62	8739.25
LPS group	6	0.346	4073.92	8147.84
LPS group	7	0.354	4172.03	8344.05
LPS group	8	0.202	2371.46	4742.92
LPS + DEX group	1	0.269	3150.62	6301.24
LPS + DEX group	2	0.189	2222.00	4444.00
LPS + DEX group	3	0.170	2004.13	4008.25
LPS + DEX group	4	0.209	2452.12	4904.23
LPS + DEX group	5	0.183	2153.14	4306.27
LPS + DEX group	6	0.187	2199.04	4398.07
LPS + DEX group	7	0.275	3221.35	6442.69
LPS + DEX group	10	0.159	1878.17	3756.35
LPS + HZOL-L group	1	0.282	3304.09	6608.18
LPS + HZOL-L group	2	0.273	3197.75	6395.50
LPS + HZOL-L group	3	0.217	2544.47	5088.94
LPS + HZOL-L group	4	0.275	3221.35	6442.69
LPS + HZOL-L group	5	0.230	2694.99	5389.98
LPS + HZOL-L group	6	0.211	2475.19	4950.37
LPS + HZOL-L group	7	0.271	3174.18	6348.35
LPS + HZOL-L group	8	0.187	2199.04	4398.07
LPS + HZOL-M group	1	0.317	3721.94	7443.87
LPS + HZOL-M group	2	0.230	2694.99	5389.98
LPS + HZOL-M group	3	0.228	2671.79	5343.59
LPS + HZOL-M group	4	0.137	1626.26	3252.53
LPS + HZOL-M group	5	0.239	2799.56	5599.12
LPS + HZOL-M group	6	0.189	2222.00	4444.00
LPS + HZOL-M group	7	0.275	3221.35	6442.69

LPS + HZOL-M group	8	0.309	3625.79	7251.58
LPS + HZOL-H group	1	0.181	2130.20	4260.39
LPS + HZOL-H group	2	0.159	1878.17	3756.35
LPS + HZOL-H group	3	0.198	2325.43	4650.86
LPS + HZOL-H group	4	0.284	3327.78	6655.56
LPS + HZOL-H group	5	0.228	2671.79	5343.59
LPS + HZOL-H group	6	0.204	2394.49	4788.98
LPS + HZOL-H group	7	0.328	3854.79	7709.59
LPS + HZOL-H group	8	0.163	1923.97	3847.93

Note 1. The dilution factor of the samples to be tested was 2 times.

Note 2. Diluted concentration was calculated by the help of ELISACalc.exe software according to the standard curve in Table S8-5.

Table S4-7: Standard curve IFN-γ in serum drawing by the help of ELISACalc.exe software.

Groups	Numbers	Absorbance value	Corrected absorbance value	Diluted concentration (pg/mL)
standards sample	1	0.035	0.000	0
standards sample	2	0.056	0.021	122.9
standards sample	3	0.101	0.066	307.2
standards sample	4	0.302	0.267	768
standards sample	5	0.658	0.623	1920
standards sample	6	1.292	1.257	4800
standards sample	7	1.892	1.857	12000
standards sample	8	2.405	2.370	30000

Table S4-8: Data of absorbance value and its concentration of IFN-γ in serum.

Groups	Numbers	Absorbance value	Diluted concentration (pg/mL)	Actual concentration (pg/mL)
control group	1	0.513	1523.25	7616.27
control group	2	0.415	1212.26	6061.32
control group	3	0.391	1139.21	5696.03
control group	4	0.335	973.01	4865.03
control group	5	0.339	984.69	4923.45
control group	6	0.457	1342.96	6714.81
control group	7	0.272	792.54	3962.72
control group	8	0.285	829.26	4146.30
LPS group	1	0.753	2389.67	11948.36
LPS group	2	0.411	1200.01	6000.04
LPS group	3	0.848	2784.04	13920.20
LPS group	4	0.765	2437.66	12188.32
LPS group	5	0.678	2100.65	10503.27

LPS group	6	0.639	1957.35	9786.73
LPS group	7	0.652	2004.61	10023.06
LPS group	8	0.391	1139.21	5696.03
LPS + DEX group	1	0.510	1513.41	7567.06
LPS + DEX group	2	0.367	1067.27	5336.33
LPS + DEX group	3	0.331	961.35	4806.76
LPS + DEX group	4	0.403	1175.59	5877.96
LPS + DEX group	5	0.355	1031.70	5158.51
LPS + DEX group	6	0.363	1055.38	5276.91
LPS + DEX group	7	0.521	1549.61	7748.03
LPS + DEX group	10	0.310	900.62	4503.08
LPS + HZOL-L group	1	0.602	1825.48	9127.38
LPS + HZOL-L group	2	0.662	2041.31	10206.55
LPS + HZOL-L group	3	0.567	1704.19	8520.96
LPS + HZOL-L group	4	0.469	1381.00	6904.99
LPS + HZOL-L group	5	0.454	1333.50	6667.51
LPS + HZOL-L group	6	0.510	1513.41	7567.06
LPS + HZOL-L group	7	0.519	1543.00	7715.02
LPS + HZOL-L group	8	0.363	1055.38	5276.91
LPS + HZOL-M group	1	0.591	1787.00	8935.02
LPS + HZOL-M group	2	0.442	1295.86	6479.28
LPS + HZOL-M group	3	0.438	1283.38	6416.88
LPS + HZOL-M group	4	0.268	781.30	3906.49
LPS + HZOL-M group	5	0.457	1342.96	6714.81
LPS + HZOL-M group	6	0.367	1067.27	5336.33
LPS + HZOL-M group	7	0.521	1549.61	7748.03
LPS + HZOL-M group	8	0.577	1738.51	8692.56
LPS + HZOL-H group	1	0.351	1019.91	5099.53
LPS + HZOL-H group	2	0.310	900.62	4503.08
LPS + HZOL-H group	3	0.383	1115.10	5575.52
LPS + HZOL-H group	4	0.535	1596.09	7980.47
LPS + HZOL-H group	5	0.438	1283.38	6416.88
LPS + HZOL-H group	6	0.395	1151.30	5756.51
LPS + HZOL-H group	7	0.608	1846.60	9233.00
LPS + HZOL-H group	8	0.318	923.67	4618.33

Note 1. The dilution factor of the samples to be tested was 5 times.

Note 2. Diluted concentration was calculated by the help of ELISACalc.exe software according to the standard curve in Table S8-7.

Table S4-9: Standard curve of IL-6 in bronchoalveolar lavage fluid drawing by the help of ELISACalc.exe software.

Groups	Numbers	Absorbance value	Corrected absorbance value	Diluted concentration (pg/mL)
standards sample	1	0.058	0.000	0
standards sample	2	0.085	0.027	40.96
standards sample	3	0.109	0.051	102.4
standards sample	4	0.166	0.108	256
standards sample	5	0.302	0.244	640
standards sample	6	0.781	0.723	1600
standards sample	7	1.808	1.750	4000
standards sample	8	2.400	2.342	10000

Table S4-10: Data of absorbance value and its concentration of IL-6 in bronchoalveolar lavage fluid.

Groups	Numbers	Absorbance value	Diluted concentration (pg/mL)	Actual concentration (pg/mL)
control group	1	0.159	348.83	697.66
control group	2	0.188	433.48	866.97
control group	3	0.177	402.81	805.62
control group	4	0.190	438.91	877.81
control group	5	0.156	339.27	678.53
control group	6	0.141	288.19	576.38
control group	7	0.141	288.19	576.38
control group	8	0.135	265.84	531.68
LPS group	1	0.822	1622.95	3245.90
LPS group	2	0.718	1442.93	2885.87
LPS group	3	0.819	1617.70	3235.41
LPS group	4	0.688	1391.50	2783.00
LPS group	5	0.462	1000.47	2000.95
LPS group	6	0.642	1312.78	2625.57
LPS group	7	0.473	1020.04	2040.08
LPS group	8	0.620	1275.12	2550.25
LPS + DEX group	1	0.272	634.52	1269.04
LPS + DEX group	2	0.427	937.47	1874.95
LPS + DEX group	3	0.434	950.17	1900.35
LPS + DEX group	4	0.423	930.19	1860.39
LPS + DEX group	5	0.455	987.97	1975.94
LPS + DEX group	6	0.374	839.28	1678.56
LPS + DEX group	7	0.485	1041.27	2082.55
LPS + DEX group	10	0.380	850.60	1701.21
LPS + HZOL-L group	1	0.551	1156.47	2312.94
LPS + HZOL-L group	2	0.257	601.64	1203.28

LPS + HZOL-L group	3	0.387	863.74	1727.48
LPS + HZOL-L group	4	0.401	889.79	1779.58
LPS + HZOL-L group	5	0.374	839.28	1678.56
LPS + HZOL-L group	6	0.364	820.27	1640.54
LPS + HZOL-L group	7	0.382	854.36	1708.73
LPS + HZOL-L group	8	0.448	975.42	1950.84
LPS + HZOL-M group	1	0.263	614.91	1229.82
LPS + HZOL-M group	2	0.268	625.84	1251.69
LPS + HZOL-M group	3	0.179	408.50	817.00
LPS + HZOL-M group	4	0.242	567.72	1135.43
LPS + HZOL-M group	5	0.125	225.11	450.23
LPS + HZOL-M group	6	0.226	530.17	1060.34
LPS + HZOL-M group	7	0.213	498.44	996.88
LPS + HZOL-M group	8	0.297	687.38	1374.76
LPS + HZOL-H group	1	0.248	581.42	1162.84
LPS + HZOL-H group	2	0.215	503.40	1006.80
LPS + HZOL-H group	3	0.253	592.70	1185.40
LPS + HZOL-H group	4	0.293	679.07	1358.13
LPS + HZOL-H group	5	0.132	254.13	508.27
LPS + HZOL-H group	6	0.139	280.89	561.77
LPS + HZOL-H group	7	0.189	436.20	872.40
LPS + HZOL-H group	8	0.220	515.68	1031.35

Note 1. The dilution factor of the samples to be tested was 2 times.

Note 2. Diluted concentration was calculated by the help of ELISACalc.exe software according to the standard curve in Table S8-9.

Table S4-11: Standard curve of IL-1β in bronchoalveolar lavage fluid drawing by the help of ELISACalc.exe software.

Groups	Numbers	Absorbance value	Corrected absorbance value	Diluted concentration (pg/mL)
standards sample	1	0.024	0.000	0
standards sample	2	0.035	0.011	31.25
standards sample	3	0.052	0.028	62.5
standards sample	4	0.126	0.102	125
standards sample	5	0.245	0.221	250
standards sample	6	0.472	0.448	500
standards sample	7	1.167	1.143	1000
standards sample	8	2.366	2.342	2000

Table S4-12: Data of absorbance value and its concentration of IL-1β in bronchoalveolar lavage fluid.

Groups	Numbers	Absorbance value	Diluted concentration (pg/mL)	Actual concentration (pg/mL)
control group	1	0.134	159.82	319.63
control group	2	0.254	282.45	564.90
control group	3	0.218	247.88	495.77
control group	4	0.338	358.67	717.33
control group	5	0.248	276.79	553.57
control group	6	0.256	284.33	568.66
control group	7	0.262	289.95	579.90
control group	8	0.150	177.68	355.36
LPS group	1	1.089	950.31	1900.62
LPS group	2	0.881	792.21	1584.42
LPS group	3	0.894	802.11	1604.22
LPS group	4	0.872	785.35	1570.70
LPS group	5	0.937	834.83	1669.65
LPS group	6	0.773	709.61	1419.22
LPS group	7	0.997	880.42	1760.83
LPS group	8	0.784	718.06	1436.11
LPS + DEX group	1	0.696	650.17	1300.34
LPS + DEX group	2	0.517	508.76	1017.52
LPS + DEX group	3	0.799	729.56	1459.12
LPS + DEX group	4	0.828	751.76	1503.52
LPS + DEX group	5	0.773	709.61	1419.22
LPS + DEX group	6	0.751	692.69	1385.37
LPS + DEX group	7	0.788	721.13	1442.25
LPS + DEX group	10	0.923	824.18	1648.36
LPS + HZOL-L group	1	0.529	518.44	1036.88
LPS + HZOL-L group	2	0.542	528.89	1057.77
LPS + HZOL-L group	3	0.322	344.53	689.07
LPS + HZOL-L group	4	0.481	479.49	958.98
LPS + HZOL-L group	5	0.161	189.60	379.21
LPS + HZOL-L group	6	0.442	447.34	894.69
LPS + HZOL-L group	7	0.410	420.57	841.13
LPS + HZOL-L group	8	0.608	581.36	1162.72
LPS + HZOL-M group	1	0.496	491.73	983.46
LPS + HZOL-M group	2	0.416	425.62	851.23
LPS + HZOL-M group	3	0.506	499.85	999.71
LPS + HZOL-M group	4	0.599	574.26	1148.51
LPS + HZOL-M group	5	0.185	214.78	429.56
LPS + HZOL-M group	6	0.208	238.01	476.03
LPS + HZOL-M group	7	0.349	368.29	736.59
LPS + HZOL-M group	8	0.428	435.68	871.35

LPS + HZOL-H group	1	0.268	295.54	591.08
LPS + HZOL-H group	2	0.347	366.55	733.10
LPS + HZOL-H group	3	0.316	339.19	678.39
LPS + HZOL-H group	4	0.353	371.78	743.56
LPS + HZOL-H group	5	0.259	287.15	574.29
LPS + HZOL-H group	6	0.213	242.96	485.93
LPS + HZOL-H group	7	0.213	242.96	485.93
LPS + HZOL-H group	8	0.193	222.95	445.90

Note 1. The dilution factor of the samples to be tested was 2 times.

Note 2. Diluted concentration was calculated by the help of ELISACalc.exe software according to the standard curve in Table S8-11.

Table S4-13: Standard curve of TNF-α in bronchoalveolar lavage fluid drawing by the help of ELISACalc.exe software.

Groups	Numbers	Absorbance value	Corrected absorbance value	Diluted concentration (pg/mL)
standards sample	1	0.035	0.000	0
standards sample	2	0.069	0.034	82.3
standards sample	3	0.081	0.046	246.9
standards sample	4	0.116	0.081	740.7
standards sample	5	0.256	0.221	2222
standards sample	6	0.801	0.766	6667
standards sample	7	1.875	1.840	20000
standards sample	8	2.364	2.329	100000

Table S4-14: Data of absorbance value and its concentration of TNF-α in bronchoalveolar lavage fluid.

Groups	Numbers	Absorbance value	Diluted concentration (pg/mL)	Actual concentration (pg/mL)
control group	1	0.266	2590.33	5180.66
control group	2	0.206	2061.44	4122.89
control group	3	0.203	2033.17	4066.35
control group	4	0.165	1652.47	3304.95
control group	5	0.167	1673.77	3347.53
control group	6	0.230	2280.53	4561.06
control group	7	0.138	1345.47	2690.94
control group	8	0.143	1405.52	2811.04
LPS group	1	0.241	2377.30	4754.59
LPS group	2	0.336	3149.88	6299.75
LPS group	3	0.314	2978.70	5957.40

LPS group	4	0.330	3103.54	6207.09
LPS group	5	0.149	1475.46	2950.91
LPS group	6	0.192	1927.55	3855.10
LPS group	7	0.184	1848.60	3697.21
LPS group	8	0.221	2199.75	4399.49
LPS + DEX group	1	0.200	2004.68	4009.36
LPS + DEX group	2	0.174	1747.03	3494.06
LPS + DEX group	3	0.165	1652.47	3304.95
LPS + DEX group	4	0.151	1498.30	2996.59
LPS + DEX group	5	0.156	1554.46	3108.92
LPS + DEX group	6	0.140	1369.70	2739.39
LPS + DEX group	7	0.110	967.42	1934.85
LPS + DEX group	10	0.145	1429.08	2858.15
LPS + HZOL-L group	1	0.252	2472.12	4944.25
LPS + HZOL-L group	2	0.202	2023.70	4047.40
LPS + HZOL-L group	3	0.174	1747.03	3494.06
LPS + HZOL-L group	4	0.156	1554.46	3108.92
LPS + HZOL-L group	5	0.202	2023.70	4047.40
LPS + HZOL-L group	6	0.169	1694.90	3389.79
LPS + HZOL-L group	7	0.179	1798.24	3596.49
LPS + HZOL-L group	8	0.187	1878.44	3756.87
LPS + HZOL-M group	1	0.158	1576.57	3153.13
LPS + HZOL-M group	2	0.192	1927.55	3855.10
LPS + HZOL-M group	3	0.182	1828.56	3657.12
LPS + HZOL-M group	4	0.123	1153.45	2306.90
LPS + HZOL-M group	5	0.156	1554.46	3108.92
LPS + HZOL-M group	6	0.162	1620.21	3240.43
LPS + HZOL-M group	7	0.221	2199.75	4399.49
LPS + HZOL-M group	8	0.221	2199.75	4399.49
LPS + HZOL-H group	1	0.238	2351.11	4702.21
LPS + HZOL-H group	2	0.264	2573.60	5147.20
LPS + HZOL-H group	3	0.143	1405.52	2811.04
LPS + HZOL-H group	4	0.138	1345.47	2690.94
LPS + HZOL-H group	5	0.125	1180.25	2360.50
LPS + HZOL-H group	6	0.121	1126.22	2252.45
LPS + HZOL-H group	7	0.162	1620.21	3240.43
LPS + HZOL-H group	8	0.169	1694.90	3389.79

Note 1. The dilution factor of the samples to be tested was 2 times.

Note 2. Diluted concentration was calculated by the help of ELISACalc.exe software according to the standard curve in Table S8-13.

Table S4-15: Standard curve of IFN-γ in bronchoalveolar lavage fluid drawing by the help of ELISACalc.exe software.

Groups	Numbers	Absorbance value	Corrected absorbance value	Diluted concentration (pg/mL)
standards sample	1	0.043	0.000	0
standards sample	2	0.066	0.023	122.9
standards sample	3	0.131	0.088	307.2
standards sample	4	0.429	0.386	768
standards sample	5	0.799	0.756	1920
standards sample	6	1.383	1.340	4800
standards sample	7	1.958	1.915	12000
standards sample	8	2.405	2.362	30000

Table S4-16: Data of absorbance value and its concentration of IFN-γ in bronchoalveolar lavage fluid.

Groups	Numbers	Absorbance value	Diluted concentration (pg/mL)	Actual concentration (pg/mL)
control group	1	0.558	1186.50	2372.99
control group	2	0.691	1575.71	3151.43
control group	3	0.662	1486.56	2973.12
control group	4	0.683	1550.87	3101.73
control group	5	0.488	1000.36	2000.71
control group	6	0.523	1091.93	2183.86
control group	7	0.458	924.15	1848.30
control group	8	0.526	1099.92	2199.83
LPS group	1	0.979	2620.35	5240.70
LPS group	2	1.012	2762.12	5524.23
LPS group	3	0.973	2595.13	5190.27
LPS group	4	0.877	2213.61	4427.22
LPS group	5	0.884	2240.10	4480.21
LPS group	6	0.973	2595.13	5190.27
LPS group	7	0.843	2087.68	4175.36
LPS group	8	0.950	2500.03	5000.07
LPS + DEX group	1	0.581	1250.34	2500.68
LPS + DEX group	2	0.535	1124.01	2248.02
LPS + DEX group	3	0.517	1076.02	2152.04
LPS + DEX group	4	0.488	1000.36	2000.71
LPS + DEX group	5	0.498	1026.22	2052.44
LPS + DEX group	6	0.463	936.71	1873.42
LPS + DEX group	7	0.381	737.69	1475.37
LPS + DEX group	10	0.473	962.00	1923.99
LPS + HZOL-L group	1	0.576	1236.34	2472.69

LPS + HZOL-L group	2	0.493	1013.26	2026.52
LPS + HZOL-L group	3	0.438	874.48	1748.96
LPS + HZOL-L group	4	0.397	775.39	1550.78
LPS + HZOL-L group	5	0.493	1013.26	2026.52
LPS + HZOL-L group	6	0.428	849.98	1699.95
LPS + HZOL-L group	7	0.448	899.20	1798.41
LPS + HZOL-L group	8	0.463	936.71	1873.42
LPS + HZOL-M group	1	0.502	1036.63	2073.27
LPS + HZOL-M group	2	0.567	1211.32	2422.63
LPS + HZOL-M group	3	0.549	1161.89	2323.77
LPS + HZOL-M group	4	0.418	825.69	1651.38
LPS + HZOL-M group	5	0.498	1026.22	2052.44
LPS + HZOL-M group	6	0.512	1062.83	2125.66
LPS + HZOL-M group	7	0.616	1350.18	2700.35
LPS + HZOL-M group	8	0.616	1350.18	2700.35
LPS + HZOL-H group	1	0.641	1423.55	2847.10
LPS + HZOL-H group	2	0.612	1338.60	2677.20
LPS + HZOL-H group	3	0.563	1200.26	2400.52
LPS + HZOL-H group	4	0.478	974.72	1949.45
LPS + HZOL-H group	5	0.581	1250.34	2500.68
LPS + HZOL-H group	6	0.486	995.21	1990.42
LPS + HZOL-H group	7	0.512	1062.83	2125.66
LPS + HZOL-H group	8	0.526	1099.92	2199.83

Note 1. The dilution factor of the samples to be tested was 2 times.

Note 2. Diluted concentration was calculated by the help of ELISACalc.exe software according to the standard curve in Table S8-15.

Table S5: Data of RT-qPCR (from Table S5-1 to Table 5-6) and Western Blotting (Table S5-7).

Table S5-1: Data of RT-qPCR for three repeat in each group.

group	number	TLR4	CD14	MyD88	NF-κB p65
control group	1	1.00	1.00	1.00	1.00
control group	2	3.53	1.37	2.22	0.71
control group	3	3.79	1.23	3.87	0.51
LPS group	1	7.17	3.17	17.03	1.61
LPS group	2	6.92	7.68	11.17	2.37
LPS group	3	8.30	5.65	9.29	1.43
LPS + DEX group	1	4.20	1.40	3.13	0.80
LPS + DEX group	2	5.39	1.89	5.06	0.71
LPS + DEX group	3	4.51	0.98	1.53	0.61
LPS + HZOL-L group	1	6.34	3.97	9.44	0.92
LPS + HZOL-L group	2	6.19	3.68	7.35	0.66

LPS + HZOL-L group	3	5.02	2.29	6.45	1.36
LPS + HZOL-M group	1	6.05	1.72	9.42	0.71
LPS + HZOL-M group	2	5.84	1.50	5.41	1.06
LPS + HZOL-M group	3	5.40	3.05	2.64	0.61
LPS + HZOL-H group	1	5.52	2.15	3.81	0.73
LPS + HZOL-H group	2	5.71	1.36	4.91	0.80
LPS + HZOL-H group	3	3.89	1.17	2.12	0.53

Table S5-2: Information of RNA concentration.

Samples	Nucleic Acid (ng/uL)	A260/A280	Concentration of reverse transcriptional RNA(ng/uL)	Volume of reverse transcription RNA added(uL)	CDNA bulk solution volume (uL)
control 1	134.88	1.54	134.88	10.00	20.00
control 2	1677.85	1.81	200.00	10.00	20.00
control 3	1431.50	1.73	200.00	10.00	20.00
LPS 1	1004.85	1.75	200.00	10.00	20.00
LPS 2	1068.92	1.76	200.00	10.00	20.00
LPS 3	1195.99	1.73	200.00	10.00	20.00
LPS + DEX 1	1288.12	1.79	200.00	10.00	20.00
LPS + DEX 2	909.54	1.78	200.00	10.00	20.00
LPS + DEX 3	1734.46	1.87	200.00	10.00	20.00
LPS + HZOL-L 1	1262.41	1.82	200.00	10.00	20.00
LPS + HZOL-L 2	892.01	1.73	200.00	10.00	20.00
LPS + HZOL-L 3	1050.64	1.75	200.00	10.00	20.00
LPS + HZOL-M 1	1656.97	1.81	200.00	10.00	20.00
LPS + HZOL-M 2	939.74	1.76	200.00	10.00	20.00
LPS + HZOL-M 3	1091.26	1.79	200.00	10.00	20.00
LPS + HZOL-H 1	1167.90	1.77	200.00	10.00	20.00
LPS + HZOL-H 2	1487.77	1.83	200.00	10.00	20.00
LPS + HZOL-H 3	1404.16	1.83	200.00	10.00	20.00

Table S5-3: 2^-DDCt method calculation results of TLR4.

**Samples**	**CT data**		**Average of CT data**		**△CT=CT of TLR4-CT of GAPDH**	**△△CT=△CT sample-△CT control 1**	**Amplification multiple=2^-△△CT**
	GAPDH	TLR4	GAPDH	TLR4	TLR4	TLR4	TLR4
control 1-1	22.52	30.81					
control 1-2	22.69	32.34	22.61	31.76	9.15	0.00	1.00
control 1-3	22.62	32.12					
control 2-1	20.24	27.96					
control 2-2	20.27	27.31	20.24	27.57	7.33	-1.82	3.53

control 2-3	20.22	27.45					
control 3-1	16.29	23.47					
control 3-2	16.42	23.64	16.38	23.61	7.23	-1.92	3.79
control 3-3	16.44	23.73					
LPS 1-1	18.40	24.65					
LPS 1-2	18.39	24.65	18.33	24.63	6.31	-2.84	7.17
LPS 1-3	18.18	24.61					
LPS 2-1	18.16	24.49					
LPS 2-2	18.26	24.54	18.20	24.56	6.36	-2.79	6.92
LPS 2-3	18.19	24.66					
LPS 3-1	17.63	23.83					
LPS 3-2	17.61	23.71	17.64	23.73	6.10	-3.05	8.30
LPS 3-3	17.66	23.66					
LPS+DEX 1-1	20.03	27.24					
LPS+DEX 1-2	20.11	27.16	20.09	27.17	7.08	-2.07	4.20
LPS+DEX 1-3	20.12	27.11					
LPS+DEX 2-1	17.85	24.67					
LPS+DEX 2-2	18.06	24.63	17.91	24.63	6.72	-2.43	5.39
LPS+DEX 2-3	17.81	24.58					
LPS+DEX 3-1	19.20	26.20					
LPS+DEX 3-2	19.28	26.30	19.31	26.28	6.98	-2.17	4.51
LPS+DEX 3-3	19.44	26.35					
LPS+HZOL-L 1-1	19.04	25.65					
LPS+HZOL-L 1-2	19.03	25.67	19.16	25.64	6.49	-2.66	6.34
LPS+HZOL-L 1-3	19.40	25.61					
LPS+HZOL-L 2-1	18.12	24.75					
LPS+HZOL-L 2-2	18.26	24.83	18.30	24.82	6.52	-2.63	6.19
LPS+HZOL-L 2-3	18.54	24.89					
LPS+HZOL-L 3-1	17.59	24.47					
LPS+HZOL-L 3-2	17.73	24.36	17.62	24.45	6.82	-2.33	5.02
LPS+HZOL-L 3-3	17.54	24.50					
LPS+HZOL-M 1-1	17.86	24.52					
LPS+HZOL-M 1-2	18.19	24.56	17.99	24.55	6.55	-2.60	6.05
LPS+HZOL-M 1-3	17.92	24.56					
LPS+HZOL-M 2-1	18.51	25.06					
LPS+HZOL-M 2-2	18.51	25.16	18.55	25.15	6.60	-2.55	5.84
LPS+HZOL-M 2-3	18.62	25.24					
LPS+HZOL-M 3-1	18.96	25.43					
LPS+HZOL-M 3-2	18.68	25.51	18.75	25.46	6.72	-2.43	5.40
LPS+HZOL-M 3-3	18.60	25.44					
LPS+HZOL-H 1-1	19.94	26.60					
LPS+HZOL-H 1-2	20.09	26.90	20.08	26.76	6.69	-2.47	5.52
LPS+HZOL-H 1-3	20.20	26.79					

LPS+HZOL-H 2-1	19.88	26.72					
LPS+HZOL-H 2-2	20.08	26.52	19.99	26.63	6.64	-2.51	5.71
LPS+HZOL-H 2-3	20.01	26.64					
LPS+HZOL-H 3-1	20.33	27.50					
LPS+HZOL-H 3-2	20.30	27.47	20.30	27.49	7.19	-1.96	3.89
LPS+HZOL-H 3-3	20.27	27.51					

Table S5-4: 2^-DDCt method calculation results of CD14.

**Samples**	**CT data**		**Average of CT data**		**△CT=CT of CD14-CT of GAPDH**	**△△CT=△CT sample-△CT control 1**	**Amplification multiple=2^-△△CT**
	GAPDH	CD14	GAPDH	CD14	CD14	CD14	CD14
control 1-1	22.52	30.15					
control 1-2	22.69	29.55	22.61	29.69	7.08	0.00	1.00
control 1-3	22.62	29.36					
control 2-1	20.24	27.00					
control 2-2	20.27	26.87	20.24	26.87	6.63	-0.45	1.37
control 2-3	20.22	26.75					
control 3-1	18.40	25.70					
control 3-2	18.39	24.70	18.33	25.10	6.78	-0.30	1.23
control 3-3	18.18	24.91					
LPS 1-1	16.29	22.40					
LPS 1-2	16.42	22.41	16.38	21.80	5.42	-1.67	3.17
LPS 1-3	16.44	20.59					
LPS 2-1	18.16	21.26					
LPS 2-2	18.26	22.70	18.20	22.34	4.14	-2.94	7.68
LPS 2-3	18.19	23.07					
LPS 3-1	17.63	21.99					
LPS 3-2	17.61	22.92	17.64	22.22	4.58	-2.50	5.65
LPS 3-3	17.66	21.75					
LPS+DEX 1-1	20.03	26.57					
LPS+DEX 1-2	20.11	26.59	20.09	26.68	6.60	-0.49	1.40
LPS+DEX 1-3	20.12	26.89					
LPS+DEX 2-1	17.85	24.13					
LPS+DEX 2-2	18.06	24.10	17.91	24.07	6.17	-0.92	1.89
LPS+DEX 2-3	17.81	24.00					
LPS+DEX 3-1	19.20	26.39					
LPS+DEX 3-2	19.28	26.44	19.31	26.41	7.11	0.02	0.98
LPS+DEX 3-3	19.44	26.40					
LPS+HZOL-L 1-1	19.04	23.61					
LPS+HZOL-L 1-2	19.03	24.57	19.16	24.25	5.09	-1.99	3.97
LPS+HZOL-L 1-3	19.40	24.57					

LPS+HZOL-L 2-1	18.12	23.05					
LPS+HZOL-L 2-2	18.26	23.75	18.30	23.50	5.20	-1.88	3.68
LPS+HZOL-L 2-3	18.54	23.71					
LPS+HZOL-L 3-1	17.59	23.56					
LPS+HZOL-L 3-2	17.73	23.46	17.62	23.51	5.88	-1.20	2.29
LPS+HZOL-L 3-3	17.54	23.50					
LPS+HZOL-M 1-1	17.86	23.77					
LPS+HZOL-M 1-2	18.19	24.47	17.99	24.29	6.30	-0.78	1.72
LPS+HZOL-M 1-3	17.92	24.62					
LPS+HZOL-M 2-1	18.51	24.01					
LPS+HZOL-M 2-2	18.51	25.58	18.55	25.04	6.50	-0.59	1.50
LPS+HZOL-M 2-3	18.62	25.54					
LPS+HZOL-M 3-1	18.96	23.96					
LPS+HZOL-M 3-2	18.68	24.29	18.75	24.22	5.47	-1.61	3.05
LPS+HZOL-M 3-3	18.60	24.42					
LPS+HZOL-H 1-1	19.94	25.65					
LPS+HZOL-H 1-2	20.09	25.70	20.08	26.06	5.98	-1.10	2.15
LPS+HZOL-H 1-3	20.20	26.82					
LPS+HZOL-H 2-1	19.88	27.07					
LPS+HZOL-H 2-2	20.08	26.39	19.99	26.63	6.64	-0.44	1.36
LPS+HZOL-H 2-3	20.01	26.43					
LPS+HZOL-H 3-1	20.33	27.12					
LPS+HZOL-H 3-2	20.30	27.02	20.30	27.15	6.86	-0.23	1.17
LPS+HZOL-H 3-3	20.27	27.32					

Table S5-5: 2^-DDCt method calculation results of MyD88.

**Samples**	**CT data**		**Average of CT data**		**△ CT=CT of MyD88-CT of GAPDH**	**△△CT= △CT sample-△ CT control 1**	**Amplification multiple=2^-△△CT**
	GAPDH	MyD88	GAPDH	MyD88	MyD88	MyD88	MyD88
control 1-1	22.52	32.30					
control 1-2	22.69	31.32	22.61	32.30	9.69	0.00	1.00
control 1-3	22.62	33.28					
control 2-1	20.24	28.70					
control 2-2	20.27	28.88	20.24	28.78	8.54	-1.15	2.22
control 2-3	20.22	28.77					
control 3-1	18.40	25.87					

control 3-2	18.39	26.33	18.33	26.07	7.74	-1.95	3.87
control 3-3	18.18	26.01					
LPS 1-1	16.29	22.13					
LPS 1-2	16.42	22.11	16.38	21.99	5.60	-4.09	17.03
LPS 1-3	16.44	21.71					
LPS 2-1	18.16	24.36					
LPS 2-2	18.26	24.41	18.20	24.42	6.21	-3.48	11.17
LPS 2-3	18.19	24.48					
LPS 3-1	17.63	23.99					
LPS 3-2	17.61	24.16	17.64	24.11	6.48	-3.22	9.29
LPS 3-3	17.66	24.19					
LPS+DEX 1-1	20.03	28.22					
LPS+DEX 1-2	20.11	28.19	20.09	28.13	8.05	-1.65	3.13
LPS+DEX 1-3	20.12	27.99					
LPS+DEX 2-1	17.85	25.43					
LPS+DEX 2-2	18.06	25.00	17.91	25.26	7.35	-2.34	5.06
LPS+DEX 2-3	17.81	25.35					
LPS+DEX 3-1	19.20	28.40					
LPS+DEX 3-2	19.28	28.39	19.31	28.39	9.08	-0.61	1.53
LPS+DEX 3-3	19.44	28.38					
LPS+HZOL-L 1-1	19.04	25.62					
LPS+HZOL-L 1-2	19.03	25.87	19.16	25.61	6.45	-3.24	9.44
LPS+HZOL-L 1-3	19.40	25.35					
LPS+HZOL-L 2-1	18.12	25.51					
LPS+HZOL-L 2-2	18.26	24.89	18.30	25.12	6.82	-2.88	7.35
LPS+HZOL-L 2-3	18.54	24.96					
LPS+HZOL-L 3-1	17.59	25.38					
LPS+HZOL-L 3-2	17.73	24.45	17.62	24.63	7.01	-2.69	6.45
LPS+HZOL-L 3-3	17.54	24.05					
LPS+HZOL-M 1-1	17.86	23.88					
LPS+HZOL-M 1-2	18.19	24.77	17.99	24.45	6.46	-3.24	9.42
LPS+HZOL-M 1-3	17.92	24.69					
LPS+HZOL-M 2-1	18.51	25.47					
LPS+HZOL-M 2-2	18.51	25.70	18.55	25.81	7.26	-2.44	5.41
LPS+HZOL-M 2-3	18.62	26.25					
LPS+HZOL-M 3-1	18.96	27.03					
LPS+HZOL-M 3-2	18.68	26.89	18.75	27.04	8.29	-1.40	2.64
LPS+HZOL-M 3-3	18.60	27.19					
LPS+HZOL-H 1-1	19.94	28.08					
LPS+HZOL-H 1-2	20.09	27.45	20.08	27.84	7.76	-1.93	3.81
LPS+HZOL-H 1-3	20.20	27.99					
LPS+HZOL-H 2-1	19.88	27.52					
LPS+HZOL-H 2-2	20.08	27.42	19.99	27.39	7.40	-2.30	4.91

LPS+HZOL-H 2-3	20.01	27.23					
LPS+HZOL-H 3-1	20.33	28.85					
LPS+HZOL-H 3-2	20.30	29.03	20.30	28.91	8.61	-1.08	2.12
LPS+HZOL-H 3-3	20.27	28.85					

Table S5-6: 2^-DDCt method calculation results of NF-κB p65.

**Samples**	**CT data**		**Average of CT data**		**△ CT=CT of MyD88-CT of GAPDH**	**△△CT=△CT sample-△CT control 1**	**Amplification multiple=2^-△△CT**
	GAPDH	NF-κB p65	GAPDH	NF-κB p65	NF-κB p65	NF-κB p65	NF-κB p65
control 1-1	22.52	29.03					
control 1-2	22.69	28.72	22.61	28.80	6.19	0.00	1.00
control 1-3	22.62	28.65					
control 2-1	20.24	26.69					
control 2-2	20.27	27.10	20.24	26.93	6.69	0.49	0.71
control 2-3	20.22	27.01					
control 3-1	18.40	25.52					
control 3-2	18.39	25.51	18.33	25.50	7.17	0.98	0.51
control 3-3	18.18	25.46					
LPS 1-1	16.29	21.84					
LPS 1-2	16.42	22.04	16.38	21.89	5.51	-0.68	1.61
LPS 1-3	16.44	21.80					
LPS 2-1	18.16	23.16					
LPS 2-2	18.26	23.25	18.20	23.15	4.95	-1.25	2.37
LPS 2-3	18.19	23.05					
LPS 3-1	17.63	23.15					
LPS 3-2	17.61	23.79	17.64	23.31	5.68	-0.52	1.43
LPS 3-3	17.66	23.00					
LPS+DEX 1-1	20.03	26.05					
LPS+DEX 1-2	20.11	26.96	20.09	26.60	6.51	0.32	0.80
LPS+DEX 1-3	20.12	26.78					
LPS+DEX 2-1	17.85	24.47					
LPS+DEX 2-2	18.06	24.47	17.91	24.60	6.69	0.50	0.71
LPS+DEX 2-3	17.81	24.86					
LPS+DEX 3-1	19.20	26.09					
LPS+DEX 3-2	19.28	26.27	19.31	26.22	6.91	0.72	0.61
LPS+DEX 3-3	19.44	26.30					
LPS+HZOL-L 1-1	19.04	25.76					
LPS+HZOL-L 1-2	19.03	25.21	19.16	25.48	6.32	0.13	0.92

LPS+HZOL-L 1-3	19.40	25.47					
LPS+HZOL-L 2-1	18.12	25.46					
LPS+HZOL-L 2-2	18.26	25.03	18.30	25.10	6.80	0.60	0.66
LPS+HZOL-L 2-3	18.54	24.82					
LPS+HZOL-L 3-1	17.59	23.44					
LPS+HZOL-L 3-2	17.73	23.26	17.62	23.37	5.75	-0.45	1.36
LPS+HZOL-L 3-3	17.54	23.41					
LPS+HZOL-M 1-1	17.86	23.95					
LPS+HZOL-M 1-2	18.19	25.10	17.99	24.68	6.69	0.50	0.71
LPS+HZOL-M 1-3	17.92	25.01					
LPS+HZOL-M 2-1	18.51	24.62					
LPS+HZOL-M 2-2	18.51	24.62	18.55	24.65	6.11	-0.09	1.06
LPS+HZOL-M 2-3	18.62	24.72					
LPS+HZOL-M 3-1	18.96	25.47					
LPS+HZOL-M 3-2	18.68	25.69	18.75	25.65	6.90	0.71	0.61
LPS+HZOL-M 3-3	18.60	25.79					
LPS+HZOL-H 1-1	19.94	26.44					
LPS+HZOL-H 1-2	20.09	26.87	20.08	26.72	6.64	0.44	0.73
LPS+HZOL-H 1-3	20.20	26.84					
LPS+HZOL-H 2-1	19.88	26.52					
LPS+HZOL-H 2-2	20.08	26.50	19.99	26.51	6.52	0.32	0.80
LPS+HZOL-H 2-3	20.01	26.50					
LPS+HZOL-H 3-1	20.33	27.47					
LPS+HZOL-H 3-2	20.30	27.49	20.30	27.40	7.10	0.91	0.53
LPS+HZOL-H 3-3	20.27	27.26					

Table S5-7: Data of western blotting results for three repeat in each group.

**group**	**number**	**TLR4**	**CD14**	**MyD88**	**p-NF-κB p65/ NF-κB p65 ratio**
control group	1	0.0449	0.0630	0.1667	0.7918
control group	2	0.0555	0.0411	0.0576	0.3102
control group	3	0.0411	0.0403	0.1012	1.4315
LPS group	1	0.1461	0.1720	0.3596	2.4530
LPS group	2	0.1718	0.0882	0.2370	2.3137
LPS group	3	0.1511	0.1248	0.4104	3.8798
LPS + DEX group	1	0.0715	0.0577	0.1798	1.2778
LPS + DEX group	2	0.1014	0.0536	0.0490	0.8410
LPS + DEX group	3	0.0511	0.0666	0.1920	1.5475
LPS + HZOL-L group	1	0.1125	0.0678	0.2489	1.3370
LPS + HZOL-L group	2	0.1022	0.0697	0.0670	1.0723

LPS + HZOL-L group	3	0.0877	0.0770	0.1685	2.2301
LPS + HZOL-M group	1	0.1066	0.0533	0.2791	0.9965
LPS + HZOL-M group	2	0.0848	0.0935	0.0905	1.7511
LPS + HZOL-M group	3	0.1345	0.0750	0.1255	1.9764
LPS + HZOL-H group	1	0.0753	0.0497	0.1654	1.1167
LPS + HZOL-H group	2	0.1206	0.0841	0.0803	0.6286
LPS + HZOL-H group	3	0.1046	0.0259	0.1543	2.3300

Figure S4: PCR amplification curve and melting curve.

Figure S4-1: PCR amplification curve of (1) TLR4, (2) CD14, (3)MyD88, and (4) NF-κB p65.

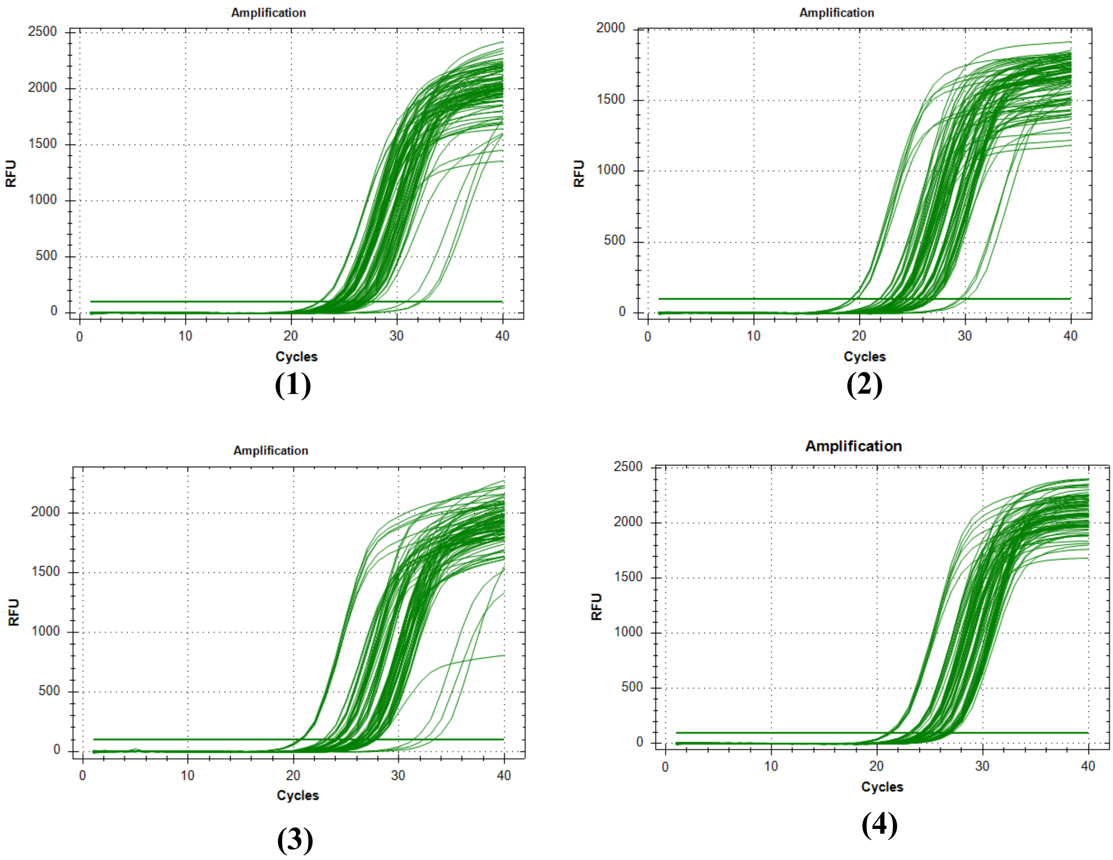

Figure S4-2: PCR melting curve of (1) TLR4, (2) CD14, (3)MyD88, and (4) NF-κB p65.

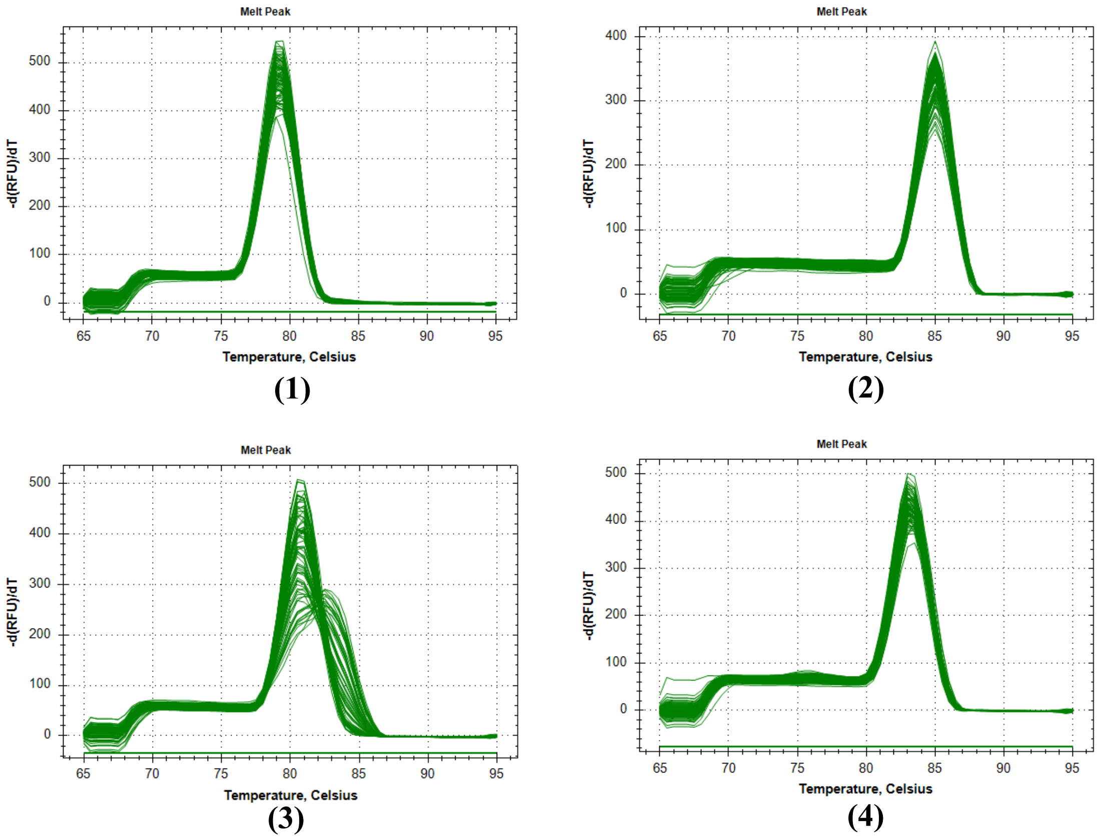

Figure S5: Original images of Western Blotting for three repeats in each group.

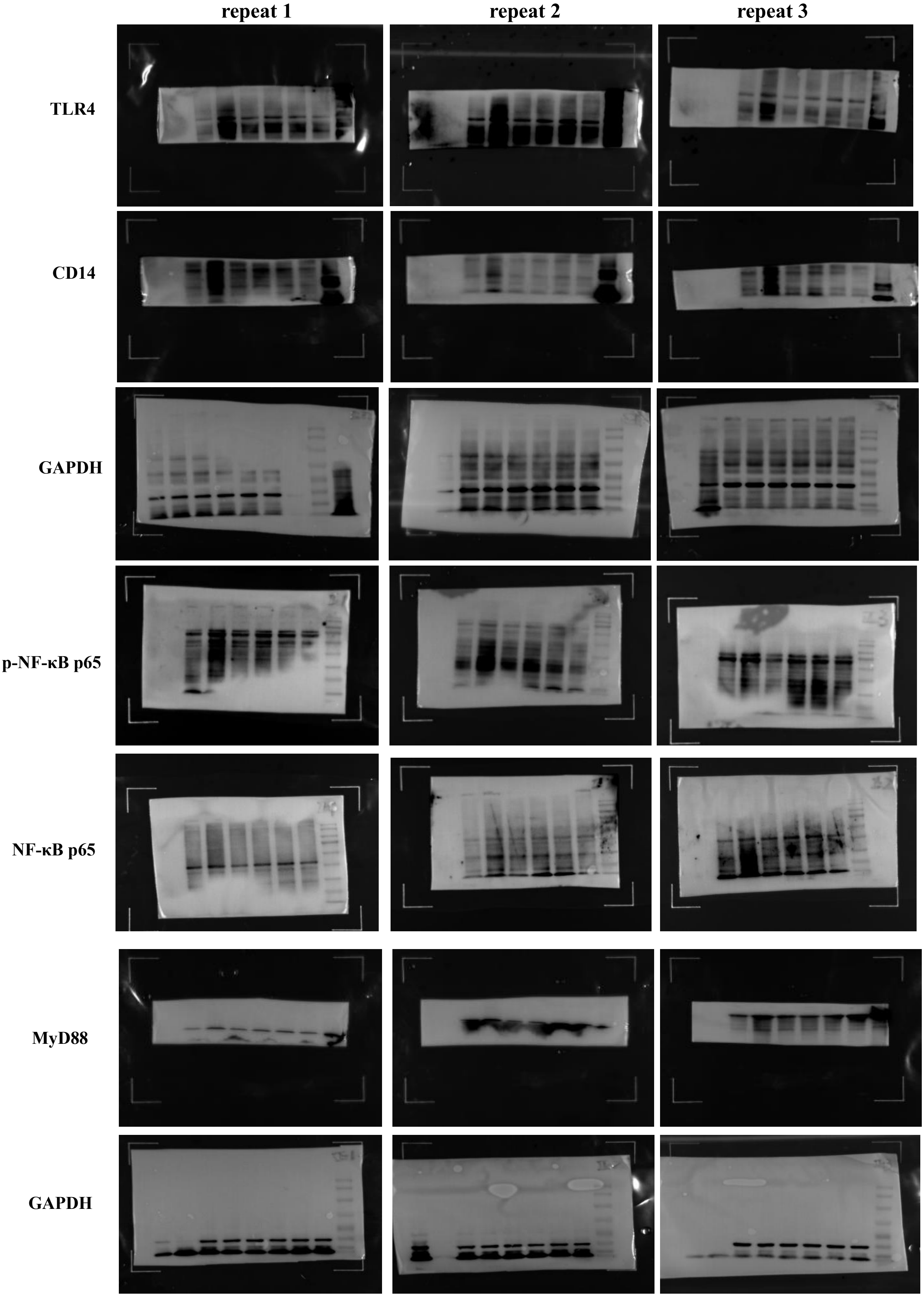

Supplement: Supplementary Materials — Supplementary Material (1): all the data of network pharmacology analysis. Supplementary Material (2): all the data of experimental validation results. [file 6183551.f1.zip › Supplementary Material (2).pdf]
